# Stabilities of Ac^3+^ Complexes Relevant as Radiopharmaceuticals

**DOI:** 10.1021/acs.inorgchem.5c05846

**Published:** 2026-02-24

**Authors:** Antía Freire-García, Raúl Alvarado, María Costa-DeDios, David Esteban-Gómez, Carlos Platas-Iglesias

**Affiliations:** CICA—Centro Interdisciplinar de Química e Bioloxía and Departamento de Química, Facultade de Ciencias, 16737 Universidade da Coruña, 15071 A Coruña, Galicia, Spain

## Abstract

We present a detailed density functional theory (DFT) investigation of the structural features and thermodynamic stabilities of La^3+^ and Ac^3+^ complexes relevant to develop radiopharmaceutical agents. A total of 16 chelators were considered, covering the acyclic and macrocyclic families functionalized with different numbers and types of donor atoms. The bond distances of the Ac^3+^ coordination environment are systematically longer than those obtained for the La^3+^ analogues, which allowed us to estimate an ionic radius for Ac^3+^ in coordination number 9 of 1.275 ± 0.020 Å (1.216 and 1.206 Å were proposed for La^3+^). Energy decomposition analysis (EDA) provided hints into the nature of the metal–ligand interactions and their relative weight in La^3+^ and Ac^3+^ complexes. A thermodynamic DFT study allowed us to estimate the stability constants of the Ac^3+^ complexes from those of the La^3+^ ones, as for the latter experimental values are available in the literature. These studies evidenced that Ac^3+^ tends to form complexes with lower thermodynamic stability in comparison with La^3+^, with the exception of one of the leading chelators for Ac^3+^, MACROPA^2–^, and the unexpected case of OCTAPA^4–^. Overall, the methodology reported here will allow identifying chelators well suited for Ac^3+^ coordination, thereby aiding the design of radiopharmaceuticals based on [^225^Ac]­Ac^3+^ for targeted α therapy (TAT).

## Graphical abstract

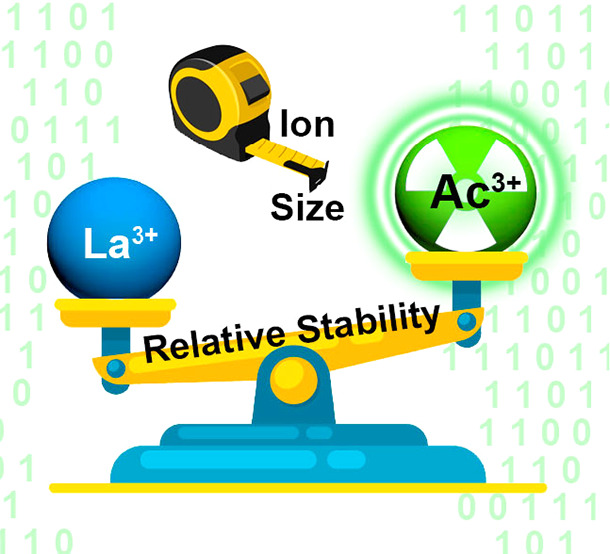

## Introduction

Actinium is the element that heads the actinide series, which comprises the 15 elements with atomic numbers ranging from 89 (Ac) to 103 (Lr). In spite of having more than 30 known isotopes,^1^ none of them are stable and only two are found naturally: ^227^Ac (*t*_1/2_ = 21.8 years) and ^228^Ac (*t*_1/2_ = 6.1 h), which come from the decay of ^235^U and ^232^Th, respectively.^2^ One of the synthetic radioisotopes of Ac, ^225^Ac, is one of the most promising candidates for targeted α therapy (TAT) due to its half-life of *t*_1/2_ = 9.92 days, as well as for its decay to the six predominant radionuclide daughters through four high-energy α and two β disintegrations, to reach stable ^209^Bi.^3,4^ The decay through multiple α emissions makes ^225^Ac a very powerful cytotoxic agent.^5^

The [Rn]­6d^1^7s^2^ electron configuration of actinium results in a coordination chemistry restricted to the trivalent Ac^3+^ ion, which has a [Rn]­5f^0^ configuration and thus is silent in optical spectroscopy, in contrast to other ions of the actinide or lanthanide^6,7^ series with partially filled 5f (or 4f) orbitals. This, together with the lack of stable isotopes, limited the number of studies on the coordination chemistry of Ac^3+^, which remains largely unexplored.^8^ Nevertheless, some important progress has been made in the past few years. For instance, extended X-ray absorption fine structure (EXAFS) and computational studies revealed that the Ac^3+^ aqua ion contains nine water molecules in the inner coordination sphere,^9–12^ as observed for La^3+^ in the solid state.^13^ More recently, the use of the long-lived ^227^Ac isotope allowed to obtain the X-ray crystal structure of the Ac^3+^-HOPO complex within the binding pocket of siderocalin, a siderophore-seeking protein.^14^ Mass spectrometry has been also recently studied to characterize different Ac^3+^ complexes formed in the presence of H_2_O and N_2_.^15^ However, the limited quantities of [^227^Ac]­Ac^3+^ available and the lack of stable isotopes precludes the use of most of the analytical and spectroscopic techniques commonly employed in coordination chemistry. As a result, computational studies are extremely important to investigate Ac^3+^ complexes.^16,17^ Furthermore, the coordination chemistry of Ac^3+^ is generally inferred from that of La^3+^, as these two ions are believed to have relatively similar coordination properties, due to their identical charge and similar ionic radii. Indeed, the ionic radius of Ac^3+^ for coordination number (CN) 9 was estimated to be 1.220 Å,^8,18^ while the corresponding values for La^3+^ proposed by Shannon and Persson are 1.216 and 1.206 Å, respectively.^18,19^ Nevertheless, computational studies are still needed to establish the similarities and differences between La^3+^ and Ac^3+^.

The application of ^225^Ac in TAT requires the use of bifunctional chelators, which must offer strong complexation of the [^225^Ac]­Ac^3+^ ion, while containing a functional group that allows conjugation to the targeting unit (i.e., an antibody or a peptide).^3,4,20^ This ensures that the radioactivity is delivered selectively to the tumor, minimizing the exposure of healthy tissue to radiation. The lack of chelators well-suited for [^225^Ac]­Ac^3+^ hindered the development of ^225^Ac-TAT for some time. Initial attempts toward bifunctional chelators of ^225^Ac relied on DOTA,^21–23^ which is considered the gold standard for the coordination of lanthanide and other trivalent ions, with some derivatives reaching clinical trials.^24^ However, some reports demonstrated that the [^225^Ac]­Ac^3+^-DOTA complex experiences slight dissociation in vivo.^25^ Additionally, the radiolabeling of DOTA derivatives with [^225^Ac]­Ac^3+^ often requires mild heating due to slow chelation kinetics, which prompted the development of alternative chelators.

A breakthrough in the coordination chemistry of Ac^3+^ was reported in 2017 by Wilson, who discovered that the macrocyclic chelator MACROPA^2–^ has excellent properties for TAT with [^225^Ac]­Ac^3+^,^26^ taking inspiration on previous studies that demonstrated that this chelator is highly selective for large metal ions.^27,28^ In subsequent studies, MACROPA^2–^ was subjected to different modifications to tune its selectivity and other properties.^29–33^ Bifunctional derivatives of MACROPA^2–^ have been prepared and demonstrated to have very promising properties,^34–37^ which paved the way to start clinical trials. In spite of the success of MACROPA^2–^, the search for chelators for ^225^Ac-TAT remains a hot topic, as the nature of the chelator may affect significantly the performance of the conjugate. A recent study highlighted PYTA^4–^ as a promising chelator for [^225^Ac]­Ac^3+^.^38^ Different chelators containing picolinate groups were also found to display favorable properties.^39–41^

In this work we report a computational study that analyzes a series of chelators with potential for ^225^Ac-TAT applications. We present a detailed comparison of the structures of the Ac^3+^ and La^3+^ derivatives to investigate the similarities and differences among them in terms of structures and chemical bonding. We also developed a computational methodology that allows estimating the thermodynamic stability of the Ac^3+^ complexes using stability data reported for the La^3+^ analogues. Energy decomposition analysis (EDA) was performed in order to identify and understand the differences in behavior of the La^3+^ and Ac^3+^ complexes. The chelators chosen for this study involve both macrocyclic and acyclic structures, in which factors like denticity, cavity size and rigidity are varied (Figure 1). Furthermore, the La^3+^ complexes of these chelators have been characterized in most cases, so that thermodynamic and structural data are available.

**Figure 1:**
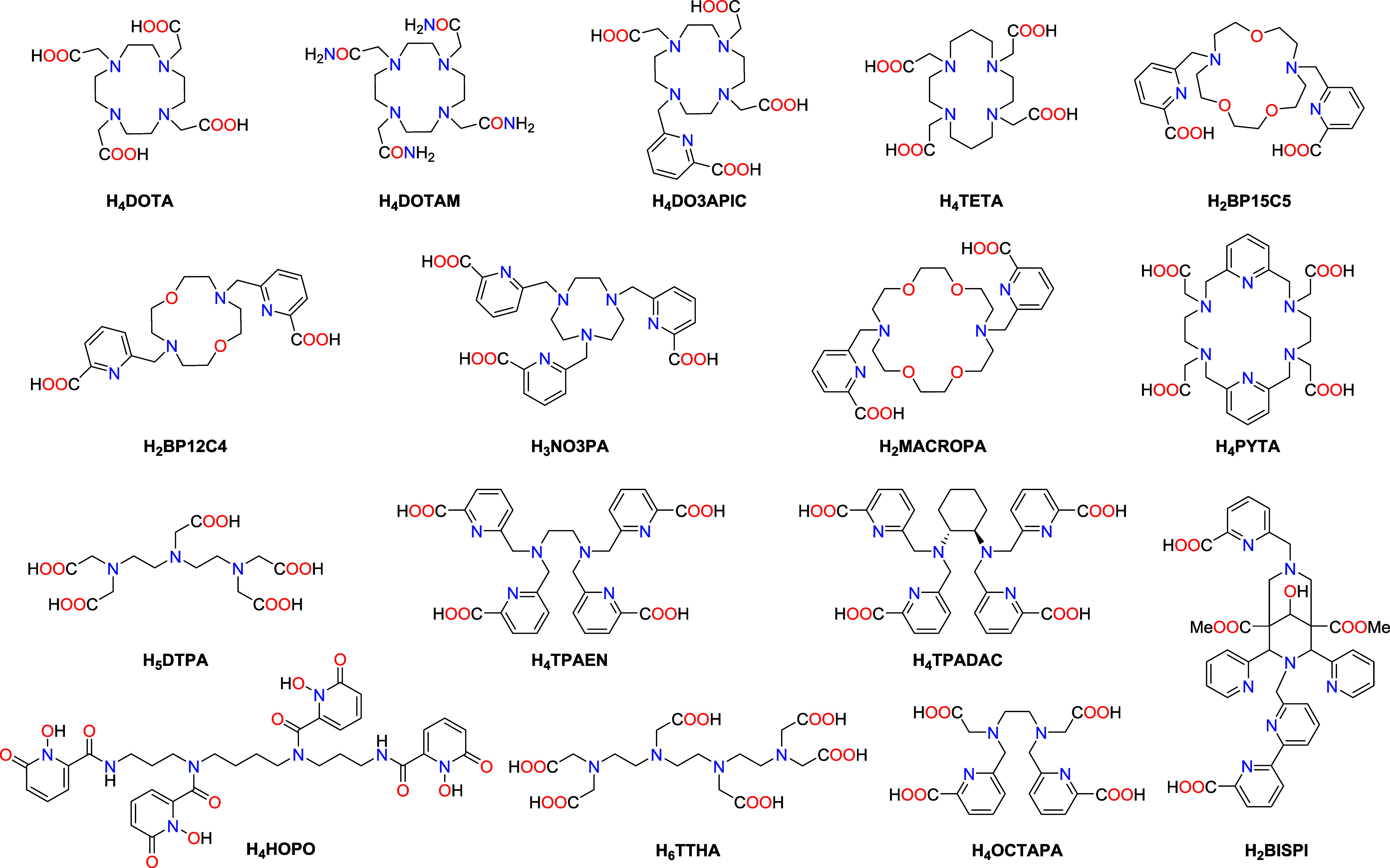
Structures of ligands discussed in this work.

## Results and Discussion

### Structural features

The structures of the La^3+^ complexes of the chelators shown in Figure 1 were optimized using the TPSSh functional, which was shown to provide better geometries than popular functionals like B3LYP for lanthanide complexes.^42^ The details of the methodology used are provided in the Computational Details section below. When available, single-crystal X-ray data of the La^3+^ complexes were used to generate input geometries for optimization (TPAEN^4–^ and TPADAC^4–^,^43^ MACROPA^2–^,^26^ PYTA^4–^,^44^ OCTAPA^4–^,^45^ DOTA^4–^,^46^ TTHA^6–^ ^47^ and DTPA^5–^ ^48^). A X-ray crystal structure reported for the La^3+^ complex of DOTAM contains a ten-coordinated metal ion, where an ethanol molecule and a triflate anion are present in the inner coordination environment.^49^ However, solution NMR and conductivity studies suggested that a nine-coordinated structure similar to those of DOTA^4–^ complexes is likely present in solution, and thus we modeled the [M­(DOTAM)­(H_2_O)]^3+^ systems (M = La or Ac). Of note, the minimum energy structures obtained for the Ac^3+^ and La^3+^ complexes of DOTA^4–^ and DOTAM correspond to the twisted-square antiprismatic (TSAP) isomers, in agreement with solution ^1^H NMR studies on the La^3+^ complexes^50^ and previous computational work on actinide complexes.^16,51^ Similarly, our calculations provided a minimum energy conformation for the complexes of MACROPA^2–^ corresponding to the Δ­(δλδ)­(δλδ) isomers, in line with previous computational work.^17,27^

A comparison of the experimental and calculated bond lengths of the La^3+^ coordination environment is presented in Tables S1–S17, Supporting Information. The distances of the La^3+^ coordination environment found in the optimized geometries show an excellent agreement with the X-ray data, with absolute mean deviations of 0.027 and 0.038 Å for the La–N and Ln–O bonds, respectively. The corresponding root-mean-square deviations (RMSD) for La–N and Ln–O bonds are 0.035 and 0.052, with the overall RMSD amounting to 0.044. Significantly higher RMSD values were reported (∼0.1), and considered to be acceptable, in previous computational studies on Ln^3+^ complexes.^52,53^

Figure 2 shows the differences in bond distances of the metal coordination environments, calculated for equivalent donor atoms, between the La^3+^ and Ac^3+^ complexes, Δ*d*_M‑D_ = *d*_Ac‑D_ – *d*_La‑D_. The values of Δ*d*_M‑D_ are all positive, indicating that the distances of the metal coordination environment are systematically longer for the Ac^3+^ complexes in comparison with the La^3+^ analogues. The histogram representing the differences for all donor atoms, in the 17 pairs of complexes studied, display a double Gaussian distribution. We have previously noted that the Ln–N bonds involving amine N atoms are characterized by rather flat potential energy surfaces, which results in rather broad distributions of their bond distances.^54^ This is also the reason why DFT often overestimates these distances with respect to those observed by X-ray.^42^ Thus, the histogram involving all donor atoms except the amine N atoms indeed displays a distribution that can be represented by a single Gaussian, while the histogram representing the Δ*d*_M‑D_ values for amine N atoms is quite broad and presents a maximum that coincides with the maximum with the highest Δ*d*_M‑D_ value for the histogram containing all data. Therefore, the histogram displaying Δ*d*_M‑D_ values involving all donor atoms except amines reflects better the differences in ionic radius between La^3+^ and Ac^3+^. The Gaussian distribution for the latter data is centered at 0.059 ± 0.001 Å, which allows us to estimate an ionic radius of Ac^3+^ for CN = 9 of 1.275 ± 0.020 Å from the reliable value reported by Shannon for La^3+^ (1.216 Å).^19,55^ Of note, the complexes considered in this study display nine- or ten-coordinated metal ions, with the complex of MACROPA reaching coordination number 11. Nevertheless, we expect that these high coordination numbers provide comparable Δ*d*_M‑D_ values, as the ionic radii of La^3+^ and Ac^3+^ very likely vary in a similar way upon increasing coordination number.

**Figure 2:**
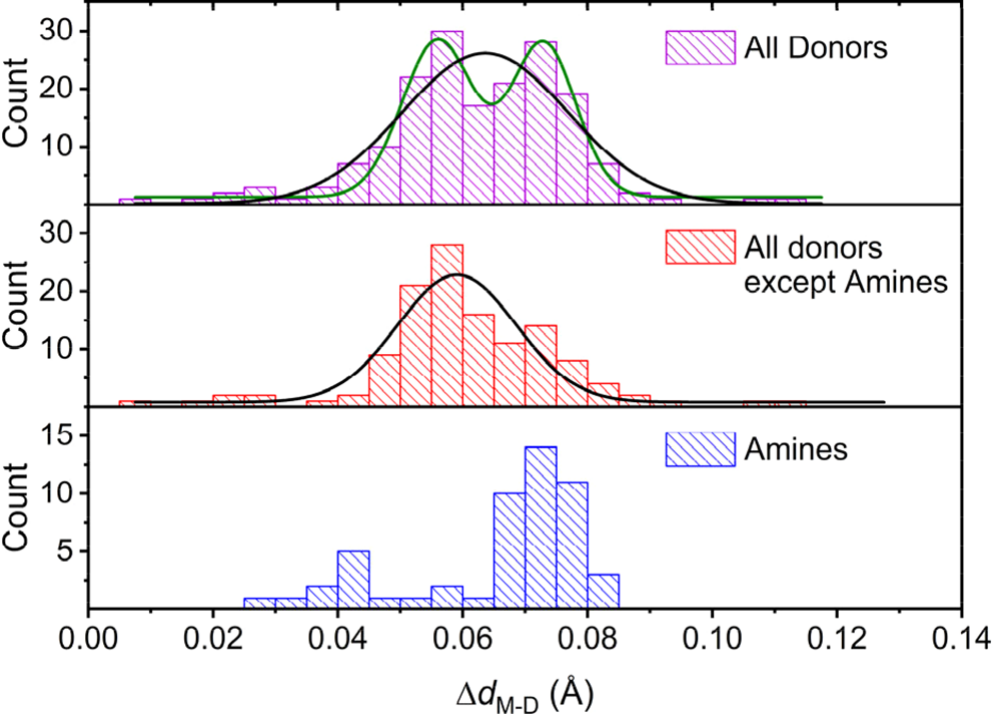
Histograms showing the differences in bond distances calculated for equivalent donors in La^3+^ and Ac^3+^ complexes. The distribution including all donors was fitted to a double Gaussian function and the distances excluding amine N atoms to a single Gaussian distribution (see text).

The ionic radius of Ac^3+^ has been the subject of some debate. Early studies on the crystal structure of AcCl_3_ provided an estimate of 1.153 Å for CN = 6,^56^ a value that was later used to estimate the ionic radius for CN = 9 (1.153 + 0.11 = 1.263 Å).^8^ More recently, Persson estimated an ionic radius for nine-coordinated Ac^3+^ of 1.220 Å by extrapolation of sizes of other An^3+^ ions, the latter value being also that recommended by Deblonde.^8^ However, the systematically positive Δ*d*_M‑D_ values obtained here point to the ionic radius of Ac^3+^ being significantly larger than that of La^3+^. Moreover, our results are consistent with the distances obtained with EXAFS for the La^3+^ (2.59 Å)^57^ and Ac^3+^ (2.66 Å)^11^ aqua-ions, which differ by 0.07 Å, a value that is very close to our estimate of Δ*d*_M‑D_.

The concept of ionic radius is extraordinary useful to rationalize coordination chemistry. It relies on the assumption that the distance between the metal ion and a donor atom can be broken down into their respective contributions. Indeed, Shannon derived ionic radii assuming that interatomic distances can be reproduced by adding both cation and anion radii. This approximation is expected to be valid for a given coordination number (CN) and electronic spin in systems with similar covalency, repulsive forces, and polyhedral distortion.^19^ Thus, we wondered whether the positive Δ*d*_M‑D_ values calculated are related to a higher contribution of Ac^3+^ to the Ac-donor bonds compared with La^3+^. In such case, the minimum of the electron density (ρ) along the Ac^3+^-donor paths is expected to be shifted further away from the position of the nucleus of the metal ion than in the corresponding La^3+^ complexes. This minimum corresponds to a (3,–1) critical point (CP) according to the terminology proposed by Bader, as it has three curvatures, one positive and two negative.^58,59^ This is indeed the case for the complexes investigated here, as illustrated in Figure 3 for the three types of donor atoms in [Ac­(NO3PA)] and [La­(NO3PA)]^+^. The electron density shows two maxima corresponding to the nuclear positions and displays a minimum along the bond path that corresponds to the (3,–1) CPs. The maxima corresponding to the position of the donor nuclei is systematically shifted further away from the metal ion in the Ac^3+^ complex compared to the La^3+^ one, as the M-donor bonds are shorter for La^3+^ than Ac^3+^. Furthermore, the position of the CPs also shifts further away from the metal ion in Ac^3+^ than in La^3+^, in line with the hypothesis that Ac^3+^ has a larger ionic radius than La^3+^. The separation between the CPs amounts to 0.044, 0.062, and 0.062 Å for carboxylate (O_C_), pyridine (N_PY_) and amine (N_AM_) donor atoms, respectively, which corresponds well to radii for CN = 9 of 1.275 Å for Ac^3+^ and 1.216 Å for La^3+^. The values of the electron density at the bond critical points (BCPs) observed for La^3+^ and Ac^3+^ are very similar.

**Figure 3:**
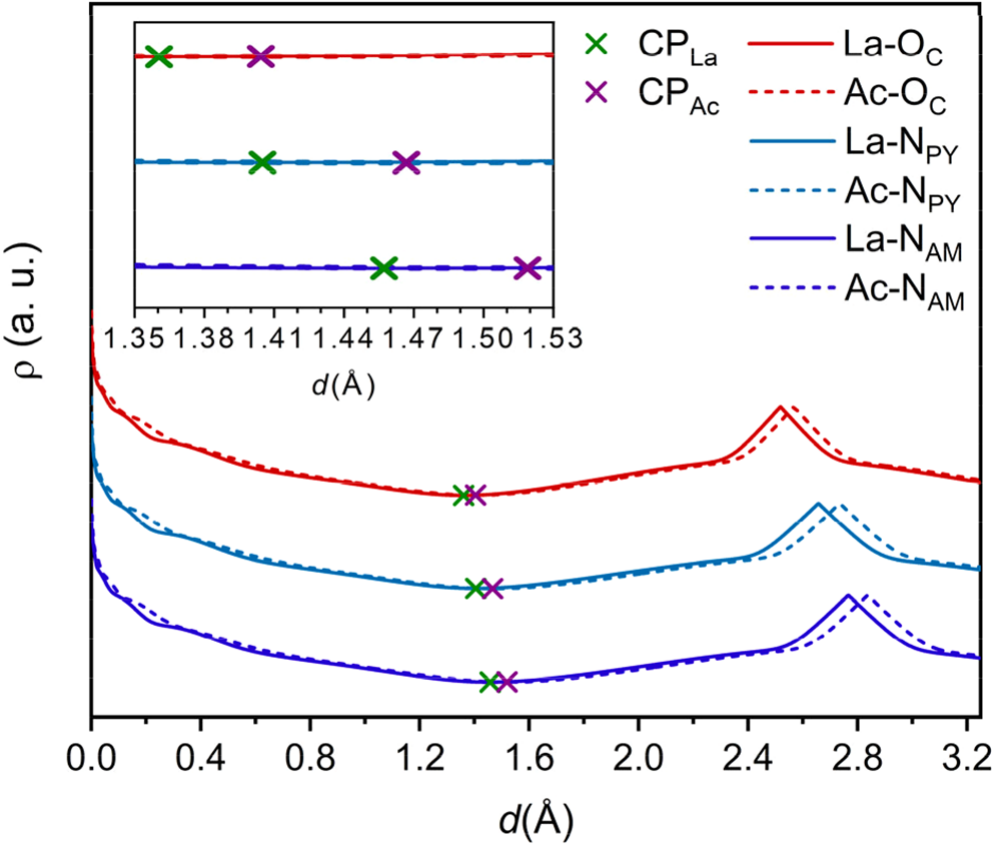
Electron densities along the paths following the metal-donor bonds in [Ac­(NO3PA)] and [La­(NO3PA)] complexes. The vertical scale is logarithmic and the data for carboxylate (O_C_) and pyridine (N_PY_) donors were shifted vertically for better visualization. The metal ion is placed at the origin and the (3,–1) critical points (CPs) are identified with crosses.

### Interaction Energies and Energy Decomposition Analysis (EDA)

Energy decomposition analysis is a very useful tool to gain information on the nature of the interaction between two molecular fragments. EDA has been used previously to rationalize chemical bonding and selectivity patterns for actinides over chemically similar lanthanides.^60^ Herein, we calculated the gas-phase interaction energies involving two different fragments, the ligand (L) and the metal ion (M^3+^ = La^3+^ or Ac^3+^), defined as^61^

1 $$E_{\mathrm{int}}=E\left(\mathrm{ML}\right)-\left[E\left(M\right)+E\left(L\right)\right]$$

The calculated *E*_int_ values studied here are static, as ligand deformation energies were not considered. Application of eq 1 with finite basis sets introduces basis-set superposition error (BSSE), which was estimated here using the counterpoise (CP) correction.^62,63^ The interaction energies calculated for the La^3+^ and Ac^3+^ complexes investigated here are given in Tables S18 and S19, Supporting Information. The gas-phase interaction energies become more negative with increasing the number of negatively charged donor groups of the ligand, as would be expected. However, this does not necessary translate into a higher complex stability. For instance, the DOTA^4–^ chelator forms more stable complexes with La^3+^ than DTPA^5–^. This is in part related to the increased hydration energies of the free ligands as their charge increases.

To gain insight into the factors that favor the complexation of Ac^3+^ over La^3+^, we decided to analyze the relative interaction energies (Δ*E*_int_) with a given ligand defined as Δ*E*_int_ = *E*_int,Ac_ – *E*_int,La_ (Table 1). The Δ*E*_int_ values obtained for the different complexes, including the aqua-ions, are all positive, with the only exception of the complex of decadentate TTHA^6–^ (Figure 4). This very likely reflects some degree of steric hindrance for the coordination of the ten donor atoms in the smaller La^3+^ ion compared with Ac^3+^. The X-ray structure of the La^3+^ complex supports this hypothesis, as it shows five La–O distances involving carboxylate groups in the range 2.47–2.56 Å and a significantly longer La–O distance of 2.71 Å.^47^

**Figure 4:**
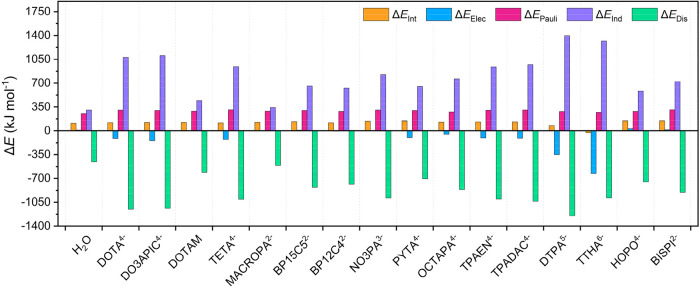
Relative values of interaction energies and of the EDA terms calculated as Δ*E* = *E*_Ac_ – *E*_La_ for La^3+^ and Ac^3+^ complexes.

**Table 1:** Relative Interaction Energies and Relative Values of the EDA Terms (kJ mol^–1^) for La^3+^ and Ac^3+^ Complexes^a^

ligand	Δ*E*_int_	Δ*E*_Elec_	Δ*E*_Pauli_	Δ*E*_Ind_	Δ*E*_Dis_	Δ*E*_Pol_
H_2_O^b^	114.0	9.7	252.4	305.4	–453.4	–148.0
DOTA^4–^	121.9	–112.0	303.9	1080.5	–1150.5	–70.0
DO3APIC^4–^	125.2	–143.9	298.9	1106.9	–1136.7	–29.8
DOTAM	125.5	3.3	288.3	446.0	–612.1	–166.1
TETA^4–^	119.1	–125.1	309.0	941.6	–1006.4	–64.8
MACROPA^2–^	127.5	6.4	288.1	341.6	–508.6	–167.0
BP15C5^2–^	136.4	9.3	298.8	657.2	–829.0	–171.8
BP12C4^2–^	120.7	–6.7	284.5	627.0	–784.1	–157.1
NO3PA^3–^	143.9	0.1	305.1	826.7	–988.0	–161.3
PYTA^4–^	146.0	–97.7	298.3	650.6	–705.3	–54.7
OCTAPA^4–^	129.7	–50.2	276.3	765.2	–861.7	–96.4
TPAEN^4–^	132.2	–104.0	302.1	937.5	–1003.4	–65.9
TPADAC^4–^	133.1	–108.4	306.3	970.5	–1035.2	–64.7
DTPA^5–^	79.2	–351.1	282.5	1397.5	–1249.7	147.8
TTHA^6–^	–28.3	–629.2	270.3	1317.6	–986.9	330.7
HOPO^4–^	148.5	30.9	284.7	583.4	–750.4	–167.0
BISPI^2–^	147.9	15.2	309.1	726.2	–902.6	–176.4

a Relative values were calculated as Δ*E* = *E*_Ac_ – *E*_La_, and thus negative values contribute to interaction energies more favorable for Ac^3+^ than La^3+^.

b Data obtained for the [M­(H_2_O)_9_]^3+^·21H_2_O systems (M = La or Ac).

The EDA scheme used in this work was developed by Mandado and Hermida-Ramón.^64^ It combines variational calculations with second-order perturbation theory, and can be regarded as an electron-density-based approach, since the decomposition follows from the partition of one-electron and exchange–correlation densities. For the two interacting systems, M^3+^ and L, the electron density, density matrix, and exchange–correlation density of the complex can be written as the sum of the corresponding isolated contributions associated with Pauli repulsion, polarization, and exchange. Substituting these expressions into the total energy expression and grouping terms with the same physical origin yields^64,65^

2 $$E_{\mathrm{Int}}=E_{\mathrm{Elec}}+E_{\mathrm{Pauli}}+E_{\mathrm{Pol}}$$

where the electrostatic term (*E*_Elec_) includes all interactions between unperturbed densities and nuclei of M^3+^ and L, *E*_Pauli_ (exchange + repulsion) arises from the antisymmetry requirement of the wave function, and *E*_Pol_ is the polarization contribution that accounts for charge induction, charge transfer, and dispersion.

Second-order perturbation theory provides rigorous, nonarbitrary expressions for induction and dispersion. Within this framework, the charge-induction energy corresponds to the interaction between the electrostatic potential of one fragment and the induced first-order density of the other. The induction energy is given by the intersystem component of this charge-induction term and equals half of the associated electrostatic interaction, consistent with the classical induction result. The difference between the polarization and induction energies contains the dispersion energy at second order, plus higher-order polarization–dispersion couplings when present. The final working expression for the interaction energy is therefore

3 $$E_{\mathrm{Int}}=E_{\mathrm{Elec}}+E_{\mathrm{Pauli}}+E_{\mathrm{Ind}}+E_{\mathrm{Disp}}$$

where *E*_Disp_ includes the dominant second-order dispersion contribution and the residual higher-order polarization terms. The results of the EDA are provided in Tables S18 and S19, Supporting Information. The results of the EDA evidence that the interaction energies are mostly dominated by the electrostatic contribution, which is the larger attractive contribution for all La^3+^ and Ac^3+^ complexes investigated, with the exception of those with the neutral DOTAM ligand. Overall, these results are in agreement with EDA performed on Ac^3+^ complexes with monodentate and bidentate ligands.^66^ Furthermore, for a given chelator the La^3+^ and Ac^3+^ complexes show a very similar weight of the *E*_Elec_ and *E*_Pol_ terms (Tables S18 and S19, Supporting Information).

The values of the relative terms (i.e., Δ*E*_Elec_ = *E*_Elec,Ac_ – *E*_Elec,La_) are given in Table 1.The relative electrostatic contributions (Δ*E*_elec_) take small positive or negative values for neutral ligands or ligands having 1-, 2- or 3- charges. However, the electrostatic term favors Ac^3+^ complexes for anionic ligands having 4-, 5- or 6- charge, with the only exception of HOPO^4–^. In the latter case, the delocalization of the negative charge on the 6-carboxylamide-1,2-hydroxypyridonate units is likely responsible for the positive Δ*E*_elec_ value.

The *E*_Pauli_ term is positive for both La^3+^ and Ac^3+^ complexes, and for a particular metal ion they take similar values in all complexes. The Pauli principle reduces the electron density in the region where interaction of overlapping filled orbitals takes place.^67,68^ As a result, more density is accumulated close to nuclei, resulting in a more negative potential energy and an increase of the kinetic energy, with the latter being responsible for the overall repulsive interaction.^69,70^ Since Ac^3+^ has more electrons than La^3+^, closed-shell electrons of the chelator overlap to a larger extent with Ac^3+^ than to La^3+^, resulting in positive Δ*E*_Pauli_ values for the former.

The polarization term Δ*E*_Pol_ = Δ*E*_Ind_ + Δ*E*_Dis_ is negative in most cases, as the negative Δ*E*_Dis_ term often compensates the positive Δ*E*_Ind_ contribution. The only exceptions to this general trend are observed for the ligands with the highest negative charges, DTPA^5–^ and TTHA^6–^ (Table 1). The positive Δ*E*_Ind_ values can be attributed to the higher charge density of the La^3+^ ion due to its smaller size compared with Ac^3+^. The frozen positive charge density of La^3+^ is therefore able to polarize more the density of the ligand than Ac^3+^. This effect is magnified as the negative charge of the ligand increases. The Δ*E*_Dis_ term provides the most negative contribution to the overall Δ*E*_Int_, thereby favoring Ac^3+^ over La^3+^ complexation. The *E*_Dis_ term is related to distortions of the charge density (charge transfer effects), and thus it is expected to favor Ac^3+^ over La^3+^, as covalent contributions are expected to be more important for the large Ac^3+^ ion than for the Ln^3+^ ions.^71,72^ The Δ*E*_Dis_ contribution becomes more negative on increasing the negative charge of the ligand, though this does not fully compensate the penalty arising from Δ*E*_Ind_ for ligands with a high negative charge such as DTPA^5–^ and TTHA^6–^.

Overall, EDA indicates that the Δ*E*_int_ values are the result of a delicate balance of different terms, with their relative role being not easy to predict in a chemically intuitive manner.

### Thermodynamic Analysis

The limitations of the polarized continuum models (PCM) of solvation to deal with ionic solutes that have concentrated charge densities make the calculation of stability constants rather cumbersome.^73^ This is the reason why stability constants are generally not directly compared with DFT-calculated values.^74^ Thus, the relative stability of La^3+^ and Ac^3+^ complexes was estimated by calculating the Gibbs free energies characterizing the following process

4 $$\left[\mathrm{LaL}\right]+{{\mathrm{Ac}}^{3+}}_{\mathrm{aq}}⇄\left[\mathrm{AcL}\right]+{{\mathrm{La}}^{3+}}_{\mathrm{aq}}$$

Here, *L* represents a given ligand and the charges of the complexes have been omitted for simplicity. We have used previously a similar approach to examine stability trends across the lanthanide series.^75^ In that work, we parametrized the radii of the Ln^3+^ ions so that the hydration free energies obtained with the PCM matched the experimental hydration energies. In this way, our calculations did not include explicit water molecules to model the Ln^3+^_aq_ species. However, application of the same approach here is problematic. In the case of the Ln^3+^ ions accurate free energies of hydration, consistent throughout the lanthanide series, are available.^76–78^ On the contrary, experimental data on Ac^3+^ hydration are scarce, with values differing by ∼90 kJ mol^–1^ having been reported. Thus, we used an alternative approach in which both Ac^3+^ and La^3+^ were modeled as clusters containing 30 explicit water molecules (Figure 5). The use of these models, accompanied by a PCM, is expected to provide an accurate description of the hydration of these metal ions. These clusters were based on those reported recently for Gd^3+^,^79^ which predicted correctly the relative stability of the [Gd­(H_2_O)_8_]^3+^ and [Gd­(H_2_O)_9_]^3+^ species.^80^ Similar clusters were also used recently for DFT studies and Born–Oppenheimer molecular dynamics simulations on the lanthanide and actinide aqua ions.^81,82^ The structure of the [M­(H_2_O)_9_]^3+^·21H_2_O clusters (M = La or Ac) afford tricapped trigonal prismatic coordination environments, as observed for the La^3+^ aqua-ion in the solid state.^13^ This is in contrast to classical molecular dynamics simulations, which favored a capped square antiprismatic coordination.^83^ Our calculated mean La–O and Ac–O distances of 2.595 and 2.663 Å, respectively, are in excellent agreement with the EXAFS values of 2.59 (La^3+^)^57^ and 2.66 Å (Ac^3+^)^11^ and the average La–O distance observed in X-ray structures (2.54 Å).^13^

**Figure 5:**
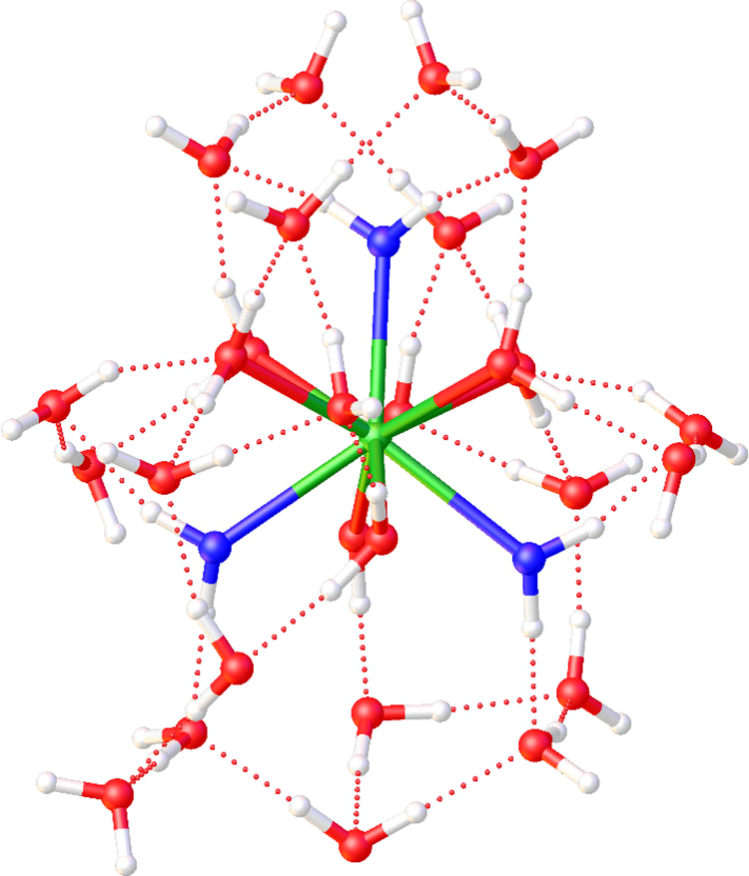
View of the structure of the [Ac­(H_2_O)_9_]^3+^·21H_2_O cluster obtained with DFT calculations. The view is perpendicular to the pseudo *C*_3_ axis of the tricapped trigonal prismatic coordination polyhedron, with apical water molecules drawn in blue.

The Gibbs free energies calculated for eq 4 are listed in Table 2. Interestingly, the Δ*G* ^298^ values are positive for all ligands investigated in this work, with the only exception of MACROPA^2–^, which is currently one of the leading chelator for Ac^3+^,^26,34,35^ and OCTAPA^4–^. The traditional chelators DOTA^4–^ and DTPA^5–^ are characterized by rather large positive Δ*G* ^298^ values, which evidence that they are not well suited for stable Ac^3+^ complexation. The TACN-based chelator NO3PA^3–^ is also characterized by a large positive Δ*G* ^298^ value, suggesting that the size of the TACN moiety is too small for the large Ac^3+^ ion. A similar situation holds for BISPI^2–^, in line with calculations of the ligand strain in bispidine derivatives, which showed that La^3+^ has a close to ideal size for the cavity of the ligand.^41^ The 18-membered macrocycle PYTA^4–^ also displays a large and positive Δ*G* ^298^ value; A recent study evidenced that the stability of PYTA^4–^ complexes with the Ln^3+^ ions increase at the beginning of the lanthanide series, reaches a maximum around Eu^3+^, and then decreases.^84^ Thus, the size of the 18-membered cavity of PYTA^4–^ appears to be ideal for medium-sized Ln^3+^ ions, and therefore somewhat small for Ac^3+^. The Δ*G* ^298^ values obtained for the structurally related BP12C4^2–^, BP15C5^2–^ and MACROPA^2–^ chelators evidenced the 18-membered cavity of the latter is best suited to achieve stable Ac^3+^ complexation, with the 15- and 12-membered analogues affording positive Δ*G* ^298^ values. Enlarging the macrocyclic cavity of DOTA^4–^ to give TETA^4–^ results in a slight reduction of Δ*G* ^298^, which remains nevertheless rather unfavorable.

**Table 2:** Thermodynamic Data Calculated for the Ac^3+^ Complexes Investigated in This Work and Experimental Stability Constants for the La^3+^ Analogues^a^

ligand	Δ*G* ^298^ [kJ mol^–1^]^a^	Δlog *K*_Ac–La_ ^a^	log *K*_Ac_ ^d^	log *K*_La_ ^b^	pLa^c^	pAc^c^ ^,^ ^d^	*I*	refs
DOTA^4–^	+16.09	–2.82	20.04	22.86	17.4	14.6	0.1 M KCl	^89–91^
DO3APIC^4–^	+18.79	–3.29	17.88	21.17	16.6	13.3	0.1 M KCl	^92^
DOTAM	+8.43	–1.48	8.87	10.35	10.8	9.3	0.1 M NaNO_3_	^93^
TETA^4–^	+10.04	–1.76	9.84	11.60	7.0	6.1	0.1 M KCl	^94^
MACROPA^2–^	–8.03	+1.41	16.40	14.99	15.6	17.0	0.1 M KCl	^27^
BP15C5^2–^	+5.13	–0.90	11.6	12.52	12.4	11.5	0.1 M KCl	^95^
BP12C4^2–^	+11.35	–1.99	14.82	16.81	15.6	13.6	0.1 M KCl	^96^
NO3PA^3–^	+17.88	–3.13	^e^	^e^	^e^	^e^	0.15 M NaCl	
PYTA^4–^	+21.09	–3.69	21.09	24.78	22.4	18.7	0.1 M (NMe_4_)Cl	^84^
OCTAPA^4–^	–2.46	+0.43	20.35	19.92	19.7	20.1	0.15 M NaCl	^97^
TPAEN^4–^	+4.34	–0.76	18.40	19.16	19.15	18.8	0.15 M NaCl	^43^
TPADAC^4–^	+5.74	–1.01	18.54	19.55	17.1	16.1	0.15 M NaCl	^43^
DTPA^5–^	+17.00	–2.98	16.38	19.36	16.9	13.9	0.1 M NaClO_4_	^98^
TTHA^6–^	+6.72	–1.18	23.26	24.44	20.1	19.0	0.1 M KNO_3_	^99^
HOPO^4–^	+9.74	–1.71	14.69	16.40	17.3	15.6	0.1 M KCl	^100^
BISPI^2–^	+17.03	–2.98	20.74	23.72	20.8	17.9	0.1 M KCl	^40^

a Values obtained with DFT calculations.

b Experimental data reported in the literature for La^3+^ complexes.

c pM values (pLa or pAc) are defined as −log­[M]_free_ at pH 7.4 for [M] = 1 μM and [L] = 10 μM.

d Calculated with DFT using the experimental data reported for the La^3+^ analogues as reference.

e Not reported.

For some structurally related chelators, an increase in ligand denticity reduces the Δ*G* ^298^ values, which points to a certain tendency of Ac^3+^ to adopt higher coordination numbers compared with La^3+^. This effect can be clearly observed by comparing the Δ*G* ^298^ values of octadentate DTPA^5–^ and decadentate TTHA^6–^. However, nonadentate DO3APIC^4–^ and octadentate DOTA^4–^ display very similar Δ*G* ^298^ values, a situation that can be ascribed to the coordination of the carboxylate oxygen of the picolinate group at the sterically demanding capping position.^85^ The situation is even more striking when TPAEN^4–^ and OCTAPA^4–^ are compared, as the decadentate TPAEN^4–^ is characterized by a positive Δ*G* ^298^ value, while this value is negative for octadentate OCTAPA^4–^. The comparison of the Δ*G* ^298^ values obtained for DOTA^4–^ (16.09 kJ mol^–1^) and DOTAM (8.43 kJ mol^–1^) suggests that increasing the softness of the ligand donor atoms may contribute to enhance the stability of the Ac^3+^ complex with respect to the La^3+^ analogue. This is in line with the slightly higher hardness of La^3+^ (η = 15.4) compared with Ac^3+^ (η = 14.4).^20,86^

The Δ*G* ^298^ data reported in Table 2 can be used to calculate the differences in the log *K* values that characterize the stability of the Ac^3+^ and La^3+^ complexes, defined as Δlog *K*_Ac–La_ = log *K*_Ac_ – log *K*_La_

5 $$\Delta \log K_{Ac\text{−}La}=\log\left(e^{-\left({\Delta G}^{298}/\mathrm{RT}\right)}\right)$$

The stability constant of the Ac^3+^ complex can be subsequently calculated if that of the La^3+^ analogue is available. The stability constants estimated in this way for the Ac^3+^ complexes are obviously lower than those of the La^3+^ analogues with the only exception of MACROPA^2–^ and OCTAPA^4–^. In the case of MACROPA^2–^, the stability of the Ac^3+^ complex increases by ∼1.4 log *K* units, reaching log *K*_Ac_ = 16.4. For OCTAPA^4–^ the stability constant of the Ac^3+^ complex increases by 0.43 log *K* units with respect to La^3+^. For all other complexes investigated here the stabilities of the Ac^3+^ complexes are lower than those of the corresponding La^3+^ analogues.

The stability of the complexes in physiological conditions is considered to correlate better with the pM values defined at pH 7.4 than with the stability constants.^87^ Since the protonation constants of the ligands shown in Table 2 are known, as well as the stability and protonation constants of their complexes, one can calculate the pM values from the corresponding speciation diagrams. The pM values (pLa or pAc) were calculated using a metal ion concentration of 10^–6^ M and a ligand concentration of 10^–5^ M, following the suggestion of Raymond.^88^

The different basicities of the ligands shown in Figure 1 provoke significant alterations of the trends observed for pM and log *K* values. For instance, the highest log *K*_Ac_ is obtained for TTHA^6–^ (23.26) followed by PYTA^4–^ (21.09) and BISPI^2–^ (20.74). However, these ligands are characterized by pAc values lower than those of OCTAPA^4–^, which affords the highest pAc value among the ligands considered here. This is clearly the result of the lower basicity of OCTAPA^4–^ ∑_*i*=1_^5^ log *K*_H_*i*__ = 22.20, where log *K*_H_*i*__ are the stepwise ligand protonation constants)^97^ compared with TTHA^6–^ (33.1),^99^ PYTA^4–^ (31.1)^84^ and BISPI^2–^ (29.2).^40^

## Conclusions

In this work, we have presented a methodology that can be used to predict the thermodynamic stabilities of Ac^3+^ complexes, providing that equilibrium constants for the La^3+^ analogues are known. Even in the absence of thermodynamic data for La^3+^, DFT can estimate the relative stability of the Ac^3+^ and La^3+^ complexes (Δlog *K*_Ac–La_ values). This approach will be very useful to develop the coordination chemistry of Ac^3+^, as experimental determination of stability constants of complexes with this metal ion is extremely challenging. We acknowledge that a high thermodynamic stability is not the only condition required to develop metal complexes for radiopharmaceutical applications, as kinetic inertness with respect to complex dissociation is very likely a more important requisite.^101^ However, stability is still key to avoid the release of the metal ion, and thermodynamic data important to guide ligand design.

Another important result obtained in the present study concerns the ionic radius of Ac^3+^ and its comparison with that of La^3+^. Our computational studies allowed us to estimate an ionic radius of 1.275 ± 0.020 Å for Ac^3+^ at coordination number nine, a value that is significantly higher than those reported for La^3+^ (1.216 and 1.206 Å). Nevertheless, experimental studies comparing the structures of La^3+^ and Ac^3+^ complexes are still required to validate our results obtained from computations.

Our DFT calculations indicate that MACROPA^2–^ forms a more stable complex with Ac^3+^ than with La^3+^, which is not surprising considering the performance of this chelator for [^225^Ac]­Ac^3+^ complexation. This result also provides confidence on the methodology developed here. Our calculations also unexpectedly identified OCTAPA^4–^ as a potential candidate for [^225^Ac]­Ac^3+^ radiopharmaceuticals. This is somewhat surprising considering the results obtained for the decadentate analogue TPAEN^4–^, which forms a more stable complex with La^3+^ than with Ac^3+^. Thus, increasing the denticity of the ligand does not necessary translate to an increased stability of Ac^3+^ complexes with respect to the La^3+^ counterparts. Increasing the softness of the donor atoms does reduce the energy penalty to form the Ac^3+^ complex from the La^3+^ analogue (i.e., replacing carboxylates by amides), but the stabilities of the complexes with amides are several orders of magnitude less stable than those with carboxylates. Overall, this DFT study indicates that the use of ligands with decreased basicity may provide a useful strategy to develop stable Ac^3+^ complexes. In principle, our methodology can also be extended to other actinide ions or to seek for ligands selective for actinides over lanthanides

## Computational Details

Geometry optimizations and frequency calculations of the La^3+^ and Ac^3+^ complexes were carried out using the ORCA program system (version 6.1.0),^102–105^ which uses the SHARK integral package.^106^ In these calculations we used the hybrid meta-GGA functional TPSSh^107^ in combination with the Def2-TZVPP^108^ basis set for ligand atoms. Relativistic effects were considered with the use of effective core potentials (ECPs). For La we used the Def2-ECP, which incorporates 46 electrons in the core,^109^ in combination with the def2-TZVPP^108^ orbital basis set. For Ac, we chose the Def-ECP (60 electrons in the core)^110^ together with the Def-TZVP^111^ basis set. We used the resolution of identity and chain of spheres approximation (RIJCOSX)^112–117^ with auxiliary basis sets generated by the AutoAux tool of ORCA.^118^ Our calculations incorporated the atom-pairwise dispersion correction with the Becke-Johnson damping scheme (D3BJ).^119,120^ The inclusion of the dispersion correction has a significant effect on the calculated bond distances of the metal coordination environment, improving their agreement with the available experimental X-ray data. Bulk water solvent effects were considered with the continuum solvation model SMD developed by Truhlar.^121–123^

Energy decomposition analysis was carried out performing single point energy calculations with the TPSSh^107^ functional and the Gaussian 16 package (version C.01).^124^ In these calculations scalar relativistic effects were incorporated using the Douglas-Kroll-Hess Hamiltonian of second order (DKH2),^125,126^ using the all-electron SARC-DKH^127,128^ basis set for Ac and La and the decontracted Def2-TZVPP^108^ basis for all other atoms. The wave functions obtained from these calculations were subsequently analyzed with the EDA-NCI program.^129^ Electron densities and the positions of the critical points were calculated using the Multiwfn program (version 3.7).^130,131^

The pM values were calculated from the equilibrium constants using the HySS2009 program.^132^

## Supplementary Material



## Notes

Views of the calculated structures, bond distances of the metal coordination environments, electron density plots, EDA data and optimized Cartesian coordinates (PDF)
