# Supplementary material for: Stabilities of Ac3+ Complexes Relevant as Radiopharmaceuticals
Source: Inorg Chem. 2026 Feb 24;65(9):5119–30. doi: 10.1021/acs.inorgchem.5c05846 (PMC12977054; doi:10.1021/acs.inorgchem.5c05846)
Supplement: Supplementary file 1 [file ic5c05846_si_001.pdf]

Supporting Information for:

On the Stabilities of Ac^{3+} Complexes relevant as Radiopharmaceuticals

Antía Freire-García,[a,†] Raúl Alvarado,[a,†] María Costa-DeDios,[a] David Esteban-Gómez,[a] and Carlos Platas-Iglesias.*,[a]

[a] CICA - Centro Interdisciplinar de Química e Bioloxía and Departamento de Química, Facultade de Ciencias, Universidade da Coruña, 15071, A Coruña, Galicia, Spain.

*E-mail: carlos.platas.iglesias@udc.es

Summary:

Figure S1: DFT optimized structure of $[La(H_2O)_9]^{3+}\cdot 21H_2O$ showing the numeration of the donor atoms. 6

Table S1: Comparison of calculated metal-donor bond distances ($d_{M\text{-}D}$) for La^{3+} and Ac^{3+} aquo complexes....6

Figure S2: DFT optimized structure of $[La(DOTA)(H_2O)]^-$ showing the numeration of the donor atoms. Hydrogen atoms bonded to C atoms are omitted for simplicity....7

Table S2: Comparison of calculated metal-donor bond distances ($d_{M\text{-}D}$) for La^{3+} and Ac^{3+} complexes of DOTA, and the bond distances found in $[La(DOTA)(H_2O)]^-$ X-Ray structure. 7

Figure S3: DFT optimized structure of $[La(DO3APIC)]^-$ showing the numeration of the donor atoms. Hydrogen atoms are omitted for simplicity....8

Table S3: Comparison of calculated metal-donor bond distances ($d_{M\text{-}D}$) for La^{3+} and Ac^{3+} complexes of DO3APIC. 8

Figure S4: DFT optimized structure of $[La(DOTAM)(H_2O)]^{3+}$ showing the numeration of the donor atoms. Hydrogen atoms bonded to C atoms are omitted for simplicity....9

Table S4: Comparison of calculated metal-donor bond distances ($d_{M\text{-}D}$) for La^{3+} and Ac^{3+} complexes of DOTAM. 9

Figure S5: DFT optimized structure of $[La(TETA)(H_2O)]^-$ showing the numeration of the donor atoms. Hydrogen atoms bonded to C atoms are omitted for simplicity....10

Table S5: Table S5: Comparison of calculated metal-donor bond distances ($d_{M\text{-}D}$) for La^{3+} and Ac^{3+} complexes of TETA. 10

Figure S6: DFT optimized structure of $[La(MACROPA)(H_2O)]^+$ showing the numeration of the donor atoms. Hydrogen atoms bonded to C atoms are omitted for simplicity. 11

Table S6: Comparison of calculated metal-donor bond distances ($d_{M\text{-}D}$) for La^{3+} and Ac^{3+} complexes of MACROPA, and the bond distances found in $[La(MACROPA)]^+$ X-Ray structure....11

Figure S7: DFT optimized structure of $[La(BP15C5)]^+$ showing the numeration of the donor atoms. Hydrogen atoms are omitted for simplicity....12

Table S7: Comparison of calculated metal-donor bond distances ($d_{M\text{-}D}$) for La^{3+} and Ac^{3+} complexes of BP15C5....12

Figure S8: DFT optimized structure of $[La(BP12C4)(H_2O)_2]^+$ showing the numeration of the donor atoms. Hydrogen atoms bonded to C atoms are omitted for simplicity....13

Table S8: Comparison of calculated metal-donor bond distances ($d_{M\text{-}D}$) for La^{3+} and Ac^{3+} complexes of BP12C4....13

Figure S9: DFT optimized structure of [La(NO3PA)] showing the numeration of the donor atoms. Hydrogen atoms bonded to C atoms are omitted for simplicity....14

Table S9: Comparison of calculated metal-donor bond distances ($d_{M\text{-}D}$) for La^{3+} and Ac^{3+} complexes of NO3PA. 14

Figure S10: DFT optimized structure of $[La(PYTA)]^-$ showing the numeration of the donor atoms. Hydrogen atoms are omitted for simplicity....15

Table S10: Comparison of calculated metal-donor bond distances (d_{M-D}) for La^{3+} and Ac^{3+} complexes of PYTA, and the bond distances found in $[La(PYTA)]^{-}$ X-Ray structure. 15

Figure S11: DFT optimized structure of $[La(OCTAPA)(H_2O)_2]^{-}$ showing the numeration of the donor atoms. Hydrogen atoms bonded to C atoms are omitted for simplicity. 16

Table S11: Comparison of calculated metal-donor bond distances (d_{M-D}) for La^{3+} and Ac^{3+} complexes of OCTAPA, and the bond distances found in $[La(OCTAPA)]^{-}$ X-Ray structure. 16

Figure S12: DFT optimized structure of $[La(TPAEN)]^{-}$ showing the numeration of the donor atoms. Hydrogen atoms are omitted for simplicity. 17

Table S12: Comparison of calculated metal-donor bond distances (d_{M-D}) for La^{3+} and Ac^{3+} complexes of TPAEN, and the bond distances found in $[La(TPAEN)]^{-}$ X-Ray structure. 17

Figure S13: DFT optimized structure of $[La(TPADAC)]^{-}$ showing the numeration of the donor atoms. Hydrogen atoms are omitted for simplicity. 18

Table S13: Comparison of calculated metal-donor bond distances (d_{M-D}) for La^{3+} and Ac^{3+} complexes of TPADAC, and the bond distances found in $[La(TPADAC)]^{-}$ X-Ray structure. 18

Figure S14: DFT optimized structure of $[La(DTPA)(H_2O)]^{2-}$ showing the numeration of the donor atoms. Hydrogen atoms bonded to C atoms are omitted for simplicity......19

Table S14: Comparison of calculated metal-donor bond distances (d_{M-D}) for La^{3+} and Ac^{3+} complexes of DTPA, and the bond distances found in $[La(DTPA)]^{2-}$ X-Ray structure. 19

Figure S15: DFT optimized structure of $[La(TTHA)]^{3-}$ showing the numeration of the donor atoms. Hydrogen atoms are omitted for simplicity. 20

Table S15: Comparison of calculated metal-donor bond distances (d_{M-D}) for La^{3+} and Ac^{3+} complexes of TTHA, and the bond distances found in $[La(TTHA)]^{3-}$ X-Ray structure. 20

Figure S16: DFT optimized structure of $[La(HOPO)(H_2O)]^{3+}$ showing the numeration of the donor atoms. Hydrogen atoms bonded to C atoms are omitted for simplicity......21

Table S16: Comparison of calculated metal-donor bond distances (d_{M-D}) for La^{3+} and Ac^{3+} complexes of HOPO. 21

Figure S17: DFT optimized structure of $[La(BISPI)]^{+}$ showing the numeration of the donor atoms. Hydrogen atoms are omitted for simplicity. 22

Table S17: Comparison of calculated metal-donor bond distances (d_{M-D}) for La^{3+} and Ac^{3+} complexes of BISPI. 22

Figure S18: Electron densities along the paths following the metal-donor bonds in $[Ac(TPAEN)]^{-}$ and $[La(TPAEN)]^{-}$ complexes. The vertical scale is logarithmic and the data for carboxylate (O_C) and pyridine (N_{PY}) donors were shifted vertically for better visualization. The metal ion is placed at the origin and the (3,-1) critical points (CPs) are identified with crosses. 23

Figure S19: Electron densities along the paths following the metal-donor bonds in $[Ac(MACROPA)]^+$ and $[La(MACROPA)]^+$ complexes. The vertical scale is logarithmic and the data for carboxylate (O_C) and pyridine (N_{PY}) donors were shifted vertically for better visualization. The metal ion is placed at the origin and the (3,-1) critical points (CPs) are identified with crosses. 23

Table S18: Interaction energies and values of the EDA terms calculated for the La^{3+} complexes in $kJ \cdot mol^{-1}$ 24

Table S19: Interaction energies and values of the EDA terms calculated for the Ac^{3+} complexes in $kJ \cdot mol^{-1}$ 25

Table S20: Cartesian coordinates (Å) obtained from geometry optimizations for $[La(H_2O)_9]^{3+} \cdot 21H_2O$. 26

Table S21: Cartesian coordinates (Å) obtained from geometry optimizations for $[Ac(H_2O)_9]^{3+} \cdot 21H_2O$. 28

Table S22: Cartesian coordinates (Å) obtained from geometry optimizations for $[La(DOTA)(H_2O)]^-$. 30

Table S23: Cartesian coordinates (Å) obtained from geometry optimizations for $[Ac(DOTA)(H_2O)]^-$. 31

Table S24: Cartesian coordinates (Å) obtained from geometry optimizations for $[La(DO3APIC)]^-$. 32

Table S25: Cartesian coordinates (Å) obtained from geometry optimizations for $[Ac(DO3APIC)]^-$. 33

Table S26: Cartesian coordinates (Å) obtained from geometry optimizations for $[La(DOTAM)(H_2O)]^{3+}$. 35

Table S27: Cartesian coordinates (Å) obtained from geometry optimizations for $[Ac(DOTAM)(H_2O)]^{3+}$. 36

Table S28: Cartesian coordinates (Å) obtained from geometry optimizations for $[La(TETA)(H_2O)]^-$. 37

Table S29: Cartesian coordinates (Å) obtained from geometry optimizations for the $[Ac(TETA)(H_2O)]^-$. 39

Table S30: Cartesian coordinates (Å) obtained from geometry optimizations for $[La(MACROPA)(H_2O)]^+$. 40

Table S31: Cartesian coordinates (Å) obtained from geometry optimizations for $[Ac(MACROPA)(H_2O)]^+$. 42

Table S32: Cartesian coordinates (Å) obtained from geometry optimizations for $[La(BP15C5)]^+$. 43

Table S33: Cartesian coordinates (Å) obtained from geometry optimizations for $[Ac(BP15C5)]^+$. 45

Table S34: Cartesian coordinates (Å) obtained from geometry optimizations for $[La(BP12C4)(H_2O)_2]^+$. 46

Table S35: Cartesian coordinates (Å) obtained from geometry optimizations for $[Ac(BP12C4)(H_2O)_2]^+$. 48

Table S36: Cartesian coordinates (Å) obtained from geometry optimizations for [La(NO3PA)]....49

Table S37: : Cartesian coordinates (Å) obtained from geometry optimizations for [Ac(NO3PA)]....50

Table S38: Cartesian coordinates (Å) obtained from geometry optimizations for $[La(PYTA)]^-$....52

Table S39: Cartesian coordinates (Å) obtained from geometry optimizations for $[Ac(PYTA)]^-$....53

Table S40: Cartesian coordinates (Å) obtained from geometry optimizations for $[La(OCTAPA)(H_2O)_2]^-$....55

Table S41: Cartesian coordinates (Å) obtained from geometry optimizations for $[Ac(OCTAPA)(H_2O)_2]^-$....56

Table S42: Cartesian coordinates (Å) obtained from geometry optimizations for $[La(TPAEN)]^-$....57

Table S43: Cartesian coordinates (Å) obtained from geometry optimizations for $[Ac(TPAEN)]^-$....59

Table S44: Cartesian coordinates (Å) obtained from geometry optimizations for $[La(TPADAC)]^-$....60

Table S45: Cartesian coordinates (Å) obtained from geometry optimizations for $[Ac(TPADAC)]^-$....62

Table S46: Cartesian coordinates (Å) obtained from geometry optimizations for $[La(DTPA)(H_2O)]^{2-}$....64

Table S47: Cartesian coordinates (Å) obtained from geometry optimizations for $[Ac(DTPA)(H_2O)]^{2-}$....65

Table S48: Cartesian coordinates (Å) obtained from geometry optimizations for $[La(TTHA)]^{3-}$....66

Table S49: Cartesian coordinates (Å) obtained from geometry optimizations for $[Ac(TTHA)]^{3-}$....67

Table S50: Cartesian coordinates (Å) obtained from geometry optimizations for $[La(HOPO)(H_2O)]^-$....68

Table S51: Cartesian coordinates (Å) obtained from geometry optimizations for $[Ac(HOPO)(H_2O)]^-$....70

Table S52: Cartesian coordinates (Å) obtained from geometry optimizations for $[La(BISPI)]^+$....72

Table S53: Cartesian coordinates (Å) obtained from geometry optimizations for $[Ac(BISPI)]^+$....74

References 76

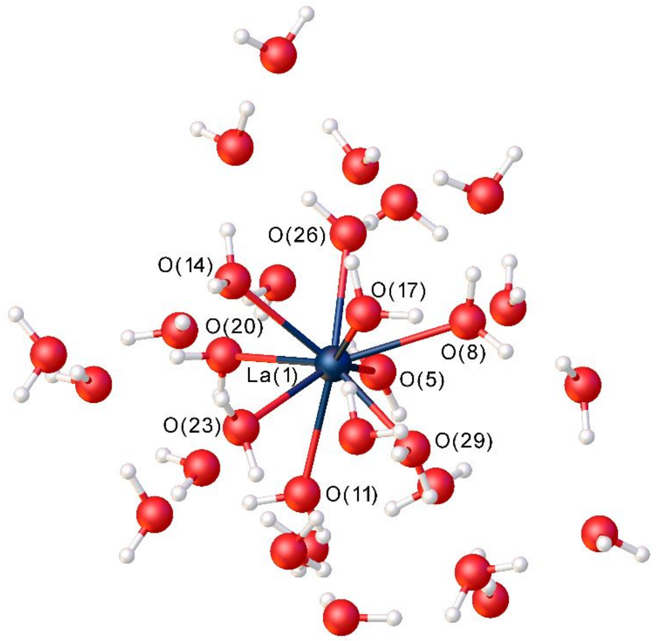

Figure S1: DFT optimized structure of $[La(H_2O)_9]^{3+}\cdot 21H_2O$ showing the numeration of the donor atoms.

Table S1: Comparison of calculated metal-donor bond distances ($d_{M\text{-}D}$) for La^{3+} and Ac^{3+} aquo complexes.

**Donor type**		$d_{M\text{-}D}$ **(Å)**	
		DFT La	DFT Ac
**aquo**	M-O5	2.573	2.635
	M-O8	2.624	2.692
	M-O11	2.598	2.661
	M-O14	2.568	2.627
	M-O17	2.640	2.717
	M-O20	2.636	2.708
	M-O23	2.579	2.653
	M-O26	2.573	2.645
	M-O29	2.565	2.627

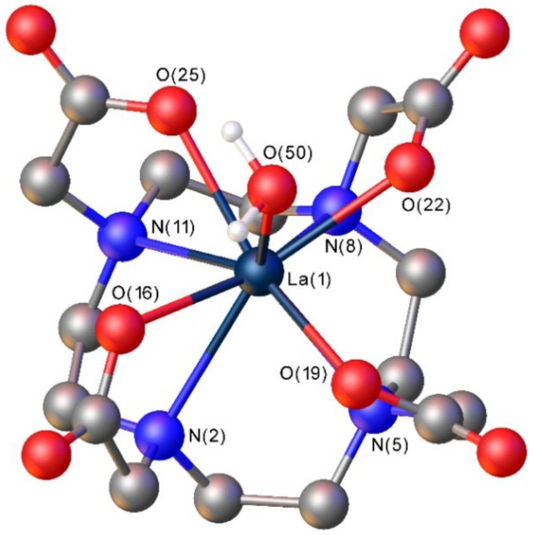

Figure S2: DFT optimized structure of $[La(DOTA)(H_2O)]^-$ showing the numeration of the donor atoms. Hydrogen atoms bonded to C atoms are omitted for simplicity.

Table S2: Comparison of calculated metal-donor bond distances ($d_{M\text{-}D}$) for La^{3+} and Ac^{3+} complexes of DOTA, and the bond distances found in $[La(DOTA)(H_2O)]^-$ X-Ray structure.

**Donor type**		$d_{M\text{-}D}$ (Å)		
		DFT La	DFT Ac	X-Ray[1]
**amine**	M-N2	2.752	2.823	2.788
	M-N5	2.750	2.823	2.781
	M-N8	2.755	2.827	2.759
	M-N11	2.744	2.818	2.751
**carboxylate**	M-O16	2.518	2.571	2.490
	M-O19	2.507	2.560	2.509
	M-O22	2.510	2.562	2.483
	M-O25	2.510	2.563	2.486
**aquo**	M-O50	2.686	2.772	2.538

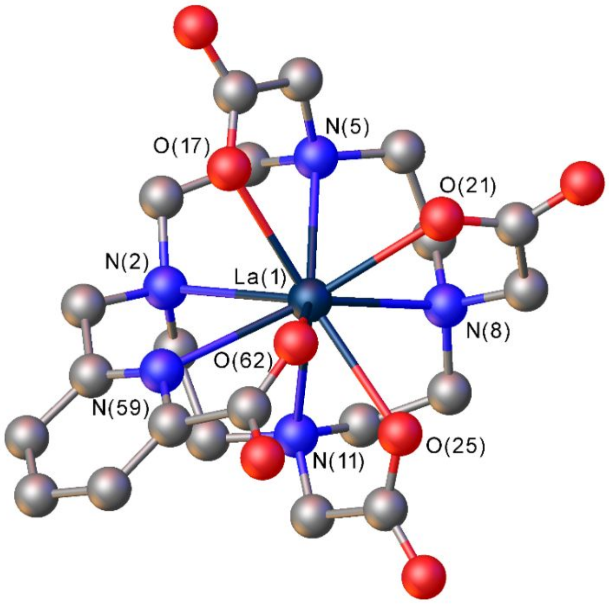

Figure S3: DFT optimized structure of $[La(DO3APIC)]^-$ showing the numeration of the donor atoms. Hydrogen atoms are omitted for simplicity.

Table S3: Comparison of calculated metal-donor bond distances ($d_{M\text{-}D}$) for La^{3+} and Ac^{3+} complexes of DO3APIC.

**Donor type**		**$d_{M\text{-}D}$ (Å)**	
		DFT La	DFT Ac
**amine**	M-N2	2.807	2.878
	M-N5	2.760	2.843
	M-N8	2.756	2.836
	M-N11	2.757	2.828
**pyridine**	M-O59	2.663	2.746
**carboxylate**	M-O17	2.496	2.548
	M-O21	2.472	2.531
	M-O25	2.524	2.577
	M-O62	2.541	2.594

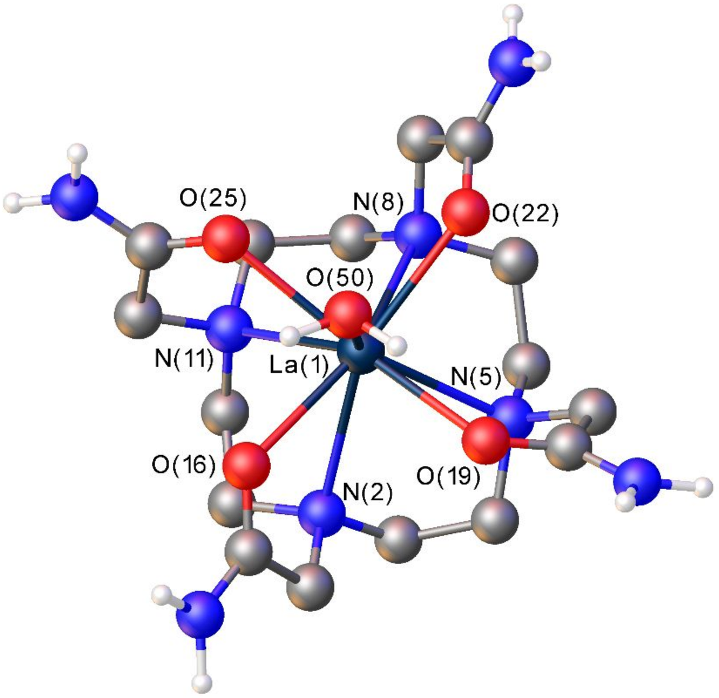

Figure S4: DFT optimized structure of $[La(DOTAM)(H_2O)]^{3+}$ showing the numeration of the donor atoms. Hydrogen atoms bonded to C atoms are omitted for simplicity.

Table S4: Comparison of calculated metal-donor bond distances ($d_{M\text{-}D}$) for La^{3+} and Ac^{3+} complexes of DOTAM.

**Donor type**		$d_{M\text{-}D}$ **(Å)**	
		DFT La	DFT Ac
**amine**	M-N2	2.756	2.829
	M-N5	2.744	2.809
	M-N8	2.754	2.830
	M-N11	2.751	2.826
**amide**	M-O16	2.542	2.597
	M-O19	2.539	2.596
	M-O22	2.534	2.588
	M-O25	2.530	2.589
**aquo**	M-O50	2.627	2.695

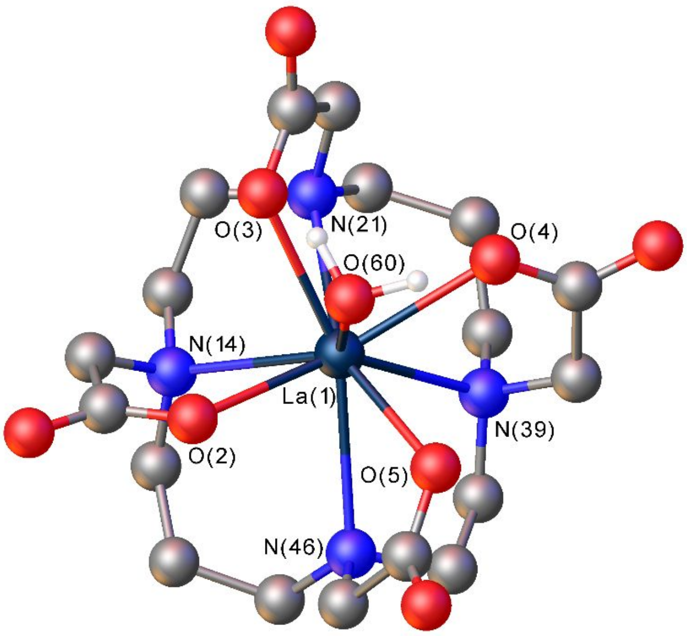

Figure S5: DFT optimized structure of $[La(TETA)(H_2O)]^-$ showing the numeration of the donor atoms. Hydrogen atoms bonded to C atoms are omitted for simplicity.

Table S5: Table S5: Comparison of calculated metal-donor bond distances ($d_{M\text{-}D}$) for La^{3+} and Ac^{3+} complexes of TETA.

**Donor type**		**$d_{M\text{-}D}$ (Å)**	
		DFT La	DFT Ac
**amine**	M-N2	2.797	2.863
	M-N5	2.766	2.831
	M-N8	2.770	2.840
	M-N11	2.772	2.839
**carboxylate**	M-O59	2.681	2.759
	M-O17	2.516	2.565
	M-O21	2.489	2.545
	M-O25	2.534	2.589
**aquo**	M-O62	2.537	2.589

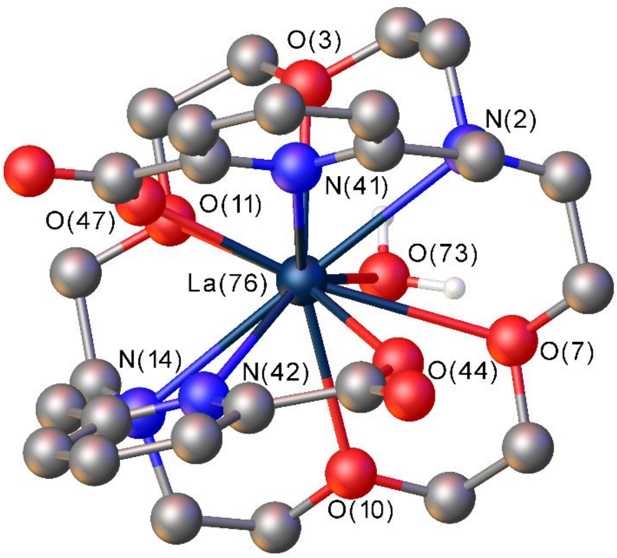

Figure S6: DFT optimized structure of $[La(MACROPA)(H_2O)]^+$ showing the numeration of the donor atoms. Hydrogen atoms bonded to C atoms are omitted for simplicity.

Table S6: Comparison of calculated metal-donor bond distances ($d_{M\text{-}D}$) for La^{3+} and Ac^{3+} complexes of MACROPA, and the bond distances found in $[La(MACROPA)]^+$ X-Ray structure.

**Donor type**		$d_{M\text{-}D}$ (Å)		
		DFT La	DFT Ac	X-Ray[2]
**amine**	M-N2	2.908	2.934	2.941
	M-N14	2.882	2.920	2.898
**pyridine**	M-N41	2.699	2.768	2.759
	M-N42	2.701	2.767	2.776
**carboxylate**	M-O44	2.503	2.568	2.552
	M-O47	2.489	2.564	2.527
**ether**	M-O3	2.755	2.791	2.700
	M-O7	2.949	2.882	2.791
	M-O10	2.776	2.801	2.731
	M-O11	2.837	2.843	2.794
**aquo**	M-O73	2.655	2.730	2.558

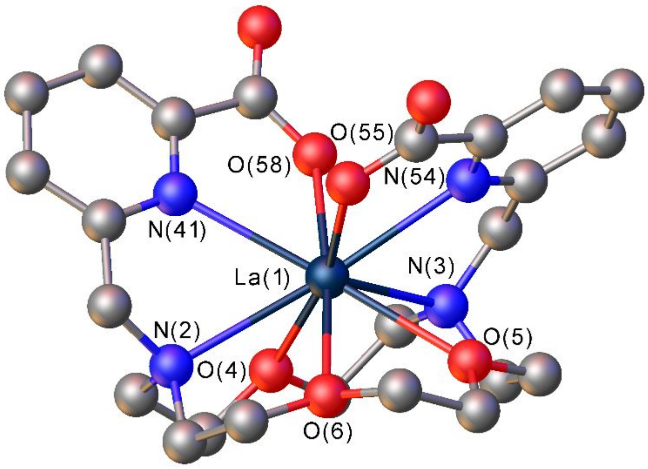

Figure S7: DFT optimized structure of $[La(BP15C5)]^+$ showing the numeration of the donor atoms. Hydrogen atoms are omitted for simplicity.

Table S7: Comparison of calculated metal-donor bond distances ($d_{M\text{-}D}$) for La^{3+} and Ac^{3+} complexes of BP15C5.

**Donor type**		**$d_{M\text{-}D}$ (Å)**	
		DFT La	DFT Ac
**amine**	M-N2	2.816	2.883
	M-N7	2.718	2.794
**pyridine**	M-N51	2.681	2.757
	M-N52	2.638	2.725
**carboxylate**	M-O54	2.446	2.502
	M-O58	2.484	2.530
**ether**	M-O33	2.579	2.650
	M-O34	2.660	2.715
	M-O14	2.642	2.693

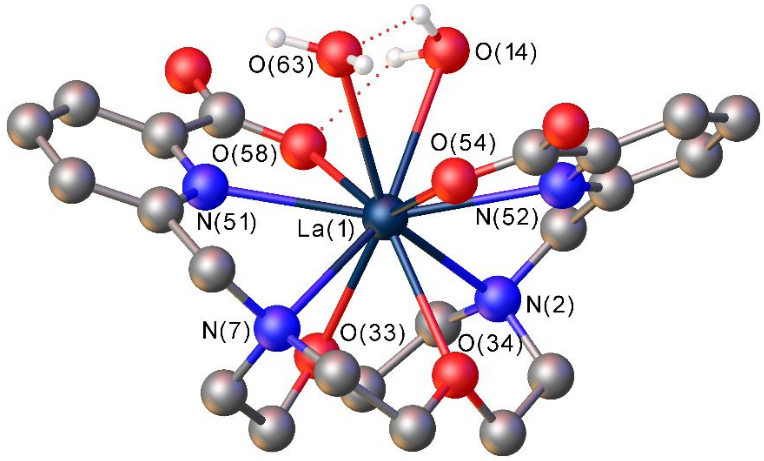

Figure S8: DFT optimized structure of $[La(BP12C4)(H_2O)_2]^+$ showing the numeration of the donor atoms. Hydrogen atoms bonded to C atoms are omitted for simplicity.

Table S8: Comparison of calculated metal-donor bond distances ($d_{M\text{-}D}$) for La^{3+} and Ac^{3+} complexes of BP12C4.

**Donor type**		***d*M-D (Å)**	
		DFT La	DFT Ac
**amine**	M-N2	2.700	2.778
	M-N7	2.714	2.783
**pyridine**	M-N51	2.681	2.750
	M-N52	2.644	2.726
**carboxylate**	M-O54	2.519	2.575
	M-O58	2.550	2.611
**ether**	M-O33	2.620	2.706
	M-O34	2.656	2.731
**aquo**	M-O14	2.846	2.816
	M-O63	2.747	2.773

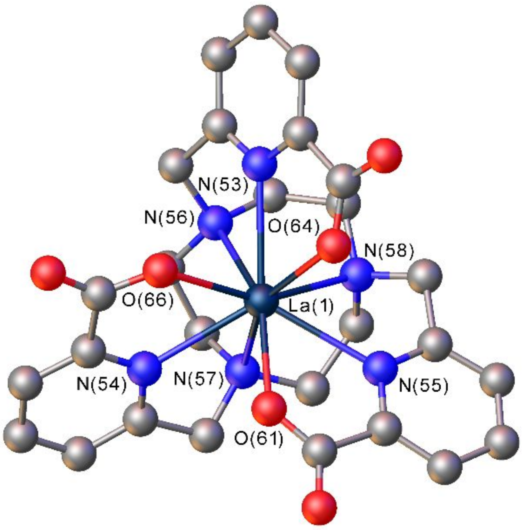

Figure S9: DFT optimized structure of [La(NO3PA)] showing the numeration of the donor atoms. Hydrogen atoms bonded to C atoms are omitted for simplicity.

Table S9: Comparison of calculated metal-donor bond distances (d_{M-D}) for La^{3+} and Ac^{3+} complexes of NO3PA**.**

**Donor type**		d_{M-D} **(Å)**	
		DFT La	DFT Ac
**amine**	M-N56	2.767	2.836
	M-N57	2.765	2.841
	M-N58	2.766	2.843
**pyridine**	M-N53	2.657	2.735
	M-N54	2.651	2.734
	M-N55	2.656	2.734
**carboxylate**	M-O61	2.519	2.567
	M-O64	2.518	2.567
	M-O66	2.520	2.563

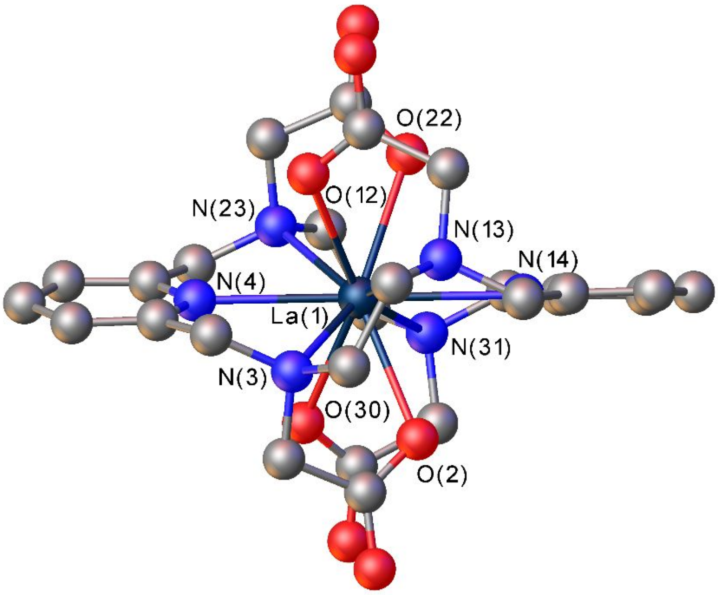

Figure S10: DFT optimized structure of $[La(PYTA)]^-$ showing the numeration of the donor atoms. Hydrogen atoms are omitted for simplicity.

Table S10: Comparison of calculated metal-donor bond distances ($d_{M\text{-}D}$) for La^{3+} and Ac^{3+} complexes of PYTA, and the bond distances found in $[La(PYTA)]^-$ X-Ray structure.

Donor type		$d_{M\text{-}D}$ (Å)		
		DFT La	DFT Ac	X-Ray[3]
**amine**	M-N3	2.720	2.763	2.701
	M-N13	2.720	2.763	2.698
	M-N23	2.720	2.763	2.692
	M-N31	2.720	2.763	2.718
**pyridine**	M-N4	2.656	2.684	2.636
	M-N14	2.656	2.684	2.637
**carboxylate**	M-O2	2.602	2.662	2.572
	M-O12	2.602	2.662	2.571
	M-O22	2.602	2.662	2.55
	M-O30	2.602	2.662	2.723

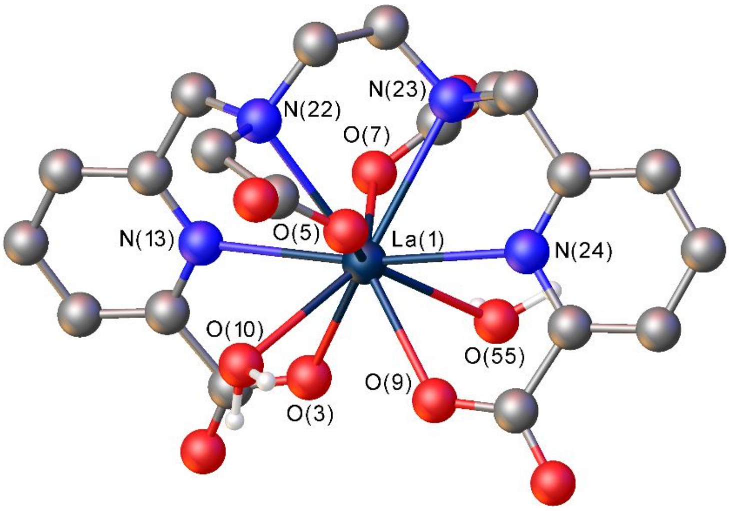

Figure S11: DFT optimized structure of $[La(OCTAPA)(H_2O)_2]^-$ showing the numeration of the donor atoms. Hydrogen atoms bonded to C atoms are omitted for simplicity.

Table S11: Comparison of calculated metal-donor bond distances (d_{M-D}) for La^{3+} and Ac^{3+} complexes of OCTAPA, and the bond distances found in $[La(OCTAPA)]^-$ X-Ray structure.

**Donor type**		d_{M-D} (Å)		
		DFT La	DFT Ac	X-Ray[4]
**amine**	M-N22	2.788	2.862	2.795
	M-N23	2.790	2.859	2.826
**pyridine**	M-N13	2.730	2.806	2.697
	M-N24	2.723	2.804	2.714
**carboxylate**	M-O3	2.534	2.596	2.527
	M-O5	2.541	2.598	2.583
	M-O7	2.549	2.599	2.548
	M-O9	2.539	2.595	2.544
**aquo**	M-O10	2.731	2.799	2.636
	M-O55	2.752	2.809	2.618

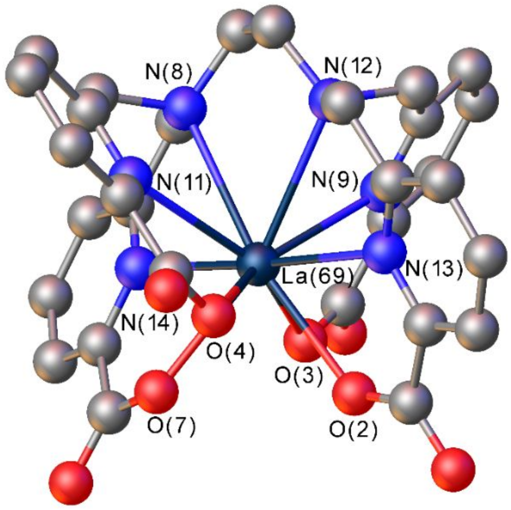

Figure S12: DFT optimized structure of $[La(TPAEN)]^-$ showing the numeration of the donor atoms. Hydrogen atoms are omitted for simplicity.

Table S12: Comparison of calculated metal-donor bond distances ($d_{M\text{-}D}$) for La^{3+} and Ac^{3+} complexes of TPAEN, and the bond distances found in $[La(TPAEN)]^-$ X-Ray structure.

**Donor type**		$d_{M\text{-}D}$ (Å)		
		DFT La	DFT Ac	X-Ray[5]
**amine**	M-N8	2.916	2.954	2.879
	M-N12	2.888	2.940	2.850
**pyridine**	M-N9	2.724	2.798	2.690
	M-N11	2.734	2.801	2.701
	M-N13	2.754	2.814	2.735
	M-N14	2.763	2.813	2.770
**carboxylate**	M-O2	2.542	2.594	2.537
	M-O3	2.553	2.609	2.498
	M-O4	2.539	2.600	2.617
	M-O7	2.536	2.591	2.547

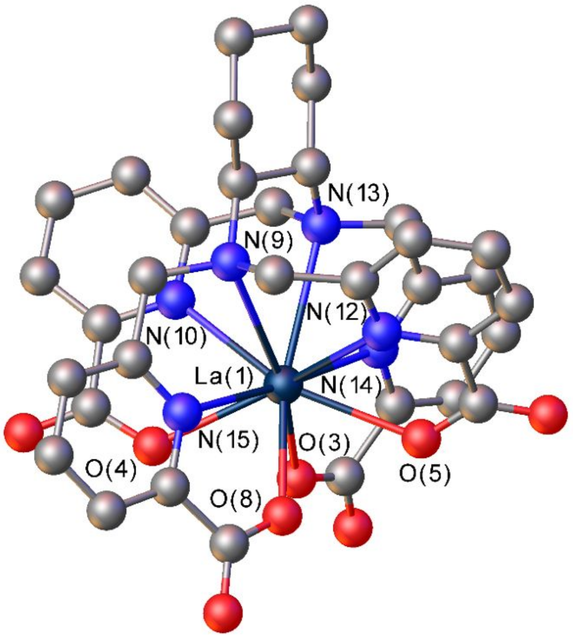

Figure S13: DFT optimized structure of $[La(TPADAC)]^-$ showing the numeration of the donor atoms. Hydrogen atoms are omitted for simplicity.

Table S13: Comparison of calculated metal-donor bond distances (d_{M-D}) for La^{3+} and Ac^{3+} complexes of TPADAC, and the bond distances found in $[La(TPADAC)]^-$ X-Ray structure.

**Donor type**		$\boldsymbol{d_{M-D}}$ **(Å)**		
		DFT La	DFT Ac	X-Ray[5]
**amine**	M-N9	2.941	2.975	2.969
	M-N13	2.920	2.962	2.931
**pyridine**	M-N10	2.712	2.777	2.691
	M-N12	2.721	2.783	2.708
	M-N14	2.740	2.796	2.723
	M-N15	2.746	2.798	2.699
**carboxylate**	M-O3	2.535	2.588	2.533
	M-O4	2.577	2.636	2.489
	M-O5	2.564	2.625	2.518
	M-O8	2.527	2.585	2.552

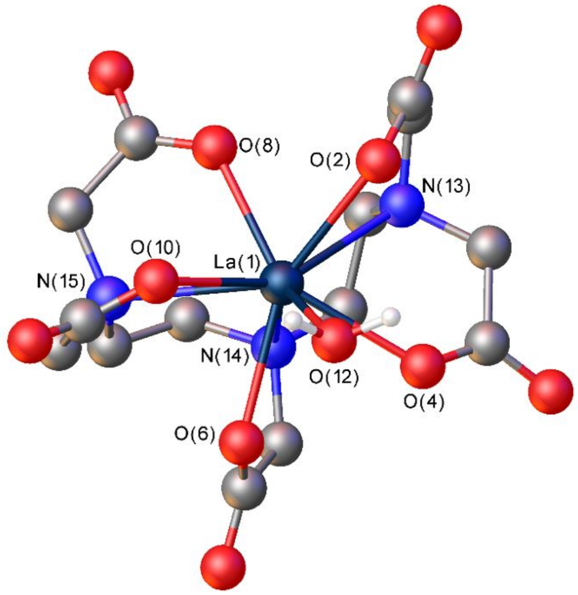

Figure S14: DFT optimized structure of $[La(DTPA)(H_2O)]^{2-}$ showing the numeration of the donor atoms. Hydrogen atoms bonded to C atoms are omitted for simplicity.

Table S14: Comparison of calculated metal-donor bond distances ($d_{M\text{-}D}$) for La^{3+} and Ac^{3+} complexes of DTPA, and the bond distances found in $[La(DTPA)]^{2-}$ X-Ray structure.

Donor type		$d_{M\text{-}D}$ (Å)		
		DFT La	DFT Ac	X-Ray[6]
**amine**	M-N13	2.759	2.829	2.842
	M-N14	2.737	2.821	2.832
	M-N15	2.792	2.868	2.772
**carboxylate**	M-O2	2.533	2.592	2.533
	M-O4	2.511	2.568	2.513
	M-O6	2.504	2.559	2.505
	M-O8	2.489	2.550	2.507
	M-O10	2.536	2.598	2.507
**aquo**	M-O12	2.666	2.745	2.478

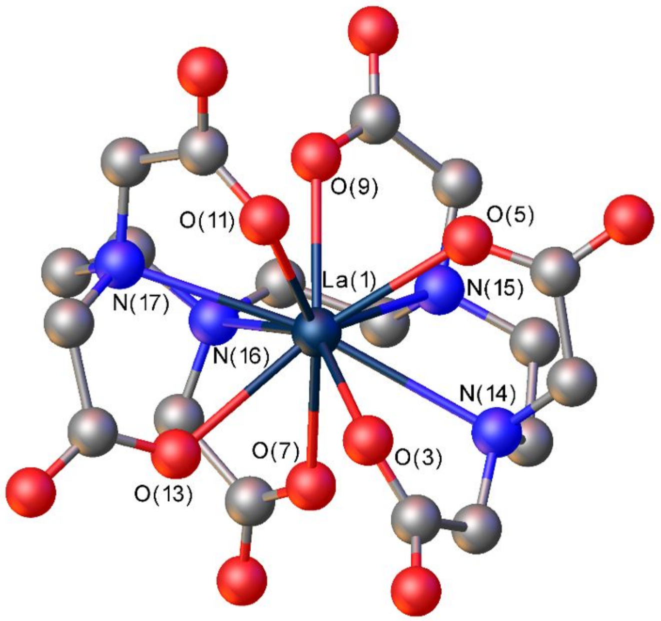

Figure S15: DFT optimized structure of $[La(TTHA)]^{3-}$ showing the numeration of the donor atoms. Hydrogen atoms are omitted for simplicity.

Table S15: Comparison of calculated metal-donor bond distances (d_{M-D}) for La^{3+} and Ac^{3+} complexes of TTHA, and the bond distances found in $[La(TTHA)]^{3-}$ X-Ray structure.

**Donor type**		$\boldsymbol{d_{M-D}}$ **(Å)**		
		DFT La	DFT Ac	X-Ray[7]
**amine**	M-N14	2.809	2.884	2.792
	M-N15	2.815	2.898	2.806
	M-N16	2.788	2.856	2.787
	M-N17	2.848	2.897	2.842
**carboxylate**	M-O3	2.584	2.631	2.546
	M-O5	2.560	2.613	2.555
	M-O7	2.559	2.619	2.470
	M-O9	2.534	2.607	2.502
	M-O11	2.582	2.634	2.560
	M-O13	2.616	2.668	2.711

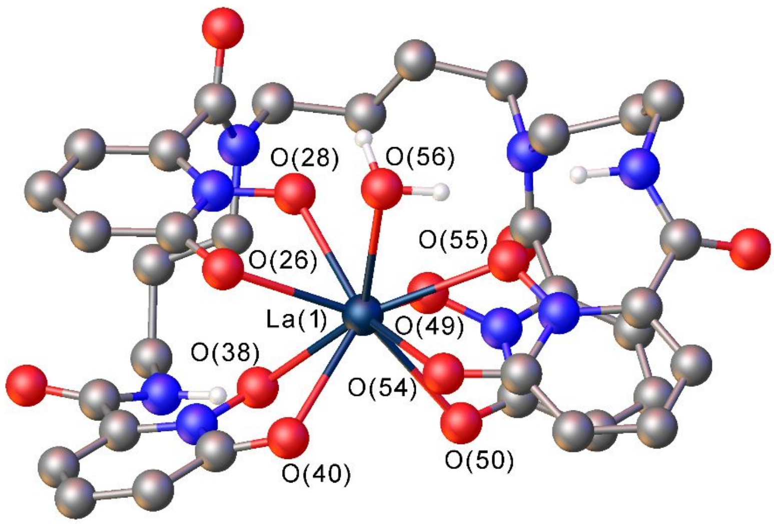

Figure S16: DFT optimized structure of $[La(HOPO)(H_2O)]^{3+}$ showing the numeration of the donor atoms. Hydrogen atoms bonded to C atoms are omitted for simplicity.

Table S16: Comparison of calculated metal-donor bond distances (d_{M-D}) for La^{3+} and Ac^{3+} complexes of HOPO.

**Donor type**		d_{M-D} (Å)	
		DFT La	DFT Ac
**hydroxy**	M-O28	2.546	2.602
	M-O38	2.554	2.601
	M-O49	2.536	2.608
	M-O55	2.594	2.650
**carbonyl**	M-O26	2.543	2.617
	M-O40	2.526	2.596
	M-O50	2.544	2.603
	M-O54	2.564	2.637
**aquo**	M-O56	2.726	2.811

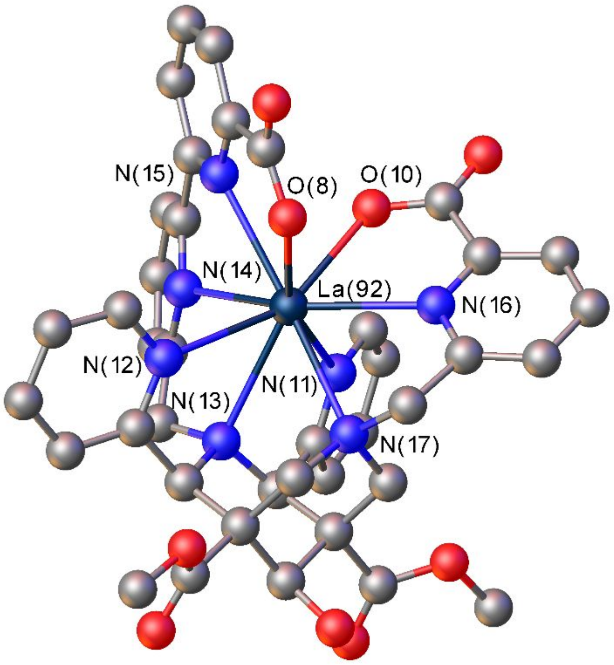

Figure S17: DFT optimized structure of $[La(BISPI)]^+$ showing the numeration of the donor atoms. Hydrogen atoms are omitted for simplicity.

Table S17: Comparison of calculated metal-donor bond distances ($d_{M\text{-}D}$) for La^{3+} and Ac^{3+} complexes of BISPI.

**Donor type**		**$d_{M\text{-}D}$ (Å)**	
		DFT La	DFT Ac
**amine**	M-N13	2.791	2.864
	M-N17	2.759	2.822
**pyridine**	M-N11	2.759	2.812
	M-N12	2.721	2.782
	M-N14	2.665	2.744
	M-N15	2.650	2.721
	M-N16	2.653	2.721
**carboxylate**	M-O8	2.476	2.530
	M-O10	2.508	2.558

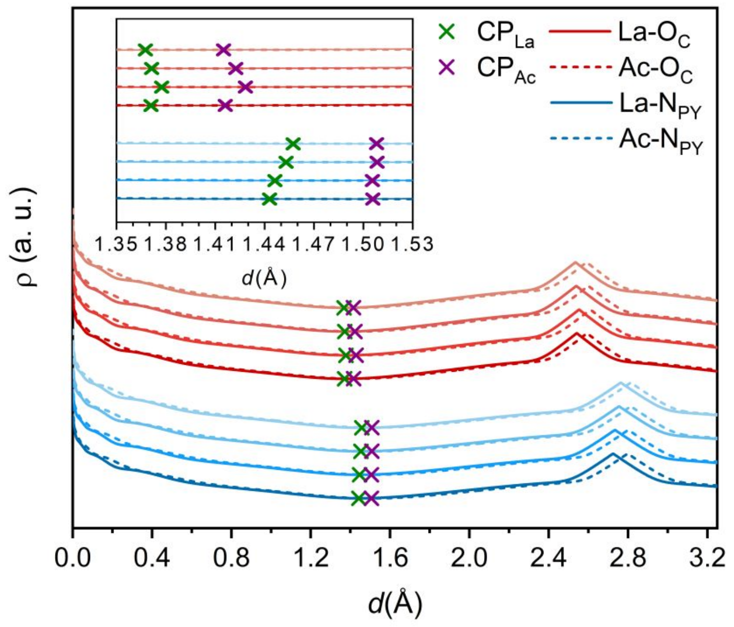

Figure S18: Electron densities along the paths following the metal-donor bonds in [Ac(TPAEN)]$^-$ and [La(TPAEN)]$^-$ complexes. The vertical scale is logarithmic and the data for carboxylate (O_C) and pyridine (N_{PY}) donors were shifted vertically for better visualization. The metal ion is placed at the origin and the (3,-1) critical points (CPs) are identified with crosses.

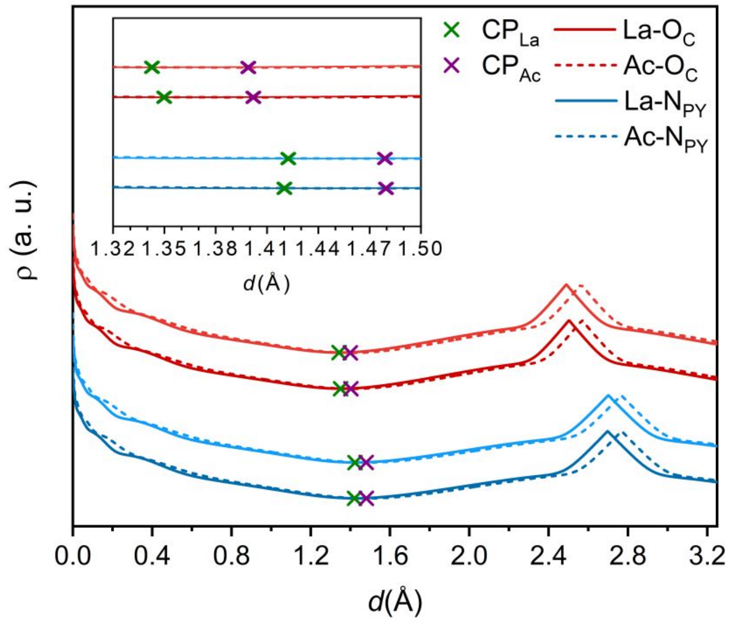

Figure S19: Electron densities along the paths following the metal-donor bonds in [Ac(MACROPA)]$^+$ and [La(MACROPA)]$^+$ complexes. The vertical scale is logarithmic and the data for carboxylate (O_C) and pyridine (N_{PY}) donors were shifted vertically for better visualization. The metal ion is placed at the origin and the (3,-1) critical points (CPs) are identified with crosses.

Table S18: Interaction energies and values of the EDA terms calculated for the La^{3+} complexes in kJ·mol^{-1}.

Ligand	E_{Int}	E_{Elec}	E_{Pauli}	E_{Ind}	E_{Dis}	E_{Pol}
**H_2O**	-3078.3	-2268.8	699.3	-531.3	-977.4	-1508.8
**$DOTA^{4-}$**	-5906.7	-4864.2	852.6	-259.1	-1635.9	-1895.0
**$DO3APIC^{4-}$**	-5907.5	-4775.9	874.1	-364.1	-1641.6	-2005.7
**DOTAM**	-2536.6	-1623.7	807.5	-449.4	-1271.1	-1720.5
**$TETA^{4-}$**	-5877.4	-4795.8	824.4	-204.8	-1701.1	-1905.9
**$MACROPA^{2-}$**	-4280.6	-3180.1	692.8	-170.2	-1623.1	-1793.3
**$BP15C5^{2-}$**	-4193.1	-3198.9	842.9	-279.7	-1557.5	-1837.2
**$BP12C4^{2-}$**	-4163.6	-3152.1	790.5	-251.5	-1550.4	-1801.9
**$NO3PA^{3-}$**	-5057.4	-4010.5	867.1	-193.0	-1721.1	-1914.0
**$PYTA^{4-}$**	-5911.3	-4735.0	853.7	134.9	-2164.8	-2029.9
**$OCTAPA^{4-}$**	-5962.2	-4882.4	769.1	-28.6	-1820.2	-1848.9
**$TPAEN^{4-}$**	-5943.2	-4699.7	759.9	-323.7	-1679.6	-2003.3
**$TPADAC^{4-}$**	-5933.2	-4671.0	756.5	-353.6	-1665.0	-2018.6
**$DTPA^{5-}$**	-6793.6	-5530.3	852.4	-421.4	-1694.4	-2115.8
**$TTHA^{6-}$**	-7487.2	-5872.3	778.3	-382.3	-2010.8	-2393.2
**$HOPO^{4-}$**	-5883.7	-4771.8	812.2	-48.9	-1875.2	-1924.1
**$BISPI^{2-}$**	-4225.5	-3124.9	850.9	-499.6	-1451.8	-1951.5

Table S19: Interaction energies and values of the EDA terms calculated for the Ac^{3+} complexes in kJ·mol^{-1}.

Ligand	E_{Int}	E_{Elec}	E_{Pauli}	E_{Ind}	E_{Dis}	E_{Pol}
**H_2O**	-2964.2	-2259.1	951.6	-226.0	-1430.8	-1656.8
**$DOTA^{4-}$**	-5784.8	-4976.2	1156.4	821.4	-2786.4	-1965.0
**$DO3APIC^{4-}$**	-5782.3	-4919.9	1173.0	742.8	-2778.3	-2035.4
**DOTAM**	-2411.2	-1620.4	1095.8	-3.4	-1883.1	-1886.6
**$TETA^{4-}$**	-5758.2	-4920.9	1133.4	736.8	-2707.5	-1970.7
**$MACROPA^{2-}$**	-4153.1	-3173.7	980.9	171.4	-2131.7	-1960.3
**$BP15C5^{2-}$**	-4056.7	-3189.5	1141.8	377.5	-2386.5	-2009.0
**$BP12C4^{2-}$**	-4042.9	-3158.8	1075.0	375.5	-2334.5	-1959.0
**$NO3PA^{3-}$**	-4913.5	-4010.5	1172.2	633.8	-2709.0	-2075.2
**$PYTA^{4-}$**	-5765.3	-4832.7	1152.0	785.5	-2870.0	-2084.6
**$OCTAPA^{4-}$**	-5832.4	-4932.5	1045.5	736.6	-2681.9	-1945.3
**$TPAEN^{4-}$**	-5811.0	-4803.7	1062.0	613.8	-2683.0	-2069.2
**$TPADAC^{4-}$**	-5800.0	-4779.4	1062.7	616.8	-2700.2	-2083.4
**$DTPA^{5-}$**	-6714.5	-5881.4	1134.9	976.1	-2944.1	-1968.0
**$TTHA^{6-}$**	-7515.5	-6501.6	1048.6	935.2	-2997.7	-2062.5
**$HOPO^{4-}$**	-5735.2	-4740.9	1096.9	534.5	-2625.6	-2091.1
**$BISPI^{2-}$**	-4077.6	-3109.7	1160.0	226.6	-2354.4	-2127.9

Table S20: Cartesian coordinates (Å) obtained from geometry optimizations for $[La(H_2O)_9]^{3+}\cdot 21H_2O$.

E -2325.852228112971 E_h

La	-0.17264729881948	0.17750533804630	0.11446049169001
O	0.29589342308430	-1.02937021072595	-5.42692187960791
H	-0.55411113172340	-0.95268099112942	-4.94534740875401
H	0.95036548063597	-0.52873482724091	-4.89341260194854
O	-2.15834870035187	-0.30300127694398	-1.44943675633771
H	-2.13278181042020	-0.64734565338810	-2.37777541900343
H	-2.93888442456182	-0.70241515382035	-1.02232507344904
O	-0.83246100956008	2.37089818003163	-1.16677169754508
H	-0.23116108188172	2.73695053946049	-1.85700045584298
H	-0.96099105862402	3.11255236589201	-0.53768251640010
O	0.57260141980232	-1.88987312604652	-1.27111588855246
H	0.58791210382844	-2.23422431321022	-2.19898429172260
H	0.68830954192252	-2.68804949271603	-0.71179654518986
O	-0.20201162841010	0.03839126396742	2.67840764256434
H	0.63354417408814	0.13508654317935	3.18014643134578
H	-0.93869451608099	0.38524426437633	3.23012132752488
O	1.29635262364892	2.09464482145701	1.18094896587394
H	0.88293263778030	2.50899908609224	1.97053230606266
H	1.46866378737204	2.85808785841420	0.57900539814883
O	-1.55041086305344	-1.88911111999288	0.99812230630116
H	-1.10078492375859	-2.32960736928414	1.75517125646529
H	-1.67946938310885	-2.61502023990060	0.34259804239403
O	1.83812739026652	-1.09492022955670	1.10998077355796
H	2.18991810564940	-0.75787302187993	1.96263017879614
H	2.61721824065601	-1.22248723944464	0.52010552346363
O	-2.21488883584521	1.38012432580083	1.11558798013720
H	-2.43558574061307	1.26892487637209	2.06566354114297
H	-3.07737252310892	1.43779651055602	0.63899268064020
O	1.77204969807329	0.92015252325202	-1.38331551016819
H	1.99026970859666	0.54255824485794	-2.27136639650550
H	2.62016053215591	1.07621969646211	-0.92694985307782
O	-4.67504006016575	1.36259584381675	-0.09771289592519
H	-4.65753307098213	0.38472471230992	-0.03266384225624
H	-4.50459994097875	1.54271005077001	-1.03149334977513
O	-4.21417261689080	-1.33408093053913	0.32142410679068
H	-3.38360297122665	-1.54504180567385	0.79508761341516
H	-4.49246355490080	-2.16190751832520	-0.09036721368339
O	0.66571319154250	-4.20745725973687	0.27469759661317
H	1.34451381335169	-4.89278180710704	0.25800535949376
H	0.49858859581392	-3.99580934801325	1.22419130882139
O	-1.85689475815118	-4.07552514425330	-0.66960825778705
H	-0.94595416952260	-4.33991958084558	-0.39435077782581
H	-2.45341466608752	-4.70863220034865	-0.24968842478919
O	3.93873415877713	1.41029522558555	0.45590181799009
H	4.28168462102472	2.25805855137643	0.14556254225664
H	3.12873761668901	1.62965909260732	0.95956570289971

O	4.09660221432631	-1.22658132865183	-0.47261209448326
H	4.20424555060780	-0.28226783202911	-0.23902063374288
H	3.72652656882390	-1.20810372373062	-1.36554298200002
O	-0.87345010075197	4.53239803814936	0.61444911472033
H	-0.65484737990736	4.28965084675132	1.54604042907116
H	-1.47214359455454	5.28750986050543	0.65862668244894
O	1.64966931283451	4.36264642822448	-0.35909523741521
H	2.28036815506935	5.00030077771766	-0.00191191887787
H	0.76205565943697	4.64882035247595	-0.03672773789545
O	2.29482364182158	0.12712361947074	-3.89521447119164
O	-2.09703509051156	-1.21935364186110	-3.97764556189618
H	-2.14112018375008	-2.19509575438830	-3.82266691276309
H	-2.91730950243548	-0.98833246461831	-4.43185994078343
H	3.08677954774448	-0.41988229878082	-3.97652544966253
H	2.54114402805219	1.01791581919517	-4.24452449101129
O	2.31754956034725	-0.22293064769662	3.65012149110240
O	-2.44045318890291	1.03123145521685	3.81114097322600
H	3.02654821613783	0.28911615724029	4.05612174105761
H	2.25208436977726	-1.07432175451254	4.13933306668929
H	-2.30722363501151	1.84895848899342	4.34370882597032
H	-3.07424551386954	0.48172336050799	4.28700793612689
O	0.71668410408941	-3.17300732737319	-3.67781653460755
O	-1.94987878305337	-3.85051554622018	-3.35582042616093
H	0.64844822218170	-2.48124199460345	-4.37274450851113
H	-0.19083354999892	-3.54289483358913	-3.63385665542881
H	-2.43578415178919	-4.57546409216012	-3.76674961526141
H	-1.98607721718604	-4.00165731478427	-2.37867145585571
O	0.77381256763516	3.78429905240271	-2.91928673377748
O	2.71442468228991	2.68386405730757	-4.66208573056727
H	1.45124774583714	3.37866246658164	-3.49586946897612
H	1.25738418782696	4.07778661937788	-2.11955702664171
H	3.60223733266445	3.02697967776619	-4.49319712052809
H	2.54525043951416	2.87270527053983	-5.59494177623413
O	-0.12756713086257	-3.44540490069274	2.77378618494466
O	1.75593247681361	-2.68913976817274	4.64209339885237
H	0.53165231153415	-3.16933682313211	3.45165571797962
H	-0.66036504495820	-4.13208036305460	3.19418792278866
H	2.48663124412479	-3.32250798106022	4.65545632150095
H	1.39862649903132	-2.70028938375524	5.54051835883208
O	0.05637576741123	3.70608514316505	3.04735572468213
O	-1.80226298092545	3.35824897602302	5.03509959838265
H	0.68960879919387	4.36358218081163	3.36256941417103
H	-0.58514217998101	3.58590564042906	3.78483476058674
H	-2.47355268528138	4.05358781045227	5.05397903176491
H	-1.46453941732794	3.30280362100102	5.93899395113220

Table S21: Cartesian coordinates (Å) obtained from geometry optimizations for $[Ac(H_2O)_9]^{3+}\cdot 21H_2O$.

E -2669.768254409089 E_h

Ac	-0.16647293831610	0.17334171627980	0.10614046504883
O	0.31385516847208	-1.03896623123523	-5.43102440497318
H	-0.53994406953684	-0.96384808692083	-4.95596390995763
H	0.96154852381327	-0.52359737416390	-4.90356910738390
O	-2.20393048573505	-0.33538500768954	-1.48585068378662
H	-2.17998410209206	-0.66854337621341	-2.41829025062546
H	-2.98114905369081	-0.74416439266997	-1.05721491421083
O	-0.87226654667194	2.42206063897554	-1.19482931735752
H	-0.28313769320886	2.78798473168099	-1.89663586924230
H	-0.98801637470021	3.16529426297960	-0.56449676756834
O	0.64628580132648	-1.94795188945070	-1.28021094549484
H	0.65453771156936	-2.29493057434610	-2.20844962890880
H	0.74244174745996	-2.74715636901732	-0.71795140448382
O	-0.19208049559698	0.04759163421329	2.73001007118989
H	0.63705448336306	0.06700329439771	3.25243776540463
H	-0.92386871343381	0.38940921707851	3.29112793493090
O	1.33661333924485	2.16350326997724	1.18403337112256
H	0.92572195573602	2.58518254845442	1.97131817527143
H	1.49580646108174	2.92138909206460	0.57067932325604
O	-1.58552734540571	-1.94861170137012	1.01127845844654
H	-1.14249158712940	-2.39229003636117	1.77049377669028
H	-1.70782049022892	-2.67375261593585	0.35255851226821
O	1.90351232632718	-1.11303738713820	1.15361440036027
H	2.22797670028932	-0.81630490568410	2.03151671712188
H	2.69904460723699	-1.21807519765396	0.58089429123391
O	-2.24727311922403	1.41476887785047	1.16577786588163
H	-2.44773930043051	1.31081914796859	2.12137081901514
H	-3.11948845474797	1.45976712384816	0.70460269437288
O	1.82829645685346	0.95120088588227	-1.41610845839513
H	2.04743388001322	0.58908708002030	-2.31099141916865
H	2.67472025024505	1.12142108565796	-0.96101429434525
O	-4.70318716884620	1.36351900953868	-0.04488298074289
H	-4.67646363292043	0.38452110024899	-0.00931077683514
H	-4.53106788860214	1.57470285800123	-0.97185043989575
O	-4.21808571267355	-1.34370281970055	0.27077378781182
H	-3.41181989718425	-1.58201538862977	0.77219097762888
H	-4.57849577766826	-2.17782679099860	-0.05501795061466
O	0.67222311192003	-4.24924582924500	0.28416882643487
H	1.33278567896311	-4.95236781594201	0.27534234657464
H	0.48637459216167	-4.04619303700213	1.23205844830043
O	-1.84378754472583	-4.11039132185176	-0.68581327479007
H	-0.93337356705976	-4.37089155090327	-0.40428710788738
H	-2.43836919138887	-4.75937312131846	-0.28777481690945
O	3.96369920678666	1.46850276086943	0.43879421165596
H	4.31968532329621	2.31264604340487	0.13367632240933
H	3.15209529190158	1.69539642278828	0.93733307115045

O	4.16251120321114	-1.18061848952560	-0.42773671306654
H	4.24443721606908	-0.22837250676652	-0.21578851118376
H	3.78120811346523	-1.19326528774389	-1.31603217023328
O	-0.87547821509837	4.57528280519613	0.59292860572073
H	-0.64533017109547	4.34370194750308	1.52461213519170
H	-1.46521221282756	5.33756469686582	0.63561392731964
O	1.64148133357156	4.40068169940900	-0.40540822139311
H	2.26877130661128	5.05549722748044	-0.07394648408640
H	0.75484024268748	4.68438806610189	-0.07798889365908
O	2.31190598909558	0.16842251231739	-3.93513843385712
O	-2.10086772899442	-1.23892175662987	-4.01802119303725
H	-2.13190667807832	-2.21409286748037	-3.85613965447978
H	-2.91247585450833	-1.02565786245411	-4.49603186325191
H	3.11494193429181	-0.35831000909143	-4.03889572471645
H	2.52341417382115	1.06236919237776	-4.29943215664912
O	2.29190362714706	-0.35656040706826	3.74578713692818
O	-2.41364854021477	1.07171773894073	3.87189565067424
H	2.97665744248735	0.14561092822877	4.20258196874634
H	2.19311752376080	-1.21454225466790	4.21845760120081
H	-2.26440549298575	1.89891943774739	4.38560560164205
H	-3.05987910593648	0.54789296556345	4.35999039521920
O	0.74601142956781	-3.19065068601361	-3.70228635282366
O	-1.91998721208469	-3.86314387246807	-3.37314451215702
H	0.67384396007028	-2.48692112367265	-4.38555367071393
H	-0.16353184754230	-3.55589570341504	-3.65404971605743
H	-2.39670703749623	-4.59335878816445	-3.78569748462053
H	-1.95738691790141	-4.01613720111365	-2.39628845834594
O	0.73073541885256	3.81344212897689	-2.95449821411391
O	2.64511375327519	2.72720772044562	-4.73260896033017
H	1.39848472211184	3.40908111576934	-3.54331769068655
H	1.22608018252389	4.10458728940016	-2.16101400751903
H	3.52866373686509	3.08904394384762	-4.58146446104938
H	2.45587957219665	2.90655256217019	-5.66344863473138
O	-0.17010792999837	-3.51719894387535	2.77946024202221
O	1.67390063806876	-2.82043287242804	4.70466539505374
H	0.47541453671551	-3.26246972453453	3.47885402685593
H	-0.71151598312760	-4.21527343666324	3.16935687236428
H	2.39659887263905	-3.46270233827054	4.72639875965273
H	1.29782976867790	-2.83586187441195	5.59533639237874
O	0.08280300481643	3.78834287122512	3.03010986612180
O	-1.74573578290300	3.42002410979686	5.04108574879686
H	0.70671445701366	4.45933047924994	3.33546537835819
H	-0.54939568386447	3.66107614390128	3.77467984542180
H	-2.42241887866806	4.11001880373910	5.06469120976234
H	-1.39225235313138	3.37694763946615	5.93966151332744

Table S22: Cartesian coordinates (Å) obtained from geometry optimizations for $[La(DOTA)(H_2O)]^-$.

E -1554.283594116957 E_h

La	-0.10614274237991	0.00705122101595	-0.28722252346457
N	0.10580991361297	1.76032297871255	1.82326759095231
C	1.50802361531175	2.15227909579594	2.08316255283513
C	2.28734919617612	2.37489195801694	0.80293409969374
N	2.38012149758062	1.15383793886856	-0.03144853445337
C	3.37651986554621	0.22037882477440	0.53699522062496
C	3.21818436043189	-1.18887464352452	0.00371937865233
N	1.89802393528174	-1.78036943301000	0.32575248432429
C	1.85384521162307	-2.19836160645069	1.74334319240989
C	0.43709745591862	-2.41049772925763	2.23731414748650
N	-0.38520333074004	-1.17861030948381	2.17142665845244
C	-0.02525031270354	-0.26495388131606	3.27819732640894
C	-0.49574750010526	1.15420236920000	3.03436282158350
C	-0.69712659767984	2.93342580665301	1.44424232481057
C	-2.03952892294575	2.54682322913998	0.82926043791130
O	-2.12999114732358	1.38031922002444	0.31118926610930
C	2.75654348156649	1.54875742765667	-1.39796135273167
C	1.60996315809357	2.22134638615408	-2.15064957135463
O	0.42014002530126	1.97798806029514	-1.74456391887131
C	1.67399456904343	-2.93084584094413	-0.56351483550977
C	1.35877789605448	-2.50597644759006	-1.99560858239800
O	0.89322660523904	-1.32552686058578	-2.16531152257215
C	-1.80458989726005	-1.56048083518625	2.25470781295374
C	-2.30754384179823	-2.21978956760156	0.97200286554833
O	-1.68938659318535	-1.93314318751848	-0.11237285682195
H	1.97332384036979	1.36370943629574	2.67270543656199
H	1.54281462116003	3.06721023464187	2.68751417506362
H	1.80763453089704	3.14870009999810	0.20286132917141
H	3.29211771966833	2.73880083396744	1.05147732756252
H	3.25992539645838	0.22493065948678	1.61974229325477
H	4.39369299319819	0.57127227740799	0.32305982920453
H	3.32578917156150	-1.19211396175944	-1.08130759718451
H	4.02109904786578	-1.81794257029318	0.40787283642143
H	2.34411642775444	-1.42699031777725	2.33532239998155
H	2.42475750590725	-3.12403045514680	1.88714771576359
H	-0.05921179558979	-3.17153796474434	1.63429753029504
H	0.46769736794550	-2.78714934816967	3.26721383519010
H	1.05768517882228	-0.28445782588447	3.39007958439954
H	-0.45119346928598	-0.62500928079078	4.22284174316669
H	-1.57875373622243	1.17410034834754	2.90863542016409
H	-0.26381999383865	1.76502383458588	3.91584511570671
H	-0.86372522294173	3.59434843180139	2.30214340080409
H	-0.16026338702166	3.50968675922395	0.68755813816685
H	3.62919697711974	2.21136600165082	-1.39479955864094
H	3.02462026220087	0.65746461140184	-1.96994494128437
H	2.53548259414557	-3.60786589920493	-0.56277223432046

H	0.81144105833183	-3.49950415076038	-0.20876583344494
H	-1.98651540998303	-2.22696336299107	3.10487565451875
H	-2.41041952055648	-0.66388185667071	2.40514447465507
O	-1.84775747408968	-0.01150097543917	-2.33251102662191
H	-2.39910508578841	0.77999675029908	-2.26051073072648
H	-2.44487864902917	-0.74549457471088	-2.12680529556011
O	-2.95258252755828	3.39748655277520	0.83247830783894
O	-3.30566636976930	-2.96346383098784	1.04520827844187
O	1.53272298432889	-3.33932629344151	-2.90726093627093
O	1.89438006327943	2.93251566305010	-3.13471715485843

Table S23: Cartesian coordinates (Å) obtained from geometry optimizations for $[Ac(DOTA)(H_2O)]^-$.

E -1898.192751030179 E_h

Ac	-0.16560177205017	0.00367279410013	-0.35619417929416
N	0.09193339047480	1.76932869832780	1.83174505929095
C	1.49934805604978	2.15081860684908	2.08194894275674
C	2.27766155882362	2.38190460585377	0.80086625181382
N	2.38630892371763	1.16768295480740	-0.04072495282003
C	3.37033082607283	0.22632096568978	0.53750562939502
C	3.22205002874039	-1.18319828053342	-0.00230868847793
N	1.91064889179334	-1.79361923046681	0.31715079679514
C	1.86158127206151	-2.19832519924845	1.73886915935451
C	0.44622834348323	-2.41976831585393	2.23758451441616
N	-0.38849855733738	-1.19675692611940	2.18352726413763
C	-0.01863197016504	-0.27155957921268	3.27734215443856
C	-0.50102737601373	1.14633892786831	3.03782050568714
C	-0.70194066077550	2.95725488880172	1.48052389690837
C	-2.05783199499790	2.61094496279132	0.87018606558690
O	-2.17278999773510	1.46682412140284	0.30847474890144
C	2.79809629373859	1.57049710351916	-1.39467284884095
C	1.68019641286067	2.24914810594716	-2.18509020927096
O	0.47609520100515	1.99614452572452	-1.83062214232561
C	1.71120332527698	-2.95998785473187	-0.55688707546922
C	1.39316645769263	-2.57584077790512	-2.00067669470642
O	0.91400912705549	-1.40596137985949	-2.20364509832444
C	-1.80266242082637	-1.58783231949149	2.30030701654109
C	-2.34138847605384	-2.25970214050175	1.03788571473064
O	-1.75218803309924	-1.98854456308266	-0.06638370014033
H	1.96486716215709	1.35683103030647	2.66362380581632
H	1.54375100926720	3.06133568473218	2.69265781856374
H	1.79350279880098	3.15460591009522	0.20221364269005
H	3.27803245483104	2.75520501846565	1.05513300028279
H	3.23953245258663	0.22583800682798	1.61838865615991
H	4.39175509660223	0.57514402067717	0.34007447462312
H	3.32919261932286	-1.18211564469393	-1.08767584487938
H	4.03414731019087	-1.80341940258831	0.39839663938700
H	2.34515538734057	-1.41942126006314	2.32604776063527
H	2.43850768877804	-3.11880539230408	1.89328263960055

H	-0.04833694008021	-3.18254009288710	1.63494306438082
H	0.48807343221323	-2.80234733745385	3.26542568724336
H	1.06554310623592	-0.28380808524201	3.37598968470058
H	-0.42896317157511	-0.62735659928024	4.23078364227781
H	-1.58467511452971	1.15814712342781	2.91393194928755
H	-0.27416408505765	1.75149896266528	3.92504074422861
H	-0.84726618395834	3.60783441413696	2.35030020682896
H	-0.16446112184690	3.53697407843156	0.72618358723354
H	3.67412956550212	2.22831418154004	-1.36450440576825
H	3.07894213955298	0.68055032924564	-1.96349330502476
H	2.58293779536925	-3.62401356396493	-0.53969259150197
H	0.85650563450603	-3.53751335473043	-0.19626118699369
H	-1.96206906657194	-2.24724053409985	3.16083321194467
H	-2.40973805536949	-0.69281556366270	2.45760986862464
O	-1.97603717587860	0.00168200634771	-2.45539160597212
H	-2.48011204774630	0.82724612843936	-2.44302698719407
H	-2.63221113402968	-0.68776775655870	-2.28059864821491
O	-2.95691666921431	3.47552052354039	0.91381797461230
O	-3.34239423297565	-2.99520770120148	1.14794635725822
O	1.57581191018964	-3.43357648132868	-2.88759557067748
O	2.00236958559485	2.96901265650410	-3.15105740123721

Table S24: Cartesian coordinates (Å) obtained from geometry optimizations for $[La(DO3APIC)]^-$.

E -1898.192751030179 E_h

Ac	-0.16560177205017	0.00367279410013	-0.35619417929416
N	0.09193339047480	1.76932869832780	1.83174505929095
C	1.49934805604978	2.15081860684908	2.08194894275674
C	2.27766155882362	2.38190460585377	0.80086625181382
N	2.38630892371763	1.16768295480740	-0.04072495282003
C	3.37033082607283	0.22632096568978	0.53750562939502
C	3.22205002874039	-1.18319828053342	-0.00230868847793
N	1.91064889179334	-1.79361923046681	0.31715079679514
C	1.86158127206151	-2.19832519924845	1.73886915935451
C	0.44622834348323	-2.41976831585393	2.23758451441616
N	-0.38849855733738	-1.19675692611940	2.18352726413763
C	-0.01863197016504	-0.27155957921268	3.27734215443856
C	-0.50102737601373	1.14633892786831	3.03782050568714
C	-0.70194066077550	2.95725488880172	1.48052389690837
C	-2.05783199499790	2.61094496279132	0.87018606558690
O	-2.17278999773510	1.46682412140284	0.30847474890144
C	2.79809629373859	1.57049710351916	-1.39467284884095
C	1.68019641286067	2.24914810594716	-2.18509020927096
O	0.47609520100515	1.99614452572452	-1.83062214232561
C	1.71120332527698	-2.95998785473187	-0.55688707546922
C	1.39316645769263	-2.57584077790512	-2.00067669470642
O	0.91400912705549	-1.40596137985949	-2.20364509832444
C	-1.80266242082637	-1.58783231949149	2.30030701654109
C	-2.34138847605384	-2.25970214050175	1.03788571473064

O	-1.75218803309924	-1.98854456308266	-0.06638370014033
H	1.96486716215709	1.35683103030647	2.66362380581632
H	1.54375100926720	3.06133568473218	2.69265781856374
H	1.79350279880098	3.15460591009522	0.20221364269005
H	3.27803245483104	2.75520501846565	1.05513300028279
H	3.23953245258663	0.22583800682798	1.61838865615991
H	4.39175509660223	0.57514402067717	0.34007447462312
H	3.32919261932286	-1.18211564469393	-1.08767584487938
H	4.03414731019087	-1.80341940258831	0.39839663938700
H	2.34515538734057	-1.41942126006314	2.32604776063527
H	2.43850768877804	-3.11880539230408	1.89328263960055
H	-0.04833694008021	-3.18254009288710	1.63494306438082
H	0.48807343221323	-2.80234733745385	3.26542568724336
H	1.06554310623592	-0.28380808524201	3.37598968470058
H	-0.42896317157511	-0.62735659928024	4.23078364227781
H	-1.58467511452971	1.15814712342781	2.91393194928755
H	-0.27416408505765	1.75149896266528	3.92504074422861
H	-0.84726618395834	3.60783441413696	2.35030020682896
H	-0.16446112184690	3.53697407843156	0.72618358723354
H	3.67412956550212	2.22831418154004	-1.36450440576825
H	3.07894213955298	0.68055032924564	-1.96349330502476
H	2.58293779536925	-3.62401356396493	-0.53969259150197
H	0.85650563450603	-3.53751335473043	-0.19626118699369
H	-1.96206906657194	-2.24724053409985	3.16083321194467
H	-2.40973805536949	-0.69281556366270	2.45760986862464
O	-1.97603717587860	0.00168200634771	-2.45539160597212
H	-2.48011204774630	0.82724612843936	-2.44302698719407
H	-2.63221113402968	-0.68776775655870	-2.28059864821491
O	-2.95691666921431	3.47552052354039	0.91381797461230
O	-3.34239423297565	-2.99520770120148	1.14794635725822
O	1.57581191018964	-3.43357648132868	-2.88759557067748
O	2.00236958559485	2.96901265650410	-3.15105740123721

Table S25: Cartesian coordinates (Å) obtained from geometry optimizations for $[Ac(DO3APIC)]^{-}$.

E -2068.935384654263 E_h

Ac	-0.20118455644172	0.01516304703979	-0.58913452275972
N	0.66491666959723	1.70005356795743	1.57764634001452
C	-0.26452485979447	2.85475100648822	1.67685302994278
C	-1.72903025061119	2.46309256018966	1.63042599805135
N	-2.16206495973276	1.84422971919703	0.35562931648459
C	-3.44717607950157	1.13535712678670	0.57011571234632
C	-3.28602888995422	-0.16314342566822	1.33846166871428
N	-2.45749152255650	-1.17769466302499	0.64649255402169
C	-2.01065160027948	-2.19767950921732	1.62617109622766
C	-0.89852426734331	-1.70141905428779	2.53330399253401
N	0.35803025311087	-1.32805749782210	1.83596644153406
C	1.17519978789990	-0.47672530066904	2.73518868820874
C	0.64358664145677	0.93936875062901	2.85077319119959

C	-2.33457537148179	2.87620094347719	-0.68286889624732
C	-1.05461406582069	3.27352578471888	-1.41669545852484
O	-1.00205534280607	4.41169080485690	-1.92573277809400
O	-0.13867840516595	2.38635442375720	-1.52022968233455
C	-3.23146663230440	-1.82304192871354	-0.42907942617252
C	-3.26698471683254	-1.05280265603305	-1.74869887130885
O	-4.24973972480322	-1.21896994749245	-2.49775055552332
O	-2.24791045335790	-0.33124328809469	-2.03670136486192
C	1.12885255062776	-2.53500503532093	1.48356205843569
C	0.64187613151184	-3.25504419296924	0.22853686435979
O	0.89480166465207	-4.47399299192919	0.11978987476521
O	0.05768059283137	-2.54802334474112	-0.65799718910480
H	-2.81406017724626	3.77265348497105	-0.27563137024244
H	-2.99411770966252	2.47333146406978	-1.45621009503701
H	-4.25572458002687	-2.03791563875750	-0.10669936405578
H	-2.75210475762004	-2.77617122124139	-0.66661570765860
H	2.15587324863658	-2.22821814789584	1.26481947792757
H	1.16555235385028	-3.23934807184374	2.32145535168077
H	-0.02914021609464	3.53838132167900	0.86256313181588
H	-0.08935391200549	3.40043801491139	2.61331653203410
H	-2.32698746075794	3.36356192275460	1.82127566661672
H	-1.95002846313504	1.76420752834775	2.43449016327565
H	-4.14991681018762	1.77452037998411	1.12056567170640
H	-3.88976140691428	0.94656667544680	-0.40700154193894
H	-4.28454022598099	-0.57528475559815	1.53375593970552
H	-2.83165015163681	0.03532084527708	2.30755574460785
H	-2.84921370626680	-2.50935165673944	2.26264917592774
H	-1.68967580867944	-3.07596741357674	1.07019674100101
H	-1.24255149550691	-0.82926253499446	3.08620616083613
H	-0.68359356066950	-2.48234449475386	3.27366134369557
H	2.19441582430747	-0.47306881612621	2.35212568834145
H	1.21452370174035	-0.91306311964460	3.74158956814868
H	-0.38384490110772	0.91571889442505	3.20810428296481
H	1.23124390358086	1.47202112922606	3.60879313023579
C	2.02534812413575	2.22504061675837	1.32040993735690
H	1.92689088715363	3.04344156380316	0.60336777635158
H	2.46086130142822	2.63831793964271	2.23640586839332
C	2.96476028646730	1.22325788990171	0.70814049213504
C	4.28449472096576	1.08193109969983	1.12703617812661
C	3.28364098039314	-0.31172019986441	-0.99899932634549
C	5.11010485306310	0.19325838603577	0.45075698224811
H	4.64708157273573	1.65419668601869	1.97077013111516
C	4.60773870397780	-0.51470547170544	-0.63535851808870
H	6.13754861907133	0.05732314752088	0.76435622271360
H	5.22236366253961	-1.20508249994534	-1.19536835005517
N	2.48347060921488	0.52887539363508	-0.32837786193824
C	2.65727242301232	-0.98994755019255	-2.20132288499367
O	3.34019482267776	-1.80903185028651	-2.84736856498613
O	1.45655915164695	-0.64556884005706	-2.47169785553027

Table S26: Cartesian coordinates (Å) obtained from geometry optimizations for $[La(DOTAM)(H_2O)]^{3+}$.

E -1476.619426126964 E_h

La	0.11060595869608	-0.15367423143385	-0.35585674050686
N	-0.84617256933859	-0.04454942892460	2.22665030303239
C	-0.29427508596707	1.11042847739918	2.97576591627963
C	-0.17419710310925	2.35424648761264	2.12040958217960
N	0.73720252879322	2.17126096892077	0.95977886961306
C	2.14992273306177	2.18606939495881	1.41115744804383
C	3.09842120286084	1.61118786598509	0.38047414278206
N	2.81153128085407	0.18717280740968	0.06279729828063
C	3.32070273106515	-0.68953698299796	1.14461873998434
C	2.69180535366504	-2.06683717685241	1.12334880432542
N	1.21854730788658	-2.03294215195764	1.31959070338174
C	0.89749779205221	-1.75935049322790	2.74101223906721
C	-0.53550659213804	-1.31262678924886	2.93816803054649
C	-2.30766021497301	0.07977568764190	2.11739934585370
C	-2.83493753695012	-0.90769636331931	1.10323252708098
O	-2.11530672125549	-1.27496661568877	0.14452832180986
C	0.49761064664123	3.26467121208053	0.00533592906059
C	-0.83092582824283	3.06295904589054	-0.68534981032002
O	-1.28437065999280	1.90650571651047	-0.86011550095221
C	3.46994460781731	-0.14819410519803	-1.20935436127297
C	2.73901107659268	0.51225729035564	-2.35406389987044
O	1.50919397859127	0.74137856136388	-2.26976969923770
C	0.66774696693086	-3.33542987345477	0.91565671930284
C	0.69496301587776	-3.46154094940843	-0.58971578373841
O	0.61633949841349	-2.44054159976569	-1.31359500000830
H	0.68117197376517	0.82016426840275	3.36056776092930
H	-0.92570140628957	1.33499071630531	3.84259714552447
H	-1.15356708005514	2.63788847537189	1.73218888949621
H	0.17722289344623	3.18524671922381	2.74247893362809
H	2.20845875555730	1.61329408070512	2.33457884465253
H	2.45955568302245	3.21027988238399	1.64739597694024
H	3.03510950936990	2.18106015234598	-0.54713687401523
H	4.12660969789482	1.71101609786200	0.74565748013631
H	3.11765632399165	-0.19633033377168	2.09288343819977
H	4.40842938048612	-0.79458863005832	1.06509701690130
H	2.88812791309147	-2.55539368972617	0.16796175416973
H	3.15639543505156	-2.68426313067319	1.90052026054332
H	1.58167488716366	-0.98983813708543	3.09292394820435
H	1.07901518572187	-2.65330329657137	3.34815893753082
H	-1.22160586556839	-2.07687969307821	2.57126408788888
H	-0.73121861435826	-1.19612511536164	4.00988375662541
H	-2.80043368492686	-0.05468868033492	3.08649458009709
H	-2.56351091031596	1.07621543165286	1.74917501355927
H	0.54132354362213	4.24450207420944	0.49231258586903
H	1.26192588201531	3.24509086905446	-0.77530155711607
H	4.52777365035980	0.13510051596208	-1.20525493620230

H	3.41418910121724	-1.22629017672611	-1.37858690016087
H	1.20375139887765	-4.16612996989638	1.38701705556824
H	-0.37991936793968	-3.40110937513525	1.21713045764704
O	-1.19570401348264	-0.47982387217124	-2.61113641246543
H	-1.70578771388396	0.30906184258195	-2.84306877212108
H	-1.84593053738458	-1.19508297977368	-2.56979338033443
N	-4.07896339297089	-1.32948951810391	1.25473852578787
N	0.76943087375612	-4.67915549148215	-1.09912796981783
N	3.43655835700636	0.80265542561952	-3.43886189135268
N	-1.46174973795596	4.14048147033720	-1.11915232644432
H	0.85100916964082	-5.48741357920938	-0.49974477192642
H	0.72247467922779	-4.81238944916305	-2.10007994957751
H	-4.63022616125654	-1.03560805352631	2.04764208959286
H	-4.49653949655550	-1.93835169174492	0.56441585701407
H	-2.32123136680495	4.04930493449351	-1.64357052776459
H	-1.08781470228907	5.06234226942608	-0.94729446798547
H	4.43060731094283	0.63056721350231	-3.47741513259455
H	2.97183515897730	1.18312167950214	-4.25189744134496

Table S27: Cartesian coordinates (Å) obtained from geometry optimizations for $[Ac(DOTAM)(H_2O)]^{3+}$.

E -1820.528006362982 E_h

Ac	0.05401231048124	-0.15763132528681	-0.44395689050948
N	-0.86851923977711	-0.05532826223678	2.22830756768314
C	-0.30038133533054	1.09946214454330	2.96648519873515
C	-0.18906779798626	2.34844168029365	2.11469540042088
N	0.72332378157223	2.18610222272590	0.95238065437598
C	2.13711487939648	2.17924181854913	1.40237688249131
C	3.08791196558982	1.61687202174920	0.36460817700673
N	2.82029701663835	0.19166187610890	0.03841576931299
C	3.31345875612288	-0.68611663038734	1.12800647564693
C	2.68983842802533	-2.06688585129252	1.10093182146306
N	1.21754792021098	-2.04877451536478	1.30357546103945
C	0.89534676548929	-1.76353361761866	2.72274703476188
C	-0.54142776237262	-1.32660110704523	2.92537726654632
C	-2.33211613482611	0.07410735550157	2.15813786475939
C	-2.89900146896771	-0.90416055940783	1.15554624681241
O	-2.22676537567832	-1.24716854235539	0.15394170547786
C	0.50158846903205	3.30745071305562	0.02580278233725
C	-0.82420942706661	3.15361070632496	-0.68295446646677
O	-1.28204282438145	2.01325919666260	-0.93483044266214
C	3.51416425001928	-0.13947212457802	-1.21562953984587
C	2.81104821120717	0.49890321568662	-2.39055428381081
O	1.57175913603232	0.68974213307315	-2.36117054857003
C	0.68122911465230	-3.36481870198472	0.92430059567658
C	0.70388394028013	-3.53004101235599	-0.57713809634272
O	0.57544112942312	-2.53407286323335	-1.32884061472405
H	0.67944956534739	0.80713834592226	3.33762791449170
H	-0.91695803914584	1.32636256189598	3.84354311334616

H	-1.17078013467091	2.62631636340127	1.72692923027849
H	0.15371383717555	3.17808386725056	2.74388860217079
H	2.19193028691012	1.59205476261780	2.31657709235216
H	2.45486295395146	3.19688363596398	1.65644866423551
H	3.01652791808439	2.18960035884832	-0.56117623287783
H	4.11552031131856	1.72893539554465	0.72884410237874
H	3.09907367264226	-0.19413019441530	2.07413326115161
H	4.40237672790327	-0.79014555952816	1.06328522577133
H	2.88459220042142	-2.54754568256931	0.14069571527429
H	3.16403502255169	-2.68598962972581	1.87130928866613
H	1.57471343821243	-0.98701204899230	3.06794289342499
H	1.08491807893241	-2.65064629734873	3.33778246072792
H	-1.22295754155665	-2.09309888828992	2.55380177132041
H	-0.73346028362065	-1.22301068331425	3.99942371332722
H	-2.79762300079412	-0.05900935889888	3.14103151156234
H	-2.59195851021611	1.07347957185692	1.79999755409565
H	0.56156086647253	4.27296825178613	0.53930145214639
H	1.27019045073264	3.29475752699921	-0.75137820711873
H	4.56848395523021	0.15573388374024	-1.18470751560545
H	3.47676129221803	-1.21971033611781	-1.37822377806383
H	1.22502004296650	-4.17979218116984	1.41409500213986
H	-0.36593564379557	-3.43394905829519	1.22816994545776
O	-1.31119092005270	-0.50748845372603	-2.74164734492358
H	-1.78669451139186	0.27627527993713	-3.05103374840474
H	-1.97694553225303	-1.20915823633949	-2.71629672381450
N	-4.12944486098237	-1.34192999142786	1.36224459353993
N	0.82530019534416	-4.75761773169837	-1.05223327948007
N	3.54458885956740	0.80802172745320	-3.44596225390455
N	-1.44599602674175	4.25985096738370	-1.05330981827732
H	0.94775974235403	-5.54369470174059	-0.43064025743285
H	0.77497094049659	-4.92183017622861	-2.04847118357285
H	-4.64480316227723	-1.06556433535269	2.18512249896652
H	-4.57491418239435	-1.94295752253370	0.68243509977166
H	-2.30146639753779	4.20587428619657	-1.58953093098129
H	-1.06855198484076	5.16719402279380	-0.82216173840358
H	4.54445845724164	0.66805009373141	-3.44188537977722
H	3.10253428841075	1.17260620326258	-4.27876912557468

Table S28: Cartesian coordinates (Å) obtained from geometry optimizations for [La(TETA)(H_2O)]$^-$.

E -1632.931743465449 E_h

La	4.94822677248242	14.95078233741640	13.10153659830499
O	5.64435110753149	12.62966065136311	12.58303663678744
O	7.43669102018102	15.29930685848431	12.63736307841831
O	4.78261143961205	17.25206411648976	12.19232650217914
O	3.26029415535430	14.63065704979540	11.27227639833516
O	6.82760693590253	10.79108414796126	13.05901090544566
O	8.99141122805207	16.88000059155361	12.32506794423273
O	3.58743154231258	19.09740763772604	11.78127606356058

O	2.10870238211607	13.09778683565340	10.11473933410129
C	6.76072632761177	12.51312257672805	14.70231021845151
H	7.69131646238071	13.05461283448334	14.51584801778186
H	6.98503232619048	11.71115828601252	15.41328877112164
C	6.37073431624341	11.91258896950624	13.35571041644242
N	5.77214407677123	13.45161390700517	15.27609275488268
C	6.44827321624881	14.31687310129683	16.28059190369969
H	7.06617794329486	13.70057373816628	16.94575501188789
H	5.66568639724965	14.76321178846661	16.89363580927991
C	7.30849194692886	15.40197723961012	15.67331008895571
H	8.03643941845209	14.97264860454060	14.98624888904802
H	7.87464018948506	15.89353497676833	16.47487455812024
N	6.51106692499704	16.41297239744696	14.93271131703966
C	7.43070670089960	17.19843561670364	14.09194690514099
H	6.88710742600442	18.04370294443942	13.66503127272176
H	8.25288512502114	17.60735354981993	14.69232376140472
C	8.01331878516606	16.40636994478894	12.93285582984268
C	5.89408923872870	17.33905133018773	15.91644498577596
H	6.67371345503988	18.02379751634710	16.27378038897871
H	5.57084620924909	16.75161007889267	16.77580336611914
C	4.69827569800812	18.13530128377219	15.40912602644458
H	4.58294336966402	18.98391966345948	16.08802850749132
H	4.89221018257934	18.56589427204814	14.42586160747005
C	3.38013584226687	17.36574140910747	15.46142525591224
H	2.55613105321424	18.06973873465201	15.63595530698006
H	3.41518658137488	16.69064160674050	16.31810560938677
C	3.74600203500702	17.99049497968331	12.33303511124508
C	2.60034270068538	17.43459657066118	13.17357780614015
H	1.98276492598991	16.86566063441806	12.47458290572638
H	1.99009765402561	18.25887032765178	13.55735605878209
N	3.01415726530012	16.54069321866053	14.27712513403750
C	1.85197088746522	15.71256935437734	14.70178829698852
H	2.09719060930476	15.30162468355935	15.68103318295180
H	0.96868440431629	16.35071091051626	14.83132895687829
C	1.51316990014440	14.59063969658116	13.74681179610433
H	1.33910574943759	14.98002979602844	12.74498402638431
H	0.57682518999831	14.12092508047130	14.07400900315843
N	2.58629456127452	13.56576623273975	13.66997353169433
C	2.36381567369906	12.75783325248856	12.45780493108545
H	3.06485267249200	11.92030451501627	12.45441613154959
H	1.35023554419331	12.33874369933265	12.45053369564162
C	2.58312331819545	13.55112455572002	11.17649432946273
C	2.47085494960566	12.67236147901721	14.85075402593224
H	2.19976660044399	13.28542646933563	15.71053906329519
H	1.63548330560387	11.98278742151941	14.67524009446083
C	3.72538668129927	11.88428128993490	15.20725238983468
H	4.17065567423459	11.42112873222026	14.32581535256156
H	3.39828966291410	11.05869454386536	15.84417448295780
C	4.74179160110907	12.68037153409335	16.02441623381609
H	4.19795785396834	13.39688542868762	16.64226696684526

H	5.27118746442009	12.00145182396004	16.70528608908129
O	5.98322644867707	14.87249330096498	10.51050068212561
H	6.83929488239009	15.11492145575534	10.91671428719389
H	5.67601765718966	15.67383051530484	10.06609044231819

Table S29: Cartesian coordinates (Å) obtained from geometry optimizations for the $[Ac(TETA)(H_2O)]^-$.

E -1976.842809903068 E_h

Ac	4.97603620863272	14.94680921907813	13.02803524141358
O	5.71232650065785	12.55546351023194	12.59431849597079
O	7.50671746704715	15.37587624365550	12.59381931495616
O	4.71969945973847	17.32021855948240	12.15364582484555
O	3.24883008153616	14.51853328545035	11.18317299347997
O	6.83674582162481	10.70275479244966	13.15304699822931
O	9.07659174977848	16.95905867305643	12.39197878638145
O	3.50853926345508	19.17068622024447	11.81573739494040
O	1.97720063044008	13.00577110583889	10.13000950081326
C	6.78334235636677	12.48014596052366	14.73882429161371
H	7.71310951015205	13.02018930496849	14.54187661213004
H	7.00807190016489	11.69631574722140	15.47005054439759
C	6.40306521159613	11.84474578060181	13.40466793364020
N	5.79290760878779	13.43052712706718	15.28749841572756
C	6.46572757591223	14.30843044558704	16.28300484693054
H	7.08444968992788	13.70132524639925	16.95608502989073
H	5.68201249659656	14.75856820344516	16.89146608430941
C	7.32921076924836	15.39216229245658	15.67162171708329
H	8.05052661784001	14.96028572153509	14.97825311598955
H	7.90484664525184	15.87357418193824	16.47309348191682
N	6.54137441227468	16.41476071696756	14.93817212336650
C	7.46857061373192	17.22014533442604	14.12388612510127
H	6.92367728347123	18.06944470795648	13.70502691437641
H	8.27702606268535	17.62660977758336	14.74412308935381
C	8.07944999305503	16.46489477685980	12.95196135317637
C	5.89878691743311	17.32331587175671	15.92228267634785
H	6.66625098926818	18.00979390132142	16.30220775657752
H	5.56520238687039	16.72352578846537	16.76869925673194
C	4.70538054508429	18.12060125811910	15.40428738620555
H	4.59537699101407	18.97740308460019	16.07349293552522
H	4.90490498790765	18.53964779054014	14.41732046299006
C	3.37760341982448	17.36313327963825	15.45941660370489
H	2.56430583188636	18.07410711128647	15.65559898340906
H	3.41462566994515	16.67482285508896	16.30535974365974
C	3.68397038577916	18.05060702174367	12.33582475268082
C	2.55719342564862	17.46769924755804	13.18423546403078
H	1.93653716486492	16.90462084954194	12.48241735218017
H	1.94219709119374	18.27911936016081	13.58803662691001
N	2.98995792480189	16.55966083292470	14.26782992980965
C	1.83486419275707	15.72050107172165	14.68867176281154
H	2.08190919854817	15.30690289080569	15.66607293322850

H	0.94616231576774	16.35045909199433	14.82226024476616
C	1.49856885742440	14.59933124685055	13.72835473850007
H	1.34019367673528	14.99027542739256	12.72369550741062
H	0.55237143956636	14.14074147766236	14.04404048873782
N	2.55892781765922	13.56215597420667	13.65896160714162
C	2.31223220610732	12.72700688866700	12.46975611570279
H	3.00984626276453	11.88575812530783	12.47764721547163
H	1.29751346723378	12.31135662659886	12.49167619090987
C	2.51484359431553	13.47219163686211	11.15539374695433
C	2.46171653163606	12.69316098284258	14.85988132843907
H	2.21198741903370	13.32367101967796	15.71308800462379
H	1.61817109353994	12.00625688052074	14.71490009118227
C	3.71785849130170	11.89871429988829	15.20613612737564
H	4.15740459082620	11.44399897225518	14.31753530815396
H	3.39002251228450	11.06645979798607	15.83367051122487
C	4.74442984390681	12.67893628429512	16.02980778648995
H	4.20930663415713	13.40608235782371	16.64267895031573
H	5.25491309368613	11.99082741903732	16.71612598869610
O	6.04909478947863	14.91363798594617	10.43202782855415
H	6.90595545564530	15.19950498893734	10.80299809910450
H	5.72350852412854	15.66713146494790	9.92183831340620

Table S30: Cartesian coordinates (Å) obtained from geometry optimizations for $[La(MACROPA)(H_2O)]^+$.

E -1941.223910549770 E_h

C	3.66511569451806	-1.19158121881960	-0.00417151649017
N	2.67242541455437	-0.96239892033641	1.07152745403839
O	2.05717996754127	-1.91134769237183	-1.61045470139020
C	3.26841332144591	-2.29870900177848	-0.93957995163781
C	2.86526684186259	-1.99729399393748	2.10995761973212
C	1.75534851983382	-2.04508961849279	3.12517250390686
O	0.54010443971241	-2.40084448936003	2.45411464023840
C	-0.56659681308775	-2.41916547873805	3.36314193738022
C	-1.78553976890025	-2.86946278658309	2.61755020364431
O	-2.04569947823879	-1.95474844630536	1.54001612363946
O	-0.54093586841930	-2.34246433270733	-2.47304208784549
C	0.54653084973232	-2.35926500683091	-3.40536205019814
C	1.78697436922738	-2.80766703630715	-2.69807309372117
N	-2.68928357340298	-0.93518784969633	-1.11070129551641
C	-2.87817278039465	-1.96670455755234	-2.15189826497510
C	-1.75789812115001	-2.00632118745396	-3.15403750859702
C	-3.27601190757753	-2.30453090682275	0.88344027200657
C	-3.66976834185901	-1.17424030471800	-0.02646492339344
C	2.93022157968754	0.36959129801427	1.64691393205584
C	-2.95419076156451	0.39991162633277	-1.67707042035509
C	-2.54908552131594	1.49178519585488	-0.72820216809871
C	2.53800355589347	1.47066052499146	0.70356601579879
C	-1.26354771859391	2.10596592269560	1.09376457697694
C	-1.73780016832313	3.40695453609800	1.06065191292996

C	-2.66026479766061	3.74587452132941	0.07577022802416
C	-3.08669781410426	2.77423771799031	-0.81715776455051
C	3.08468192504889	2.74769124631359	0.81561656533342
C	2.67226885098483	3.73722876820509	-0.06353131315870
C	1.75224590922044	3.42126574667726	-1.05789903184682
C	1.26843249000577	2.12474804937541	-1.11533497831541
H	3.98939746652402	0.47717521018223	1.91349709023937
H	2.34861430801575	0.47826943691412	2.56209593397908
H	-4.01610680812441	0.50887965889788	-1.93127498867891
H	-2.38237828389012	0.51267196869815	-2.59806006970351
H	-1.39714629950185	4.12477599904578	1.79343415443690
H	-3.05536774944786	4.75224446569972	0.01740878794608
H	-3.82737016118089	2.99541819710636	-1.57469225527511
H	3.82171167866737	2.95025046066084	1.58188206069932
H	3.07493409357641	4.73929478218737	0.01341177038500
H	1.41958052606435	4.15334415627463	-1.78008367590362
N	1.63514162079796	1.17937433124709	-0.23656946478536
N	-1.64198131252987	1.18017459612711	0.20039996600709
C	-0.31443297495150	1.60512181238489	2.15289095568750
O	-0.00880669712758	0.36989083990948	2.06647866934133
O	0.08929578153193	2.38929397522560	3.03410212514316
C	0.31651030452201	1.65726169388567	-2.18682344026749
O	-0.00058095568844	0.42227059699892	-2.13106302177644
O	-0.08026775753219	2.46486147400320	-3.04892240238670
H	4.64658235581915	-1.41986939043349	0.43031337134721
H	4.05571664893364	-2.43681254049122	-1.68572708215135
H	3.10295964690998	-3.25285974417250	-0.43042485378183
H	-4.65897965736521	-1.38453600495354	-0.45263850751380
H	-3.75247540340881	-0.26527771668703	0.56831013640264
H	-4.05401863565519	-2.43720399248424	1.64031752525786
H	-3.14291387216276	-3.25377571035860	0.35669110175711
H	-1.62504522935851	-1.05218442559513	-3.67145842886240
H	-1.97058702310458	-2.77633855809041	-3.90184919374575
H	-2.93908734389870	-2.93652772432961	-1.65870998734905
H	-3.82522892837662	-1.80356173960155	-2.68285079803623
H	1.98532064472089	-2.80738549274951	3.87555205814067
H	1.61030529677770	-1.08963929080863	3.63558520102437
H	3.81654600328148	-1.83509071740944	2.63382394631826
H	2.92376200326315	-2.96586427136290	1.61306962966256
H	1.68122549760623	-3.82754937608793	-2.31425856212632
H	2.62905556875949	-2.78760275384676	-3.39602762522270
H	0.67415428708173	-1.35840669361489	-3.82674168815523
H	0.32266417224774	-3.06116361445884	-4.21462070889875
H	3.76565643125478	-0.27304114671474	-0.58136742538204
H	-2.64253219841151	-2.87282632442834	3.29701569205182
H	-1.65106257207010	-3.87655848112585	2.21139674630829
H	-0.36192077386448	-3.12255386390349	4.17602131260139
H	-0.70284873259125	-1.41796077417978	3.77960294619523
O	0.10743723017666	-3.60149116454225	0.11856504786054
H	0.48047530141404	-3.67627929451003	1.01555591474833

H	0.70021243239469	-4.09602741987569	-0.46113088490507
La	-0.00708905477517	-0.95447141369870	-0.05712587424873

Table S31: Cartesian coordinates (Å) obtained from geometry optimizations for $[Ac(MACROPA)(H_2O)]^+$.

E -2285.140832160850 E_h

C	3.70534598073314	-1.20674686774975	-0.01845863781950
N	2.72285024507002	-0.95965317988759	1.06259420432917
O	2.08505252095291	-1.93859192367982	-1.61192959255913
C	3.30003667800874	-2.32142456766550	-0.94488590109397
C	2.89090359653050	-1.99526380164589	2.10450257704216
C	1.76955976154441	-2.02417655174134	3.10899012225926
O	0.55070548995071	-2.36636250102282	2.43240558913562
C	-0.54645306798746	-2.40692167197882	3.35629791773286
C	-1.77368765036761	-2.87445177899909	2.63379149665461
O	-2.07625817767598	-1.95069164569341	1.57614723255510
O	-0.53363926111980	-2.33467336409167	-2.46542334805897
C	0.55631431264861	-2.36670102777650	-3.39719684561801
C	1.79294264597112	-2.83623506105834	-2.69267268298620
N	-2.71925239560734	-0.94984740910960	-1.09430322306581
C	-2.88069866698364	-1.98032880605982	-2.14204735795053
C	-1.75514809230221	-1.99919131154572	-3.14213916705105
C	-3.29060997653962	-2.32846229732825	0.90536149975397
C	-3.69871673947870	-1.20738170908110	-0.01259677964170
C	2.99635021316750	0.37063294276626	1.63442681580385
C	-2.99782941272388	0.38345507726662	-1.65703370064807
C	-2.60415169616008	1.48847057904780	-0.71638582989779
C	2.60431236750210	1.48292103496730	0.70178980868101
C	-1.30913817305933	2.15529425911823	1.08082291459632
C	-1.79209268580261	3.45238867799875	1.01737140340117
C	-2.72703922067680	3.76005059813832	0.03443138074831
C	-3.15184854331457	2.76462972642584	-0.83232813790531
C	3.15390562669787	2.75715364473037	0.83134064436518
C	2.73175322293710	3.76243253247272	-0.02512431247936
C	1.79669129813598	3.46659050895702	-1.01142888981451
C	1.31187394670680	2.17093624169260	-1.08904072773842
H	4.05791137041546	0.46897338564257	1.89530400745121
H	2.42311269172912	0.48208093767794	2.55507791213117
H	-4.06038787497092	0.48119456416634	-1.91357701725676
H	-2.42791299857448	0.50214865031019	-2.57883019659356
H	-1.44708864707017	4.19217243910968	1.72577548743148
H	-3.12936216458533	4.76210256777983	-0.04507471459190
H	-3.89626211986145	2.96339461804473	-1.59246432384383
H	3.89793463327191	2.94662389326363	1.59425255157110
H	3.13592304394628	4.76279079216119	0.06502906834918
H	1.45282344999514	4.21428311287185	-1.71205962101791
N	1.69398682405962	1.20806579097538	-0.23577004235316
N	-1.69324292084453	1.20291549327860	0.21714630055653
C	-0.33305372734688	1.70180612649371	2.14013731188504

O	-0.01319081809642	0.46805670999577	2.09837654171386
O	0.08038281060078	2.52807463306298	2.97834925251379
C	0.33487727755702	1.73258343928236	-2.15373743682902
O	0.01584042515863	0.49797520320516	-2.12957908851085
O	-0.08044875249313	2.57043803983619	-2.97906967102984
H	4.68869060168153	-1.43737970342179	0.41081190300064
H	4.08377225581978	-2.46497252943047	-1.69378659806550
H	3.13760909397284	-3.27154144962593	-0.42762293324538
H	-4.68222302099850	-1.43710909156682	-0.44189826799590
H	-3.80039521966193	-0.29779480844660	0.57850170673122
H	-4.07457332536010	-2.47889158942014	1.65280566986269
H	-3.12850295047141	-3.27424561482616	0.38031036053891
H	-1.62827412235309	-1.03908941901693	-3.64987138909647
H	-1.96157730983641	-2.76315411575655	-3.89754366769480
H	-2.92809277284459	-2.95317404590241	-1.65284353904358
H	-3.82665933986091	-1.83264815191382	-2.67962700078923
H	1.98284448203977	-2.78861684146429	3.86190960460667
H	1.63553050044457	-1.06624892324418	3.61848029918757
H	3.83879245104483	-1.84741750937223	2.63869462817374
H	2.93892894335418	-2.96571563752715	1.61008352723373
H	1.67327078595243	-3.85094858292661	-2.30071825576338
H	2.62947104493119	-2.83285226689989	-3.39768222499971
H	0.69922228746040	-1.36685755140300	-3.81640567082204
H	0.32087569192405	-3.06333968752472	-4.20736717081385
H	3.80605691274665	-0.29293375430847	-0.60326863654749
H	-2.61353360047217	-2.90305617139399	3.33415835067410
H	-1.62873134941170	-3.87300185832388	2.21181005663691
H	-0.31612596193586	-3.11039236091459	4.16196736134807
H	-0.69296042226245	-1.41038629862853	3.78159527602950
Ac	-0.00046881978652	-0.97143394160423	-0.02871097069066
O	-0.00921791671118	-3.69876818448126	0.09408226235075
H	0.46564469699128	-3.93508675046356	0.90464702962660
H	0.34750090395476	-4.25986756481661	-0.60585238473993

Table S32: Cartesian coordinates (Å) obtained from geometry optimizations for $[La(BP15C5)]^{+}$.

E -1710.816145708486 E_h

La	0.09673240682858	-0.25309022708851	0.03993015578124
N	2.56964723040975	-1.57923832563696	-0.19806010065861
N	-1.57942699226087	-0.16085946725759	2.17790165818790
O	1.19215952133513	-0.94700188849169	2.26939519904576
O	-2.03515528477942	-1.83988035669448	-0.06494172766552
O	0.14430799983336	-2.59187299505655	-1.18760617540791
C	-2.35543715858848	-1.42094728617731	2.26321074907887
C	-3.03453954690401	-1.79376810158440	0.96681885625728
C	-2.16991291228881	-2.95512686926975	-0.95840197873049
C	-1.04315981554502	-2.85805301904654	-1.95400277163012
C	1.36795240187924	-3.09220248295378	-1.74742670367179
H	1.62241005429500	-2.52390851799912	-2.64605683262459

H	1.23568219201068	-4.14156401023651	-2.02049859527522
C	2.40413644439487	-2.97702759779580	-0.65654875621932
H	2.07489831646152	-3.57604090739043	0.19186507425458
H	3.36028866616918	-3.38351302808608	-1.00712825141398
C	3.22008751785709	-1.55050591354659	1.13417431028416
H	3.60528688225957	-0.54449358509201	1.30040070039154
H	4.07135814817002	-2.24149577179060	1.16105778588758
C	3.40857543146070	-0.85377934385053	-1.17251593005679
H	2.99523690354670	-1.01935400293948	-2.17024725967078
H	4.43106938276819	-1.24829257716843	-1.17582957129142
C	2.27809022806135	-1.89823620270763	2.26271286344693
H	1.86068830871714	-2.90450091434203	2.17580972637126
H	2.81685079214194	-1.82300720090153	3.21014781516111
C	0.38989203954872	-1.03420341070492	3.46420159279459
H	1.01809955330849	-0.80240086097427	4.32790262022591
H	0.02437104741502	-2.05820275305641	3.56786608579734
C	-0.71997660128390	-0.01199510245642	3.38180758750676
H	-0.28127099614506	0.98488678736854	3.33999760297897
H	-1.32032057151724	-0.08245183233157	4.29681304569057
C	3.43562760694290	0.62329061896760	-0.90528594198107
C	4.54362585586571	1.40107117975091	-1.23247922922860
H	5.41641057919779	0.93447924884839	-1.66999386216498
C	4.50597467677626	2.76376584964461	-0.97925271212541
H	5.35316531538893	3.39019202073937	-1.22831260793307
C	3.37703683006540	3.31127584418203	-0.37894066965296
H	3.31415236398680	4.36336098548892	-0.13989319143299
C	2.32084249268563	2.46961080861616	-0.06896029018867
C	1.08360354154416	2.95757478853750	0.64459767747404
N	2.34415728427990	1.15669142091613	-0.34792813580002
C	-2.48843333627528	1.00674713396465	2.09980306355883
H	-1.91898332987294	1.88491341319061	2.40952572165844
H	-3.32246045244382	0.89008711988084	2.79909348276260
C	-3.01142696117684	1.27498706963116	0.71427385655683
C	-4.27321118408033	1.82926328072956	0.50581884388692
H	-4.92164666902596	2.02394216558898	1.34994789737169
C	-4.67666804735293	2.11092083696187	-0.79068899404667
H	-5.65382261574932	2.53809621420359	-0.97763576749815
C	-3.81738330964697	1.83133432796841	-1.84872631721222
H	-4.09604274997385	2.03097323704867	-2.87369147861619
C	-2.57790465426492	1.28059868701152	-1.56169633014256
C	-1.57192027399005	0.94788508093452	-2.64028578779866
N	-2.19055876271906	1.01536074445947	-0.30632596738893
O	-0.45052566061057	0.47216653932594	-2.23137604397304
O	-1.86887857049076	1.14772865473270	-3.82970109976051
O	0.97064862632504	4.16999507128506	0.90345606545357
O	0.23308158214447	2.05397862159338	0.94915843379004
H	-1.66633067791544	-2.22399858600500	2.52023731256025
H	-3.10617403048688	-1.34578328370954	3.05906568650249
H	-3.81793989740892	-1.09346984917250	0.67118783432577
H	-3.48192216763588	-2.78254154792896	1.08197499924251

H	-0.93805850378737	-3.80229001544086	-2.49065614835709
H	-1.19878125330917	-2.04771552986781	-2.66989434737064
H	-2.10050640747795	-3.87752639352270	-0.37602127179414
H	-3.13522582906722	-2.91420099329576	-1.46908145550324

Table S33: Cartesian coordinates (Å) obtained from geometry optimizations for $[Ac(BP15C5)]^{+}$.

E -2054.726067877944 E_h

Ac	0.08694568937058	-0.18787935029063	-0.07724184767582
N	2.61628061745271	-1.57078874214713	-0.22489047897680
N	-1.60877288873167	-0.13292974656821	2.14008342900825
O	1.17577040475359	-0.93486554404387	2.22380329099266
O	-2.07415187509243	-1.84371544186242	-0.12008449630885
O	0.17229834388541	-2.64154056255783	-1.18506394762542
C	-2.37564493140481	-1.39893753523285	2.22078153600663
C	-3.06016956821908	-1.78129890434596	0.92790908400841
C	-2.13512777991131	-3.04312739325017	-0.90914643108359
C	-1.02054370634660	-2.97206929385431	-1.91906933229963
C	1.40635634779759	-3.09295986491537	-1.76955417347702
H	1.63012663360398	-2.50055449990592	-2.66087083239426
H	1.30216253050364	-4.14055729306303	-2.06039764906502
C	2.45475861569086	-2.96740987273616	-0.69097230227759
H	2.14103980784936	-3.57477980204758	0.15734039002611
H	3.40997486513101	-3.36438097691397	-1.05464838045045
C	3.22672778543556	-1.54865276045521	1.12602805503510
H	3.61064939007163	-0.54481640724509	1.30774949149181
H	4.07483622354702	-2.24239769855492	1.17652351191392
C	3.48649608572520	-0.84679973104172	-1.17386224126798
H	3.08108566082855	-0.98163658946402	-2.18003946568041
H	4.49779202239666	-1.26950725762285	-1.17266264616468
C	2.25336019132947	-1.89652352780606	2.22767812977658
H	1.82800345470117	-2.89766483843082	2.12180679103976
H	2.77235287987370	-1.83694753134925	3.18727961117180
C	0.36957444084782	-1.01569047273654	3.41598759524692
H	0.99691108043077	-0.78564870342965	4.28093821150263
H	-0.00112249578673	-2.03760959668546	3.52167750815178
C	-0.73497683076257	0.01188522563102	3.33400728039912
H	-0.28956122264823	1.00639698591359	3.28577601919543
H	-1.32649640836261	-0.04920338824538	4.25573382976334
C	3.55627236114759	0.62458132673028	-0.87504260231836
C	4.71897458125903	1.35593993382146	-1.10576587731079
H	5.59889323785594	0.85706191137377	-1.49030078513766
C	4.72674115366699	2.71425714902021	-0.82408623800516
H	5.61817889707981	3.30401987919470	-0.99646960366390
C	3.58320324058745	3.30355336018355	-0.29537340784071
H	3.55261943586333	4.35296896829932	-0.03890904587409
C	2.46659412204067	2.50896436606035	-0.08343030442605
C	1.20010060808040	3.05561594673796	0.53495984330648
N	2.45092954236742	1.19982295681596	-0.38610370043361

C	-2.51936420511656	1.03476478965688	2.09215680740590
H	-1.93503602566791	1.91419037862111	2.37247859068460
H	-3.32144949341844	0.92489728032606	2.82941153824842
C	-3.10687944614933	1.29591993623042	0.73083518770281
C	-4.39082180692771	1.81811664674411	0.58097370861076
H	-5.00504866228250	1.99486507541741	1.45421002395628
C	-4.86079356190964	2.08951670152188	-0.69558651735980
H	-5.85596041992213	2.49176471521137	-0.83750105746788
C	-4.04428418768238	1.82899279320509	-1.79196564932362
H	-4.37575353211949	2.01869797591986	-2.80307256508067
C	-2.77882241651179	1.30858107441199	-1.56400088289332
C	-1.82527657745614	0.99210802073983	-2.69576424154014
N	-2.32472375696608	1.05690888418234	-0.32630613755815
O	-0.68493937858484	0.51019529891695	-2.35127342640256
O	-2.18240215156323	1.20877948622427	-3.86560189487947
O	1.14435980352204	4.26325178370309	0.82975445862400
O	0.26231928678518	2.20549864973562	0.71955049033423
H	-1.67973999015212	-2.19722205126193	2.47444316130668
H	-3.12494844487923	-1.33306896497032	3.01942912611300
H	-3.84189657157145	-1.08164070852451	0.62956923995500
H	-3.51276453332787	-2.76546321657967	1.05783393293026
H	-0.90072277170079	-3.94242903537299	-2.40432121108918
H	-1.20494211254710	-2.20781623459393	-2.67844725050204
H	-2.00677882880063	-3.90392486921964	-0.24800253337488
H	-3.10062975895876	-3.10902209322499	-1.41681871667916

Table S34: Cartesian coordinates (Å) obtained from geometry optimizations for $[La(BP12C4)(H_2O)_2]^+$.

E -1709.850657950308 E_h

La	-0.04573794725956	-0.13157553573661	-0.00084234732711
N	-1.79277370659951	1.11397217916364	-1.63941764262016
C	-0.95512268368953	1.62674187414826	-2.74632373259122
C	0.10921630850861	2.60228818823086	-2.28712572796522
C	1.43129439566816	3.10322572512387	-0.36575981573048
C	2.18115522724246	2.51862142106906	0.81536921961443
N	1.46962524926932	1.46482672332454	1.58665410505384
C	0.56490784800282	1.98812562618974	2.63373939932018
C	-0.59486558981365	2.77988762731680	2.07363843337569
C	-1.95267675855165	2.93148656206185	0.13230066078225
C	-2.62680585453012	2.14944600089217	-0.97509746015158
C	-2.70211614469907	0.06169779458969	-2.15121454813266
C	2.48174644430670	0.59697237689030	2.23284746697898
O	-0.82008600631766	-2.52255878223487	-1.33673717407449
H	-1.56835898502838	2.13018261254408	-3.50350327572324
H	-0.48560114556149	0.76169993006412	-3.21678220631441
H	0.66293377655098	3.81467547175909	-0.06328607482994
H	2.15075876042521	3.63814706016338	-0.99164383678129
H	3.10081568695512	2.07340269063035	0.44229698068858
H	2.46404379952598	3.34455223939743	1.47782192724207

H	1.10889848153858	2.63247252231579	3.33513385637556
H	0.19200392355957	1.12606385587005	3.18697982238615
H	-1.29048503027241	3.71012675793822	-0.24808374492737
H	-2.72954824569831	3.41971467652215	0.72729233322156
H	-3.48719739700000	1.63779245417433	-0.54930037793318
H	-3.00538191042574	2.86120176613773	-1.71714209453447
H	-3.46750943286189	0.48334711633282	-2.81080605840723
H	-2.10942315930298	-0.64279260903627	-2.74023404835736
H	3.15169123701035	1.17656796429022	2.87655239948326
H	1.95328327190299	-0.11958970968463	2.86813673067019
H	-0.39948360064794	-3.08271728028186	-0.66525789983934
H	-0.09839016080218	-2.32245421482538	-1.96127725370735
O	0.81347410738585	2.06275468084308	-1.14656799067037
O	-1.19380681672244	2.05262211753443	0.98095559711403
C	-3.34713220807121	-0.66549429203752	-0.99919620515644
C	-4.61928373784466	-1.22232030993195	-1.07090842298370
C	-5.12261648335593	-1.88137044268834	0.04380986485591
H	-5.20049698388596	-1.13177896895734	-1.97918346975657
C	-3.09443735025379	-1.37694099299795	1.19256056951812
C	-4.35488343174977	-1.95752797653192	1.20114886478935
H	-6.11058196525533	-2.32323909204942	0.01436908134678
H	-4.71850284839498	-2.45239298481781	2.09060708075140
C	3.27426679220499	-0.14641747721171	1.19202649372926
C	4.60148698479149	-0.52114402952959	1.37483385502455
C	3.24249239060855	-1.06846178647603	-0.93146458015974
C	5.24916515664430	-1.19981724993127	0.35109443151664
H	5.11203234189751	-0.27439149000268	2.29645063075204
C	4.56561859549181	-1.47251955726789	-0.82937664025622
H	6.28249209523049	-1.50185982599623	0.46587129415933
H	5.04143941211608	-1.98354463755707	-1.65435386330460
N	2.61844499296546	-0.42765751072203	0.06361747930167
N	-2.61287073869396	-0.75317221054746	0.11293476346123
C	-2.16669686202005	-1.40512325643602	2.38630724409464
O	-1.00294464846305	-0.90238393597657	2.19758040634817
O	-2.56899863575497	-1.90218071901736	3.45320301862170
C	2.40446913254823	-1.31393454881743	-2.16335503801309
O	2.92343698479850	-1.86155543762121	-3.15138380054946
O	1.18470679980695	-0.92632036229928	-2.08754244721687
H	-1.34271208302026	2.93550269356086	2.85509690329878
H	-0.27293857559345	3.75805183786675	1.71157311411150
H	-0.32340631622761	3.56433413013910	-2.00834521828937
H	0.82006528357258	2.77127320462021	-3.09934549307362
O	0.78163192633510	-2.56582223792116	0.96778706594560
H	0.43058223220310	-2.50308805431364	1.86896974036206
H	1.72247780530161	-2.76351736224855	1.06184265508262

Table S35: Cartesian coordinates (Å) obtained from geometry optimizations for $[Ac(BP12C4)(H_2O)_2]^+$.

E -2053.761910009146 E_h

Ac	-0.04198600969711	-0.21532877128354	0.01232932297996
N	-1.84238990162881	1.13436451129833	-1.61639936454255
C	-1.00335482834644	1.64774192076528	-2.72188871874642
C	0.07036030247833	2.61910041019064	-2.27659050427482
C	1.43439008727628	3.11181704717007	-0.38716890766715
C	2.21088558491939	2.53237607874948	0.78047779161208
N	1.52487731347781	1.47761315675784	1.56946487572879
C	0.62047125481943	1.98826332742154	2.62281176430247
C	-0.54735343455235	2.78398340397844	2.08447308374931
C	-1.94917276987564	2.93884901821174	0.17497240385115
C	-2.65261000362906	2.16533209034184	-0.92134482535189
C	-2.76931232524595	0.10978751560061	-2.14913848633963
C	2.55427836245629	0.62739543148892	2.21018061755439
O	-0.81200005003445	-2.54764252931049	-1.36554533635040
H	-1.61503909731166	2.15350702254406	-3.47939353520948
H	-0.53974421647847	0.78061656057246	-3.19685907566300
H	0.67125612587913	3.82176283749918	-0.06810359810828
H	2.14075732830778	3.65021845234610	-1.02531820421907
H	3.12460074935354	2.09006186845304	0.38912572384058
H	2.50520410646866	3.36364730027591	1.43190389281083
H	1.16317231061106	2.62677353332073	3.33109609946989
H	0.25369666151712	1.11981292052817	3.17302408682693
H	-1.28848790319758	3.71153244348646	-0.21918244072126
H	-2.71161571539442	3.43489011963505	0.78236115271839
H	-3.50178694891003	1.65048705798304	-0.47650902115205
H	-3.05171043534043	2.88694842593181	-1.64370848755624
H	-3.52711700810685	0.55827446815627	-2.80039815610433
H	-2.18920326542506	-0.58959274605562	-2.75692299182141
H	3.22123571301033	1.21900444534473	2.84657542607227
H	2.04238890561759	-0.09293673900471	2.85546370166380
H	-0.62395708745426	-3.31191002929132	-0.80378707051225
H	-0.03505199354061	-2.46518198504583	-1.94819930295707
O	0.80227122003850	2.07340143436961	-1.15810255302679
O	-1.18078036528885	2.05722943413517	1.01196779536242
C	-3.43216791245423	-0.64176456099820	-1.02308154310520
C	-4.71693900394982	-1.16556667431632	-1.12319853430343
C	-5.24267648404802	-1.85468664384333	-0.03785959685349
H	-5.29003555138648	-1.02610973372968	-2.03048228901746
C	-3.21059932746499	-1.44064799336014	1.14095694547319
C	-4.48370479393144	-1.99256471784705	1.11955011801074
H	-6.24063674182132	-2.27146690657984	-0.08908509593245
H	-4.86431452455935	-2.51194829672103	1.98766416489746
C	3.35489013106473	-0.11406285136332	1.17277508376308
C	4.69304828451932	-0.44822320225570	1.35473752489752
C	3.33332268376555	-1.08961370965046	-0.92720113428970
C	5.35148553214025	-1.13644322812464	0.34440782138181

H	5.20345192798978	-0.16361562975740	2.26548078675907
C	4.66696977310789	-1.45787465582151	-0.82290830884957
H	6.39320681041776	-1.40817141592015	0.45927510804371
H	5.15020958722981	-1.97849701949882	-1.63745347718709
N	2.69829308312780	-0.43990394875896	0.05614668795113
N	-2.70586514691722	-0.78731704997112	0.08809745648116
C	-2.29802590183516	-1.53376393539872	2.34406488965231
O	-1.13306246700892	-1.02051939630890	2.20088506151694
O	-2.71595028096039	-2.08954591373091	3.37622460614054
C	2.50088469103978	-1.38687822327183	-2.15343234300213
O	3.03795893164301	-1.94874372234069	-3.12468661091072
O	1.27263839543129	-1.02692377107142	-2.09302026522961
H	-1.27202641096706	2.94896162676548	2.88586948504056
H	-0.22683296297489	3.75852167055108	1.71166511326021
H	-0.35611439033475	3.57962831729948	-1.98354615000192
H	0.75996845020515	2.79364252061279	-3.10617640117999
O	0.90247435555616	-2.55095382784619	1.17141863401445
H	0.47325686386411	-2.49193901428255	2.03902752377062
H	1.84650373273851	-2.64909452902502	1.35276258058908

Table S36: Cartesian coordinates (Å) obtained from geometry optimizations for [La(NO3PA)].

E -1857.889894556449 E_h

La	0.00127532072861	-0.00351346000557	-0.40000518602714
C	-3.37052465861664	-0.67951637324074	-1.14188274830649
C	-4.75835530383161	-0.66006674819378	-1.14193271619714
H	-5.30691082288288	-1.34722317936943	-1.77032944017321
C	-5.40783609788886	0.25336793800868	-0.32010417080120
H	-6.48945169181243	0.29305987373474	-0.29359523846996
C	-4.65618307299767	1.10813086217884	0.47603298279026
H	-5.12971696469839	1.82221251082825	1.13662321895244
C	-3.26917077888759	1.02716826348380	0.42258489780574
C	-2.36723811248824	1.91641248516272	1.23124282271282
H	-1.95469439583432	2.69035270637589	0.57688599928326
H	-2.93761493132752	2.41546739691089	2.02181184128830
C	1.07947067486338	3.25628517014501	-1.14222982255788
C	1.77254165002423	4.45874502950633	-1.12638610359047
H	1.44154375221385	5.27874295168158	-1.74782395559754
C	2.88256560060566	4.57217719412832	-0.29780457852815
H	3.44348679265063	5.49737481179578	-0.25778057652294
C	3.26085379804278	3.48995314810089	0.48697578529491
H	4.11311153387500	3.54804700264766	1.15095668204496
C	2.51509193838653	2.31878447596378	0.41747630951432
C	2.84762580722595	1.08771480560503	1.21346109432998
H	3.30338739180357	0.34807308755923	0.54804830595771
H	3.57473403173931	1.32812062138090	1.99615676750561
C	2.28811381030054	-2.56870772780628	-1.14857954265985
C	2.98116099176731	-3.77126294665908	-1.13968885534831
H	3.85538355081968	-3.89360012808622	-1.76326759360730

C	2.52426077258661	-4.79265720522509	-0.31528155593293
H	3.04369428204147	-5.74199754037069	-0.28130111753798
C	1.40065443045459	-4.58071677149429	0.47367035810713
H	1.02539997848110	-5.35004633500088	1.13539156670423
C	0.76125978296144	-3.34775586261547	0.41141230335870
C	-0.46743504538966	-3.02134380659123	1.21309197602577
H	-1.33939799292751	-3.04901756866000	0.55239437071421
H	-0.61761579454534	-3.77077690896949	1.99739202368937
C	-0.25031654535808	2.08556166545335	2.36612101534799
H	-0.67328850517063	2.61400272820156	3.23056702867701
H	-0.03596883784927	2.83218589925518	1.60208533158653
C	1.03227593067254	1.39708572655380	2.81052465855047
H	1.75525998360427	2.17168347363266	3.07946007572708
H	0.85560078460518	0.82251498945960	3.71852964815080
C	1.94761441817304	-0.83439957451940	2.35574230153405
H	2.62072503507118	-0.73264946853766	3.21709705685663
H	2.48342962116549	-1.39157012623452	1.58791401101367
C	0.71190389461073	-1.60177895853804	2.80488002133310
H	1.02274246516677	-2.61487533938330	3.07348406438623
H	0.30458076815172	-1.16127375359895	3.71363120847426
C	-1.68051975106905	-1.27958279593084	2.35463659833552
H	-1.93054833259892	-1.91566696876739	3.21393867030915
H	-2.43111042078941	-1.46143335012132	1.58599488565283
C	-1.72516321298520	0.17252308438382	2.80948041258495
H	-2.75709032642428	0.40881682197136	3.08226551766301
H	-1.13763538023729	0.29911709324572	3.71753770960874
N	-2.65112709668777	0.15001239693644	-0.37642986186372
N	1.44944685611428	2.21654142849080	-0.38530788297624
N	1.20377621581642	-2.37146951448538	-0.38926765522565
N	-1.23740009579616	1.14604923006058	1.79053489401312
N	1.62254401676264	0.49066670233094	1.78443464803474
N	-0.37118749449461	-1.66135912782542	1.78196088280500
C	2.70099681287814	-1.39863479246731	-2.01504751264988
O	3.73411666587667	-1.49893839434087	-2.70378941652661
O	1.93314768034417	-0.37812729704917	-1.97181869605354
C	-2.57154996499817	-1.63149683799875	-2.00487934354971
O	-3.18192795172387	-2.46815919160698	-2.69720091205107
O	-1.30187509579977	-1.49516669862080	-1.95507302507921
C	-0.13622200121584	3.03083785652141	-2.01471371751316
O	-0.64370840880160	1.85876080851749	-1.96990520464750
O	-0.55589895445688	3.97451451210132	-2.71132351672988

Table S37: : Cartesian coordinates (Å) obtained from geometry optimizations for [Ac(NO3PA)].

E -2201.798304272148 E_h

Ac	-0.00935827989327	0.00780824238327	-0.51676489205181
C	-3.47845435141438	-0.67012740281997	-1.15195836380915
C	-4.86417415234978	-0.69099202082094	-1.06757770046525
H	-5.43088934062986	-1.38688975537128	-1.66954447586875

C	-5.48791925812612	0.18980177177767	-0.19231622423940
H	-6.56645854715750	0.19524426878094	-0.09786303616329
C	-4.71353143646254	1.05331481490250	0.57242420337916
H	-5.16580668818623	1.73926190017414	1.27630812331149
C	-3.33095546339818	1.01598591267987	0.43154802878020
C	-2.40688489361872	1.90812727524215	1.21307217770206
H	-2.00311057383394	2.67531921735485	0.54520858133437
H	-2.95974829132254	2.41671545680608	2.01012265982184
C	1.16880003232836	3.33816269547956	-1.15489623753229
C	1.86412417965623	4.53773967146844	-1.08660603457608
H	1.55405488880943	5.37504201773387	-1.69559007603456
C	2.94657991346084	4.62717919986523	-0.21982409586312
H	3.50750022015860	5.54963186895054	-0.13812579666000
C	3.29432875678950	3.52523621922030	0.55142394576713
H	4.12145040609333	3.56564195498695	1.24775828177171
C	2.55080548256755	2.35702131946674	0.42548046029064
C	2.85574096684463	1.10914259759110	1.20693214009850
H	3.31523319870542	0.37528764422585	0.53715609633231
H	3.57519516730938	1.33086795298761	2.00246216461241
C	2.32090714395835	-2.64483070539591	-1.16420673276363
C	3.03696683663656	-3.83173930979971	-1.08567070696873
H	3.92425986521253	-3.96617751775278	-1.68780275612242
C	2.58844074113719	-4.81969630426128	-0.21750482499504
H	3.12667755576471	-5.75484393597686	-0.12780586751026
C	1.45033441073188	-4.59050470222882	0.54564584803302
H	1.08325811757085	-5.33128841891394	1.24354960463386
C	0.78611961804396	-3.37656245196743	0.41077995575824
C	-0.45422989721995	-3.03531206654181	1.18952558258694
H	-1.31684147312545	-3.06693579123910	0.51672710515515
H	-0.61838004806949	-3.77772679754468	1.97796832239555
C	-0.27833482722549	2.08275647048009	2.32109830888389
H	-0.69976607954450	2.62414066914394	3.17858604488818
H	-0.06601543904798	2.81968051060053	1.54620823352620
C	1.01016507744362	1.40952404838197	2.77463561406749
H	1.71873254254962	2.19689628006245	3.04537718817360
H	0.83700003544505	0.83634731873349	3.68433651416697
C	1.94370765183979	-0.81751606836279	2.31993792805174
H	2.62116827099433	-0.71963675312121	3.17872596397690
H	2.47808493518151	-1.37129855328053	1.54799681393165
C	0.71442314332416	-1.59341409969631	2.77270023536247
H	1.03740854863130	-2.60102482332361	3.04837440196077
H	0.30310114455557	-1.15171712348527	3.67905980818619
C	-1.67982162189270	-1.29319050490254	2.31233524094083
H	-1.93678930710883	-1.93470572349177	3.16588934326658
H	-2.42386390565576	-1.47451232316156	1.53637211322315
C	-1.73984774905218	0.15650557471265	2.77525429254898
H	-2.77447277715186	0.37620286051923	3.05206166520005
H	-1.15289840234434	0.28637031365764	3.68324863469906
N	-2.73763151573113	0.17251374680821	-0.42149687130119
N	1.51516975164148	2.27540305746133	-0.41786707256268

N	1.21822832481558	-2.43346796138862	-0.43490699677552
N	-1.26907840895243	1.13846039321092	1.75906211226226
N	1.62027021935523	0.51095278799027	1.75502303636765
N	-0.36499942668668	-1.67079867883968	1.74971958157498
C	2.74208539306896	-1.50600067847909	-2.06906120972803
O	3.77455317855655	-1.63799850361388	-2.75316702352749
O	1.98662005233334	-0.47461881950386	-2.05703986618594
C	-2.70973868128219	-1.60540235707105	-2.06138629170879
O	-3.34599355871254	-2.42218947549676	-2.75394542468990
O	-1.43714271110314	-1.48205899133358	-2.04390582443555
C	-0.03193901169390	3.15230461995847	-2.05770781883938
O	-0.57652432712493	1.99563957775304	-2.03211491303563
O	-0.41080232639685	4.11322838763470	-2.75375922261032

Table S38: Cartesian coordinates (Å) obtained from geometry optimizations for $[La(PYTA)]^-$.

E -1972.258890881035 E_h

La	0.00027059403430	-0.00058522685377	0.00020637271319
O	2.17229379941369	0.86243293449136	1.14320219307668
N	0.89136477367265	-1.21318382840730	2.26573007584815
N	0.00092868287791	-2.65633206866306	0.00013087431984
C	0.16153746955033	-3.32637530776706	1.15356297248673
C	2.96020988713168	-0.01751343892603	1.62197228661430
C	2.33990177197649	-1.34878152107385	2.05245149518573
C	0.67050080064608	-0.35201987985220	3.45470813301578
C	0.25301527451489	-2.52909922209177	2.43355208643800
C	0.18351619967879	-4.71637190312012	1.18571819119378
C	-0.00203433403434	-5.41680703347042	0.00061296642684
O	-2.17257813784701	-0.86211872120736	1.14272309794568
N	-0.89098319427572	1.21281978961425	2.26550976249157
N	-0.00077096548208	2.65576988228914	0.00007544694475
C	-0.16106138703952	3.32584326281157	1.15352643042454
C	-2.96025362180013	0.01831189213323	1.62104034846096
C	-2.33939325893116	1.34915641709850	2.05197883493055
C	-0.67068420410093	0.35151011889088	3.45445761182827
C	-0.25229174558033	2.52853821334531	2.43349929904056
C	-0.18331754713818	4.71583900519245	1.18572267270271
C	0.00154287383054	5.41634603979962	0.00055786020075
O	-2.17236313299543	0.86116127158851	-1.14324807183683
N	-0.89062700208163	-1.21354371154868	-2.26561611833488
C	-0.16123733400437	-3.32647917333885	-1.15311740171880
C	-2.95991960925478	-0.01877718941067	-1.62255674308251
C	-2.33913239146146	-1.34992247087857	-2.05274657438394
C	-0.67022902678897	-0.35243823849924	-3.45471226364611
C	-0.25140228695374	-2.52912444956723	-2.43313354003914
C	-0.18619497256300	-4.71637818006641	-1.18477067989170
O	2.17341655783896	-0.86017855801816	-1.14321364421141
N	0.89046133049866	1.21345075297757	-2.26568867914702
C	0.16072288093597	3.32601569723454	-1.15319721236188

C	2.96056296754190	0.02026485141458	-1.62230025574078
C	2.33889643233214	1.35075902224834	-2.05305729012308
C	0.67040597611742	0.35207115558811	-3.45462340893105
C	0.25070604055776	2.52872882437758	-2.43324903885761
C	0.18528962168775	4.71592674253993	-1.18488875223304
H	0.33705688385659	5.23209786075640	-2.12395413661300
H	1.47111016574657	-0.38576163252164	-3.47643453032274
H	0.75635944139109	0.94642919082316	-4.37259956552642
H	-0.76668674357771	2.35928866725787	-2.79344631533043
H	0.76930062118624	3.12546310539971	-3.19310297676741
H	2.51811196970853	2.07540211709748	-1.25511288865120
H	2.84515757096455	1.71711659191621	-2.95376810221827
H	-0.33850948651582	-5.23255313142426	-2.12374562358552
H	0.76597504724934	-2.36001448887315	-2.79356777024387
H	-0.77033358560618	-3.12572671151486	-3.19285177522197
H	-1.47090482163497	0.38541058368982	-3.47692087230584
H	-0.75597719829954	-0.94702259528198	-4.37254933948752
H	-2.51866062112514	-2.07410519635076	-1.25445681866612
H	-2.84570786642427	-1.71634301646244	-2.95325179876539
H	0.00250929154645	6.49932869398896	0.00075513131913
H	-0.33427733874321	5.23192836610777	2.12496664284336
H	0.76477562401369	2.35993153238243	2.79502739058533
H	-0.77215491002664	3.12506351077251	3.19265344591162
H	-1.47087089975441	-0.38689351323266	3.47596345211137
H	-0.75727850415326	0.94571203713202	4.37246773547400
H	-2.51848074495030	2.07357002931282	1.25380842318791
H	-2.84644838589669	1.71534125136432	2.95231220315698
H	-0.00328871357543	-6.49979179651694	0.00081919940429
H	0.33475107557118	-5.23253854690665	2.12487396920524
H	-0.76404417946818	-2.36069072007855	2.79522581298727
H	0.77302927168669	-3.12563059280358	3.19259461231292
H	1.47066153978300	0.38639291852630	3.47667639880045
H	0.75673542563551	-0.94635485158093	4.37265953589324
H	2.51948996886445	-2.07295211282757	1.25415657723881
H	2.84692188018447	-1.71494835100654	2.95280819898159
O	-4.19706728628317	-0.11634565960857	1.75284717403969
O	-4.19651451905916	0.11653253372163	-1.75587756133543
O	4.19732198938443	-0.11411481990088	-1.75487510374461
O	4.19686825581619	0.11786299576789	1.75454279758296

Table S39: Cartesian coordinates (Å) obtained from geometry optimizations for [Ac(PYTA)]$^-$.

E -2316.164723838342 E_h

Ac	0.00000081207353	-0.00000142743583	0.00000628147562
O	2.23120159424358	0.81092260894492	1.20360640690281
N	0.87842195072311	-1.24833126401249	2.30368379330694
N	-0.00020329639041	-2.68445885300573	0.00000519878432
C	0.13920337229105	-3.34946500293096	1.15865146069586
C	2.98830251309465	-0.10656213689160	1.65930940356191

C	2.32449098937350	-1.41467636862840	2.09951489650189
C	0.66080605891905	-0.37445759180758	3.48496952080119
C	0.20670876622874	-2.55178962343863	2.44504734906191
C	0.16194614999276	-4.73895046206940	1.18995789665054
C	0.00027373569906	-5.43730911681987	-0.00006343910331
O	-2.23124626807376	-0.81096572708379	1.20356767480275
N	-0.87846216076080	1.24831035772303	2.30364098925790
N	0.00013184549055	2.68446453071555	-0.00001855441771
C	-0.13922889624599	3.34946456506216	1.15863057799690
C	-2.98835053808580	0.10652182465651	1.65926166227634
C	-2.32453431528023	1.41464539278280	2.09943844155651
C	-0.66086193907554	0.37445922025091	3.48494767152405
C	-0.20676459506960	2.55177937322694	2.44502158674279
C	-0.16174876900144	4.73895727650927	1.18996841172217
C	-0.00004251553920	5.43731426737285	-0.00004822763283
O	-2.23113707762073	0.81116157265904	-1.20354263100683
N	-0.87854110163847	-1.24822783488965	-2.30363604321835
C	-0.13937109641938	-3.34944241422937	-1.15866967504509
C	-2.98831984138079	-0.10627755555222	-1.65920481698322
C	-2.32462672035624	-1.41445264807599	-2.09941700546640
C	-0.66091188201896	-0.37439134379750	-3.48494749254435
C	-0.20687870896820	-2.55171532802120	-2.44503743787535
C	-0.16116053512387	-4.73894428447288	-1.19010872832306
O	2.23110027801556	-0.81116143265928	-1.20360827898184
N	0.87848910328125	1.24823619642622	-2.30367054494847
C	0.13937901947684	3.34945394184563	-1.15868320346071
C	2.98827694401446	0.10627853105865	-1.65928326136619
C	2.32457293720079	1.41445277171260	-2.09948342384185
C	0.66081382766377	0.37441099563249	-3.48497808927519
C	0.20686530766776	2.55174291022872	-2.44505574555631
C	0.16139759328849	4.73895194996403	-1.19009488948206
H	0.29555555425221	5.25715389926923	-2.13055558875386
H	1.48234493131478	-0.33985592444707	-3.51299627236103
H	0.71883320989146	0.96602619995078	-4.40721546009915
H	-0.81706653072779	2.36232953987729	-2.77556401232403
H	0.68964026557077	3.16359759822634	-3.21650274664680
H	2.49310099770393	2.15037573014838	-1.30873899736469
H	2.81983229224646	1.78743398618872	-3.00374878648292
H	-0.29537808616381	-5.25714509850906	-2.13056160175505
H	0.81705089370808	-2.36227825857711	-2.77554094088689
H	-0.68963062732241	-3.16357231425325	-3.21650020328963
H	-1.48244305404851	0.33987783281184	-3.51291314155476
H	-0.71898067597753	-0.96600084861360	-4.40718996170266
H	-2.49313482988488	-2.15037103538165	-1.30866526078423
H	-2.81989750604224	-1.78744231654174	-3.00367434485380
H	-0.00064935757871	6.52027339606347	-0.00013219929230
H	-0.29516252809132	5.25717218376926	2.13052814759399
H	0.81715674914892	2.36231066852580	2.77553231497206
H	-0.68950815217921	3.16367077333045	3.21646272732282
H	-1.48245703793359	-0.33974098108601	3.51294274235022

H	-0.71885443605472	0.96607818042993	4.40718530323722
H	-2.49298610183552	2.15055562921195	1.30866552936669
H	-2.81977092227794	1.78770042247545	3.00368726100508
H	0.00124007347739	-6.52027179895031	-0.00019419071684
H	0.29521845304482	-5.25716976839244	2.13053618801647
H	-0.81721829409398	-2.36230334689737	2.77551821644664
H	0.68941281912923	-3.16368968741567	3.21650872757805
H	1.48240157901175	0.33973896890603	3.51296585069911
H	0.71877786482735	-0.96604911124315	4.40722669804187
H	2.49295769912133	-2.15061449830369	1.30877022990970
H	2.81971749765347	-1.78770258455628	3.00377925226200
O	-4.23352716222823	0.03279174534641	1.76036876521013
O	-4.23349424644470	-0.03246208895218	-1.76026237585408
O	4.23344940160770	0.03246181774827	-1.76035358447224
O	4.23347672548635	-0.03282678110894	1.76041684008964

Table S40: Cartesian coordinates (Å) obtained from geometry optimizations for $[La(OCTAPA)(H_2O)_2]^-$.

E -1779.669582601773 E_h

La	6.22704072470897	22.27651928589030	15.04989851890581
O	4.86920625147981	22.36769988901274	10.60422344739671
O	5.82422883241792	21.89781963995700	12.57693673371361
O	3.07834219793752	23.16404175154989	18.28744841133813
O	4.79205086063079	22.27737126269517	17.14748516429192
O	10.41482355279849	23.88382448364009	14.03644932843680
O	8.19317928386820	23.60366708431210	14.11786977953104
O	5.84175248065306	17.66657956103812	15.33232794456407
O	5.46323748795489	19.85545965683329	15.02914449605285
O	3.54471291663273	22.11562147227202	14.56361197034361
H	3.19121113141069	21.51087452767626	15.23021239416123
H	3.38170043542626	21.67457856030564	13.71905492551651
N	5.31710030424494	24.34325780882212	13.51612028464315
C	4.89288668898768	24.06021748318052	12.27549764680604
C	4.22336160010372	24.99118359449631	11.49231088511872
H	3.89726104596069	24.72311627370805	10.49740568984063
C	3.98554004632465	26.25511640638697	12.02057191236088
H	3.45336441243118	27.00036447897761	11.44275548058547
C	4.44268886124369	26.55115849537112	13.29704187658895
H	4.28307634517394	27.52625350375580	13.73875599244540
C	5.11854850638311	25.56783617262186	14.01633722448528
N	5.63276132542460	24.70808043382204	16.27827288719673
N	8.20486933448139	23.18248083524359	16.79702582997980
N	7.30007476171907	20.52742765772708	16.84044828226389
C	8.17158211257560	20.91554021358165	17.77898960437317
C	8.90433871499241	19.99301411138471	18.52188109171153
H	9.61210662605434	20.34089772599866	19.26303840102980
C	8.71070938956067	18.63825185176781	18.29023734285428
H	9.27222704154440	17.90115649574464	18.85045335287985
C	7.78629525951138	18.23986846534057	17.33127225486069

H	7.59853823391696	17.19540157641637	17.12661183389227
C	7.09919221171879	19.21827691971466	16.62489834424082
C	5.21006236786283	22.66937077056246	11.76612007766315
C	5.72642224228323	25.85616604098098	15.36030715006700
H	5.26495492079892	26.74800503501507	15.79923694380349
H	6.78370296488873	26.08171971729365	15.20261341377783
C	4.25057753241732	24.57596785364909	16.76535165108775
H	3.56946762257062	24.61426601070285	15.91103064251242
H	3.98436726613359	25.39956521348581	17.43715535565710
C	4.01910358527963	23.24167069451633	17.46954199329421
C	6.53091567841806	24.91312182475157	17.43880998032680
H	6.18492741937969	24.26452444186922	18.24395405048330
H	6.44839372382571	25.94522324969019	17.80102128340983
C	7.97757353685687	24.61155434096536	17.11444793915422
H	8.29855840346799	25.19530467868206	16.25144298217038
H	8.60904281100750	24.91468515686640	17.95858732535200
C	9.47440782909311	23.05748334260186	16.06410612951592
H	9.75063702287481	22.00079176558561	16.01108793601026
H	10.28653068070031	23.58123339754622	16.58062783636106
C	9.36201452418047	23.56839467617407	14.63037863711383
C	8.26802782480974	22.39279005155982	18.03910078363694
H	7.41966114796814	22.67636678544499	18.66642939424485
H	9.18120365689743	22.61752749456320	18.60197130600961
C	6.05931260449484	18.87118824678234	15.57871733361206
O	8.14929420324295	20.64938590267002	13.93967532978424
H	8.86286327848873	20.41107803034970	14.54642799785268
H	8.58130124748436	21.17756028563988	13.25443575688453

Table S41: Cartesian coordinates (Å) obtained from geometry optimizations for $[Ac(OCTAPA)(H_2O)_2]^-$.

E -2123.581409666386 E_h

Ac	6.24845200651585	22.23593615983949	14.96354165230839
O	4.64415380791101	22.48923672250887	10.54726275642811
O	5.75622027419084	21.98221579712687	12.42685816536830
O	3.03614566511259	23.20432255245494	18.20620653598449
O	4.74724610347617	22.29109167164700	17.08313469340503
O	10.50812666842176	23.92923616171508	14.12637453837253
O	8.29160012780392	23.60047402326680	14.11553309206910
O	5.93402813330944	17.55844486224990	15.43726312956642
O	5.58061559068167	19.72881429441578	14.99941743831941
O	3.50896621394044	21.99715220722944	14.44242487329430
H	3.10159270554517	21.51931051958860	15.17765154232101
H	3.34669357631630	21.44507154671841	13.66558129130636
N	5.29734464422776	24.40719499815175	13.46236120557943
C	4.80817862620968	24.15190370174692	12.23905256594332
C	4.10406254898083	25.10182347557155	11.51034246359462
H	3.72601356824985	24.85465616951834	10.52854072345912
C	3.89997578903451	26.35572412773847	12.07442506095094
H	3.34512922394229	27.11548726004808	11.53831457535950

C	4.41577728199267	26.62087071441803	13.33531457841723
H	4.27804440217473	27.58503823002509	13.80727899342920
C	5.11958269149230	25.61989702211804	14.00096366809773
N	5.64769484665437	24.72370079289150	16.24537544531744
N	8.22219148549665	23.17606397701681	16.80635208704315
N	7.33362973088829	20.49037724159223	16.87118482823155
C	8.18078644445485	20.91335697885144	17.81774152359922
C	8.91208189710364	20.02000667042865	18.59755870657471
H	9.60117750353298	20.39684630510326	19.34218067454182
C	8.74113770128253	18.65724809288534	18.39931514017394
H	9.30144907302288	17.94284093571548	18.98934971974997
C	7.84033786733216	18.22202091003309	17.43424170986048
H	7.66955005209958	17.17024916914137	17.25383719332415
C	7.15550720917541	19.17211017266252	16.68780020405433
C	5.08110994694558	22.76881799662359	11.68291717640261
C	5.76837463417527	25.87419415498884	15.33322105075228
H	5.34767974663559	26.77954460455211	15.78605820001103
H	6.83044067539029	26.06040499400741	15.15801165055247
C	4.25826186039142	24.60585889281326	16.71438270739512
H	3.58788365352397	24.66090499294891	15.85196910451773
H	3.99285934542509	25.42774271040924	17.38893134009666
C	3.99108639479049	23.26930377730587	17.40352026283890
C	6.53447294787121	24.91029285020645	17.41681054896872
H	6.17417318101602	24.25563621343287	18.21089719568406
H	6.45312504401332	25.93911317946264	17.78950580876964
C	7.98636018229847	24.60618639194895	17.11232946801346
H	8.31892242721858	25.18714943454224	16.25185594984185
H	8.60453734628289	24.91520029906656	17.96450369956420
C	9.50709014067333	23.04431885374123	16.10195416843941
H	9.76361922466031	21.98294540632164	16.03079386247837
H	10.31613519541536	23.54230417237038	16.64808315623114
C	9.44046044786518	23.58330037402957	14.67425814576040
C	8.25529026830512	22.39617838067792	18.05524647003591
H	7.38672513183651	22.67922293856907	18.65468546324853
H	9.14933110967815	22.63535697276120	18.64305813831835
C	6.14859798115734	18.77409071537697	15.62585935853713
O	8.21690018464585	20.63317291431156	13.76104680314704
H	8.56219450896837	19.86868495234417	14.24039738377874
H	9.00220803394278	21.12925304996689	13.49409067276991

Table S42: Cartesian coordinates (Å) obtained from geometry optimizations for $[La(TPAEN)]^-$.

E -2121.166341142741 E_h

O	5.77106263844010	7.01758118751406	6.60828769329803
O	5.63706499901137	9.42790646318332	10.85018869689840
O	6.10480598490568	8.61057221609369	8.15011415300839
O	7.42397793769854	11.91390758077810	11.20349450110708
O	4.60127718873300	12.53445906736026	7.14401461270761
O	8.25665620842263	13.46995220648420	12.58450780831230

O	5.97995998458972	11.67873220691941	8.68980133839097
N	10.03420198689135	9.92279967541482	7.75459203730762
N	8.29380624245803	7.40790377559140	9.07785458192489
O	4.46216768620444	8.93244297159328	12.69319900471703
N	9.51946231215241	12.09009710641593	9.56522259061806
N	10.17053907131853	8.95615741797188	10.60561542182548
N	7.94889836287082	9.28312667391822	12.16406667810351
N	7.58364128978073	10.64451446693230	6.83336919507136
C	6.82230702862637	9.15502181858609	12.87968506547234
C	8.00723405966411	5.57259005712466	7.56619813782215
H	7.43474223418697	5.12960727913262	6.76394141707281
C	7.62079390204473	6.79897375854040	8.08583247530252
C	9.37897429533224	6.80940917281060	9.58444289599242
C	6.40176986006770	7.52444865955626	7.56077398817424
C	6.47158293375309	11.26334731797933	6.41079829535692
C	8.38099328751351	10.05626438897277	5.93809298370538
C	9.12222193684702	9.32890845064199	12.80131015360173
C	9.49894461303008	12.89620241721568	10.64074760811167
C	9.82516306216730	5.57470475863012	9.11798699085306
H	10.71028986142124	5.12806474703087	9.55131928964191
C	8.30602478309742	12.75680787626198	11.55990276655406
C	6.82874233760381	9.05214880584687	14.26340407946504
H	5.89566714936727	8.94525573462550	14.79813462587343
C	5.53277626287236	9.16578649929429	12.09198415316450
C	10.50496811677451	13.81409152394400	10.90491036246316
H	10.44201220336106	14.43500100252908	11.78703706275390
C	9.60096694270294	9.35437925315674	6.46729832555877
H	9.36543351647963	8.29897543880130	6.62325657893659
H	10.40269608824831	9.39752794171051	5.71945322202333
C	6.11640733503851	11.31970097863835	5.07105795150031
H	5.21210879867036	11.83257780492616	4.77586807885148
C	11.12240780453514	9.08776013156988	8.30548119912967
H	12.06126487293582	9.28748156232125	7.77368647312684
H	10.86905466533102	8.04550709849666	8.11830000537697
C	11.57404123864672	13.90392282936816	10.02251100226330
H	12.37800797776645	14.60699191786779	10.20041105705169
C	11.59940493008680	13.07103549258787	8.91508041108262
H	12.41859356168951	13.09936946694175	8.20862400332780
C	8.04854065163564	9.09817997448925	14.92826523098404
H	8.08990126669453	9.02336547447828	16.00763380254766
C	5.60858967436126	11.88017735668660	7.48713620296950
C	10.06649821123980	7.48534972294207	10.73354246787112
H	9.48065281273301	7.26595010792781	11.62858992321223
H	11.05873316058641	7.04648308505365	10.88348987355287
C	10.34906449363398	9.52154190358898	11.95472288986750
H	10.54337247671394	10.59195586079907	11.84922895241907
H	11.21849383708683	9.08424052076224	12.46062974633607
C	10.51931940494647	11.29710801157507	7.50253870259163
H	9.84035288107528	11.76753283923543	6.78889751611495
H	11.51042793902628	11.27030647439013	7.03663258163278

C	10.55423203595326	12.17132536005151	8.71938081054579
C	9.21252621141940	9.23742218117115	14.18820935449850
H	10.18270173610857	9.27113936360336	14.66676463357719
C	8.09275378247933	10.06567664361961	4.57508271003433
H	8.76784154061165	9.58381491656481	3.87967366842622
C	9.13074876912817	4.94913581000894	8.09476999051366
H	9.46305894329310	3.99328576635025	7.70973974624029
C	11.34716903970940	9.31591089230882	9.78581530528664
H	12.21931429001298	8.72880500649771	10.10111562684301
H	11.58041771334035	10.36125631006351	9.98105221378632
C	6.94229683080116	10.70300452122439	4.13781740636055
H	6.69402233327203	10.72533854190797	3.08415940429270
La	7.71925341079723	10.03307615341830	9.52405226659342

Table S43: Cartesian coordinates (Å) obtained from geometry optimizations for $[Ac(TPAEN)]^{-}$.

E -2465.079170568959 E_h

O	5.79329061467475	6.93958577684052	6.57437772277225
O	5.58005676876982	9.42538544153343	10.95769710568533
O	6.04765917586505	8.50843188857223	8.15688929329116
O	7.38673897707745	12.03556442098114	11.18124553832184
O	4.51357587866555	12.49749567539710	7.00289608090665
O	8.28801206605363	13.51612528339399	12.60336845389084
O	5.86644246292451	11.68274704268569	8.59346907345195
N	10.00728742288364	9.95158512700837	7.74737498040885
N	8.27966137061487	7.36048802320277	9.07600131482767
O	4.45252964636329	8.99571092722212	12.84638759714147
N	9.51672220546356	12.13716666261375	9.56883544822072
N	10.14471989025595	8.94194295818850	10.61450202100910
N	7.93113236376071	9.30793984109931	12.22358426041103
N	7.53296219826539	10.64885915043866	6.77769067515513
C	6.81954536228394	9.18534952939511	12.96432198605209
C	8.05314587722134	5.53919342037104	7.53889110034873
H	7.49858140684903	5.08953560527671	6.72779263174811
C	7.62878634745703	6.74764525177242	8.07252884504277
C	9.37978187744466	6.78822432167665	9.58111366523339
C	6.39136463498878	7.44582751710558	7.54793812587815
C	6.42447512721369	11.25593315365542	6.32770559660242
C	8.36787669201729	10.08212093146156	5.90221914664636
C	9.12127127320207	9.32623963977031	12.83141297289948
C	9.51702893314146	12.92756196777519	10.65536769645777
C	9.86300446905042	5.57264315258261	9.10173204842124
H	10.75827682935102	5.14497486091536	9.53345630451889
C	8.30832894446942	12.82558272965506	11.56236796319978
C	6.85857438317348	9.07173457134940	14.34696754562595
H	5.93779151007629	8.97272112508004	14.90398101379467
C	5.50839105484096	9.19901576138443	12.20934871862238
C	10.55874877009443	13.79891615891191	10.94144407228968
H	10.51570153804760	14.41024245870096	11.83143524672574

C	9.57988217573868	9.38414047287696	6.45623756488151
H	9.33807661279670	8.33027061444531	6.61437131536905
H	10.38985252064760	9.41913934779883	5.71681812890885
C	6.10822935873628	11.31629644313443	4.97812943167795
H	5.20499402658349	11.81672960360073	4.65948148401821
C	11.08697838108937	9.10481201410150	8.30089237825409
H	12.03029236318686	9.30445506265697	7.77653506925145
H	10.82843272467869	8.06666044611468	8.09963865425947
C	11.63767740662620	13.85673628733526	10.06838760723240
H	12.46794923889005	14.52392190406596	10.26319975338934
C	11.63950068414902	13.03982890798738	8.94809078612553
H	12.46567131706565	13.04572022190525	8.24925489366428
C	8.09500503468624	9.09161224100289	14.98166364310516
H	8.16119618011129	9.00511577244056	16.05897561761941
C	5.52304108051906	11.86454443965678	7.37921012994485
C	10.04155539502183	7.47153680310386	10.74306199331848
H	9.43683711418486	7.25537010338389	11.62649374496883
H	11.03169014063894	7.03519862928242	10.91476136720730
C	10.33488406464215	9.50994847735964	11.96209916382757
H	10.53073579720358	10.57953796408201	11.85111177502639
H	11.20970524325342	9.07336308333561	12.45954653513900
C	10.49820755717975	11.32560027697408	7.50381039973046
H	9.80888035361142	11.80729201646456	6.80747746291718
H	11.48139379754725	11.29712898895580	7.02116128852682
C	10.56087533071875	12.18581525002516	8.73178016801536
C	9.24323072335878	9.21638913123967	14.21477188507313
H	10.22514714444669	9.22579793783592	14.66993705663037
C	8.12174939977114	10.10266320950800	4.53099789734755
H	8.82628058662610	9.64090924723391	3.85133228749807
C	9.19078433540708	4.94128770196630	8.06662247344781
H	9.55130775635432	3.99977192465004	7.67163399602044
C	11.31372318053188	9.30976621409414	9.78558235391226
H	12.18785796089530	8.71703996014638	10.08532839983449
H	11.55098494159714	10.35150482118331	9.99439768110071
C	6.97321942118322	10.72334178500507	4.06525287084780
H	6.75684707470527	10.75223833095188	3.00468135019411
Ac	7.64925850305440	10.04942398807693	9.52372714611215

Table S44: Cartesian coordinates (Å) obtained from geometry optimizations for [La(TPADAC)]$^{-}$.

E -2277.296498102023 E_h

La	7.69417601143390	10.03662592795762	9.51446439912006
O	5.75433891583266	7.10217828452740	6.52287555140552
O	5.64010448910867	9.44755007580312	10.87902702808528
O	6.02977479855074	8.59125049559576	8.17924164142632
O	7.38853714280880	11.95811843645930	11.18469910337599
O	4.49272599133462	12.38309658295646	7.12661174451383
O	8.32193013269588	13.33043489184361	12.69420854164569
O	5.94267719773820	11.65134051590329	8.67098624632669

N	10.03188376928021	9.91855718897375	7.73325409985034
N	8.25284319403541	7.42947496281355	9.01629606039417
O	4.49326406940519	9.10101250203014	12.77296478498446
N	9.50649878006715	12.06485100518433	9.60195507147906
N	10.19026036714096	8.94041075162809	10.55914764825896
N	7.97322545002305	9.36797723295009	12.15743162638133
N	7.52018710396872	10.56458058251656	6.82561797890912
C	6.85886992634944	9.30208393542532	12.90128774682030
C	8.06372211621859	5.68059924503450	7.39729801512296
H	7.52261542616863	5.25991768835195	6.56174520016786
C	7.61674785315766	6.85695815107289	7.98313475592663
C	9.34566601074698	6.84144426271352	9.51872807796858
C	6.36725029173715	7.56566406270598	7.50886010783362
C	6.38080067271171	11.13284215806642	6.40287130735161
C	8.32149987522333	9.97132445793210	5.93833272367425
C	9.16019911746107	9.42448593355110	12.76588282507691
C	9.54117663271202	12.80613057176723	10.71950048821698
C	9.85181416583959	5.65830201513672	8.98920829006074
H	10.74104420037514	5.21646597172577	9.41861478705952
C	8.32888999835735	12.69625622630888	11.61756279255783
C	6.89150746479669	9.28377017526879	14.28771258116895
H	5.96775483059593	9.22473971965153	14.84546120013234
C	5.55522736686970	9.27735965269442	12.13862904939839
C	10.61131520993881	13.63505767713686	11.02721471223347
H	10.59870160913040	14.20770479302646	11.94361104948502
C	9.58423219854020	9.33785534848805	6.45268007224825
H	9.40809452786208	8.27143805538472	6.60965808009052
H	10.35392749117393	9.42156045538321	5.67723009199849
C	5.99613303305643	11.12224878993744	5.07085067754202
H	5.07028136324872	11.59467317324286	4.77517158945105
C	11.19706708561466	9.11256034355903	8.23116835694799
H	10.94006585739242	8.07627192477153	8.00498238881387
C	11.67575339475246	13.70528172080557	10.13735072027811
H	12.52714572709071	14.34010172371087	10.34785332229848
C	11.63250451578141	12.95011784969830	8.97382270666938
H	12.44035519766650	12.97647740453205	8.25468666923015
C	8.12566717092913	9.34898340097111	14.92452351035922
H	8.18802461597219	9.34111762737054	16.00536380696891
C	5.52895324268319	11.77700526084582	7.47127202905872
C	9.93338067812843	7.48979112443771	10.73281986898825
H	9.19428033470424	7.38868606039505	11.52786923087989
H	10.82296738787014	6.95211894786329	11.06293881420133
C	10.38478147141345	9.52798656489811	11.90045878584305
H	10.63704650532615	10.58322317543492	11.77089684798275
H	11.22265830046494	9.06366859468188	12.43128512112622
C	10.37541682424067	11.33957082936111	7.48403077920464
H	9.54309293534782	11.77718814602246	6.93317510608549
H	11.25919652315541	11.44216989163152	6.85310983169214
C	10.52909698991215	12.13509529208629	8.74090070290646
C	9.27597933451576	9.41660280764551	14.15479211299395

H	10.25642690597280	9.45776785400808	14.61125761281054
C	8.00417474384235	9.91662654739063	4.58207243317010
H	8.68190811283518	9.43162930660101	3.89153448478316
C	9.20486343360381	5.07521762615471	7.90858778916381
H	9.58383044729776	4.15992643523654	7.47178282174269
C	11.42144826194649	9.22434952378017	9.74494686748113
H	11.67857471671271	10.25959419217992	9.97627878471586
C	6.82267906725767	10.49383558464995	4.14554425691822
H	6.55142467247139	10.46332327670732	3.09785904836600
C	12.61495686504282	8.34054647314100	10.14881841269330
H	12.38869261641578	7.29859413593106	9.90668228616340
H	12.76445513284987	8.39907850656230	11.22856636658797
C	12.50411523817651	9.44558778424302	7.48991873691127
C	13.89553891022059	8.71023699379049	9.40871001003100
H	14.71287511002674	8.07044883296697	9.75118626544299
H	14.17630560547265	9.74562772857210	9.63057404890297
C	13.66569664982735	8.55299923255305	7.91121846540045
H	14.55938809361341	8.82601325028005	7.34407179204616
H	13.44189524548563	7.50531167518385	7.68255992052406
H	12.78952451806583	10.47746118944344	7.71127457511007
H	12.33713376420424	9.37804920275090	6.41282456076191

Table S45: Cartesian coordinates (Å) obtained from geometry optimizations for $[Ac(TPADAC)]^{-}$.

E -2621.209439530558 E_h

Ac	7.63690138234499	10.04465425545834	9.51772360491116
O	5.79126878739685	7.03719391405763	6.46944085812665
O	5.59293363137039	9.42886982312489	10.98150195492297
O	5.98786402536403	8.49876689657987	8.16215114026871
O	7.36790323600741	12.04872156131691	11.19188441165954
O	4.41897131292786	12.34441706486174	6.99960042575238
O	8.35694904322686	13.34459579468334	12.73454883688059
O	5.85154289863958	11.66414391037038	8.58406538310359
N	10.01484249821852	9.94399220050993	7.73228477019396
N	8.24242444819211	7.38294287180921	9.00779697986190
O	4.49197355453960	9.17734850857956	12.91755276134208
N	9.50442304375501	12.10600139781778	9.61682030508791
N	10.17112819880335	8.92519991841228	10.56670452807783
N	7.96319372118540	9.38029676432954	12.21396449020858
N	7.48631623163360	10.57698172376040	6.77515558071403
C	6.86308759436331	9.33190406138381	12.98097248037713
C	8.11646832471653	5.65540676016818	7.36200026749626
H	7.59705148174170	5.23173172841604	6.51423181855493
C	7.63165964402721	6.81109803973185	7.95970875787794
C	9.34709840741604	6.82006884719377	9.51331595370275
C	6.36939310873523	7.49624757906689	7.47814862221991
C	6.34709391130788	11.12765221620147	6.32782106615818
C	8.31816214238515	9.99732532958086	5.90695346554936
C	9.16458088797620	9.41871790969273	12.79541588196468

C	9.55312863845676	12.83280801044766	10.74274088537932
C	9.88878396912533	5.65728064825096	8.97408797063782
H	10.78489495506757	5.23171960615599	9.40589571102353
C	8.33636728592832	12.74149288192211	11.64013660663316
C	6.92595878207425	9.31916726529949	14.36653100992403
H	6.01424596759017	9.27659392008093	14.94520699925088
C	5.53942339340644	9.30889133917300	12.24939145206726
C	10.64401916015725	13.63069698525985	11.06135919632600
H	10.64558613866556	14.19446182165017	11.98338581695652
C	9.57462333385753	9.36874856294137	6.44492328815984
H	9.39322540716301	8.30264764880238	6.59916592305603
H	10.35361446117893	9.44875449933911	5.67837470769712
C	5.99288949352119	11.11064491543237	4.98731754760268
H	5.06579585166537	11.56670068482262	4.67051220103186
C	11.17449781264568	9.12693691209862	8.22944531790753
H	10.91544014916701	8.09505258438271	7.98652662901950
C	11.71118584982293	13.68270883246294	10.17326599840056
H	12.57714409560052	14.29510397477064	10.39130926785837
C	11.65380970694879	12.93897463610764	9.00257171222481
H	12.46497631955835	12.95246257522187	8.28663401800859
C	8.17499014358603	9.36379755906884	14.97582676562061
H	8.26045384021008	9.35642373137447	16.05521924876250
C	5.46027154036389	11.76519935127295	7.37420834508614
C	9.90878961947249	7.47497577251054	10.73868624720937
H	9.15254157390681	7.37981941962617	11.51861985835669
H	10.79127915844423	6.93782897868442	11.08914673089645
C	10.37638411972012	9.51095140513580	11.90854118533997
H	10.63163019972403	10.56537596136108	11.77663446705405
H	11.21865236284830	9.04414142139754	12.43023610469741
C	10.36062850303139	11.36730087867235	7.49590817958288
H	9.52301345849411	11.81154994164793	6.95785987469809
H	11.23859301237982	11.47362066754975	6.85687270797126
C	10.53138112550923	12.15351221711281	8.75945908182711
C	9.30974831464729	9.40874222535379	14.18184510064271
H	10.30011605996422	9.43239884641456	14.61789046370587
C	8.03390283281411	9.94241337960130	4.54314563503371
H	8.73558698397468	9.47139689516501	3.86683927598320
C	9.26717244134483	5.07292229847945	7.87892224989889
H	9.67399803812766	4.17331590581239	7.43434530482369
C	11.39891443310469	9.21231790158261	9.74676926877373
H	11.66595206244506	10.24188528074023	9.99221117172558
C	6.85267082481163	10.49958603732478	4.08071800694151
H	6.60678784698770	10.46709252056763	3.02670946177879
C	12.58449238452636	8.31002347871234	10.13299637795918
H	12.34606976434742	7.27520353697032	9.87200099792577
H	12.73391210187708	8.34686871433509	11.21374490518477
C	12.48542586064883	9.46847581834771	7.49894700740320
C	13.86991438086576	8.68089187137863	9.40217626054544
H	14.67987181922873	8.02475767474566	9.73102439182128
H	14.16123963624744	9.70801891063237	9.64761885196602

C	13.64077227016954	8.55932866954752	7.90127264601341
H	14.53732740660928	8.83837070161146	7.34180603137767
H	13.40844844739401	7.51888680572059	7.64924230773898
H	12.77506810718769	10.4939928462373 8	7.74330711813428
H	12.32039803710862	9.42507099357371	6.42029976334037

Table S46: Cartesian coordinates (Å) obtained from geometry optimizations for $[La(DTPA)(H_2O)]^{2-}$.

E -1570.318732634460 E_h

La	-0.04331681092366	0.36568313844893	0.05082144499730
O	-2.07868852811464	1.14643712816993	1.34073103767051
O	-3.66253539545931	0.70686121248555	2.86219368165224
O	-1.58985361094530	0.21858649310978	-1.92184436572671
O	-3.26290109325362	-0.71313163590651	-3.08009164450908
O	1.39001967003107	0.38446719093086	-2.00178152623275
O	2.33245570315486	-0.59079378312151	-3.78098678964971
O	0.17285474815254	-0.83785483559129	2.21854193925437
O	1.23828639247825	-2.12373062988829	3.70750674636276
O	1.71922127313076	1.96584442907740	0.92634396882167
O	3.82889272452952	2.71515910253831	0.84334240781501
O	-0.48158769592731	2.85613677591108	-0.79369976669212
N	-2.16552581190327	-1.37596070867288	0.31974398442279
N	0.42175327277686	-2.08779622330141	-1.06897392470785
N	2.48895456120022	-0.63561897791065	0.66808612497957
C	-1.60913556084543	-2.74204866457729	0.24237481276145
H	-0.99596556975002	-2.89614623120179	1.13080370432252
C	-0.78846353047021	-2.94933852038647	-1.01583474132739
C	1.50102846317357	-2.72652449691908	-0.27548848783027
H	1.79647829685659	-3.66679023878820	-0.75928881088942
H	1.09587610910713	-2.98085000289214	0.70435315546887
C	2.73554033774352	-1.85719085535170	-0.12624627143423
H	3.52734789263008	-2.46057088637968	0.33437796713794
H	3.10294313813596	-1.55538439864727	-1.10650673620291
C	-2.78218315720308	-1.13427415429342	1.63152767371631
H	-3.78044810608577	-1.57919243385882	1.70072514690888
H	-2.15955593138075	-1.59219056073817	2.40101015037405
C	-2.86400926434684	0.35492688290063	1.96759192788532
C	-3.16671857044019	-1.13847767612436	-0.73777730920979
H	-3.89548788502377	-0.41534804477132	-0.36144240643184
H	-3.71603380939565	-2.05629871157348	-0.97001927610872
C	-2.62601642868688	-0.51866637004134	-2.02161983919148
C	0.85305179401040	-1.91284970055145	-2.46985584585523
H	-0.03930497479655	-1.87599864423381	-3.09839813894375
H	1.45542138828583	-2.76272223394779	-2.80726646402839
C	1.60080841924508	-0.61388466043506	-2.76717139789258
C	2.55053835016962	-0.92369004408066	2.11255396422782
H	3.32788845923582	-1.66141219925597	2.33381576650010
H	2.81556105813571	-0.00054043478224	2.63560652988766
C	1.22532495993460	-1.35503565728501	2.72338233930757

C	3.46263393267603	0.41613866256476	0.34344419852740
H	3.60456994358745	0.43951561208880	-0.73829575661018
H	4.43933771928643	0.22766453057843	0.80230561533390
C	2.97572635043182	1.80711919257537	0.75039417803266
H	-1.40881002049541	3.12489641673852	-0.74128768362206
H	-0.00483404494428	3.47558964970659	-0.22391449683123
H	-2.41380038322805	-3.48925945727223	0.25719370212723
H	-0.50399608601096	-4.00605084373223	-1.08632707207795
H	-1.39790168846876	-2.72994750131135	-1.89241341649022

Table S47: Cartesian coordinates (Å) obtained from geometry optimizations for $[Ac(DTPA)(H_2O)]^{2-}$.

E -1914.228013046714 E_h

Ac	-0.05943935172254	0.45554991332848	0.04483122813394
O	-2.17882862974944	1.13948464156456	1.37146893905400
O	-3.76524478664822	0.63278627276360	2.86918151803968
O	-1.63896349707171	0.19145544930221	-1.96323897978855
O	-3.32048685237293	-0.76703574736479	-3.08742265981939
O	1.41124747814019	0.36918970750045	-2.04722105677660
O	2.39047462643670	-0.65322321595070	-3.77977371916738
O	0.19434101821229	-0.81993321708988	2.23860924911595
O	1.26376155325569	-2.13327363577634	3.70101215216883
O	1.83519548880017	2.00089982125169	0.92411848752907
O	3.96942465948291	2.67343268471343	0.81278902951346
O	-0.54082024411919	3.04409024117053	-0.73168718943771
N	-2.19415187540049	-1.38133124462838	0.31289035478414
N	0.42492753122686	-2.08792022366656	-1.07490773200200
N	2.51774127235862	-0.63748588971826	0.67038572749759
C	-1.61594152217307	-2.73813211838391	0.23053310774796
H	-1.00266231759100	-2.88513194972510	1.12023183189772
C	-0.79036692820238	-2.94121606190595	-1.02567974723426
C	1.49113553734059	-2.72313747586718	-0.26261409179551
H	1.78151902551309	-3.67419903237098	-0.72909112221106
H	1.07630141002010	-2.95938632570037	0.71754222681635
C	2.73766787310111	-1.86927938135192	-0.11618230461756
H	3.51928682465021	-2.48423594106381	0.34739033469822
H	3.11081302785085	-1.58051382211546	-1.09829409829103
C	-2.81431996590928	-1.16559479143220	1.62801736079613
H	-3.79601672237355	-1.64615976090136	1.69924763853144
H	-2.17288386581587	-1.60691207073754	2.39250026010111
C	-2.94675570533156	0.31682974140113	1.97887816181368
C	-3.20137662726780	-1.15452176642392	-0.74019693018092
H	-3.92370174175745	-0.42318488294990	-0.36695886058166
H	-3.75620907220091	-2.07332443981288	-0.95637769229113
C	-2.67321804450201	-0.55234730692003	-2.03917660170752
C	0.87152949023750	-1.93573438718885	-2.47287829914613
H	-0.01453313782630	-1.89782484312914	-3.11062215555936
H	1.46798260731475	-2.79617177301934	-2.79451975785891
C	1.63517981492103	-0.64874168161353	-2.78303608615284

C	2.57404920987697	-0.91752967798437	2.11604736984196
H	3.35213591720899	-1.65290470208395	2.34424421994076
H	2.83566143472807	0.00952260642841	2.63398065258184
C	1.24804005329778	-1.34978346970107	2.72822757075275
C	3.51844685118473	0.38515330586392	0.33586910068299
H	3.65205536403466	0.40029187677950	-0.74747302057330
H	4.49395351146736	0.16901025332807	0.78578710076927
C	3.08258578822573	1.79509577103219	0.73727889465016
H	-1.46213096625326	3.33209842892515	-0.67823102808400
H	-0.04344980382382	3.69415964840967	-0.21686274595133
H	-2.41039513555142	-3.49666328296830	0.24489262418478
H	-0.51221005229169	-3.99997604235423	-1.09763619386749
H	-1.39590952293112	-2.71678420186270	-1.90363306854809

Table S48: Cartesian coordinates (Å) obtained from geometry optimizations for $[La(TTHA)]^{3-}$.

E -1855.425861633567 E_h

La	10.33855412466325	3.90770494015724	4.82616076021233
O	13.32242626998452	6.03225921231344	2.04594296563290
O	12.49647819319338	4.85636254494150	3.76719620917883
O	11.63443616217841	7.42350513978592	7.62770963456977
O	11.20458968057857	5.44007898660638	6.68491553492195
O	7.56484906523479	4.14928157636933	1.08652341159205
O	9.05428294650543	4.55009510534274	2.70857212727458
O	7.95110361434102	3.60035273099364	8.81476246124431
O	9.25736320397938	3.27382340562907	7.02860721739749
O	12.37459583320188	1.21173060456743	7.91294786721381
O	12.11460622707375	2.63901670965885	6.20524556927474
O	11.88881903884530	0.71451438183769	1.70204236095741
O	11.03054558053414	2.51722519207230	2.72089145206139
N	10.53843400859508	6.64928413849754	4.24791122862905
N	8.19078390228902	5.49504147109785	5.71714558382268
N	7.86039699147054	2.70524674009220	4.39593523810595
N	10.34483030244926	1.06140551111257	4.93065549268765
C	12.45392392847300	5.80906980319462	2.92040239076464
C	11.22870859827445	6.72432728677781	2.95581779776787
C	11.32790110980853	7.36316475961832	5.26710575211145
C	11.37115280839906	6.69866338799777	6.63766020556025
C	9.17122775918348	7.19515614633675	4.15565615659316
C	8.38401181598691	6.93822722537491	5.42508419715641
C	6.99790100575313	5.00570423371080	4.98914867809795
C	6.80978430492743	3.50051265245135	5.07339880819677
C	7.59805422211718	2.66935580664955	2.94929008113364
C	8.09688584255178	3.89444233055269	2.19383636876818
C	7.85923483415506	1.33486278973393	4.96238951856610
C	9.04336079976713	0.50032396094757	4.51477400407230
C	8.00334703282417	5.31105878086039	7.16650067341395
C	8.41988661276740	3.94772696537730	7.70428808119246
C	10.68758455633887	0.70644745299223	6.30927939087053

C	11.81730701608089	1.58111355912885	6.85315467676682
C	11.39327007267845	0.56246251625647	4.03132830183764
C	11.42867671645604	1.31123186932868	2.70587034390212
H	11.53499778889966	7.74797516968258	2.70976951792355
H	10.54653752426333	6.38382497187582	2.17473840590990
H	10.99111025452837	8.39999277656515	5.36897938272607
H	12.36991223826769	7.40035515327702	4.93800247451287
H	8.68785971149834	6.72613237628329	3.30045646896357
H	9.19830285095983	8.28034870564072	3.98202505864361
H	8.90726727798638	7.38375722966307	6.27150658250337
H	7.41374505638740	7.44580380777100	5.35379830182258
H	7.07541047672285	5.33022945592758	3.95333292125698
H	6.09827586919046	5.47611415622865	5.41074679511989
H	6.77796768666291	3.19095324353966	6.11743614965399
H	5.82853378393386	3.25676615912162	4.64538843506126
H	6.53166368700386	2.51842120334442	2.74525523842742
H	8.13413659799189	1.82591355037940	2.51020096882251
H	6.93584565616310	0.80622900673645	4.68855124250773
H	7.86956500452917	1.43891196865541	6.04705456765254
H	8.91945079226505	-0.51934202039978	4.90238450500875
H	9.05838682755567	0.41649625942523	3.42696496321248
H	6.96894170045111	5.51911537338666	7.46394497874905
H	8.64089357245837	6.02784080854791	7.68818998454017
H	10.97831874302148	-0.34837744646526	6.39794957607846
H	9.81982205750696	0.86330202863604	6.95005577129785
H	11.29015996174979	-0.51435745926245	3.85414548979534
H	12.36648069834115	0.72461360307389	4.50167167826066

Table S49: Cartesian coordinates (Å) obtained from geometry optimizations for $[Ac(TTHA)]^{3-}$.

E -2199.338605176344 E_h

Ac	10.42942856044439	3.89192312776823	4.81516222619299
O	13.30588657258722	6.25399230870302	1.97435403566129
O	12.52130721999985	4.95071495948530	3.62130880898353
O	11.61377515734814	7.51138417468780	7.62328160071165
O	11.29191132427370	5.50760009742794	6.67922072949279
O	7.54576872395596	4.14186476050336	1.07733304192318
O	9.08360057337944	4.52402809019874	2.65866548098694
O	7.90478432791100	3.59257179672970	8.81594112995120
O	9.27486249991687	3.28731258497702	7.07290911157896
O	12.32853451101942	1.06636279114450	7.96228688757715
O	12.14736869496933	2.52514484668683	6.27125672492152
O	11.90019500368050	0.55447457723447	1.72773719728671
O	11.09213837000076	2.40513981288873	2.70080491860504
N	10.54476267977342	6.71573702308683	4.24297743254212
N	8.20351408616296	5.50316461106206	5.73442032811051
N	7.86998977863131	2.69908856411582	4.38563679593338
N	10.34523241139894	0.99863677401038	4.93991203687875
C	12.45054706802543	5.95545457999904	2.83938737496932

C	11.20378664487606	6.83675387331171	2.93772441693790
C	11.33118117047188	7.43668282108479	5.25899089384293
C	11.39432075462343	6.77408391525062	6.63222795911650
C	9.16059480720413	7.22138267508132	4.17514037103674
C	8.38999735240041	6.94757736325469	5.45116831427776
C	7.02507202927618	5.00921370960061	4.98666411651538
C	6.82990154021192	3.50401250146272	5.06729085662460
C	7.60267503718559	2.66301946409599	2.93990588853532
C	8.10201808223287	3.88185138767297	2.17165293407115
C	7.86056924755177	1.32980113530041	4.95489956213764
C	9.03256727339445	0.47640616152666	4.50741146369934
C	7.99823616298656	5.31352340688790	7.17985269079702
C	8.40841907003534	3.94908298146262	7.72289238880950
C	10.65389875687865	0.63701490257211	6.32583309189152
C	11.80411002649231	1.46823765213324	6.89761983315187
C	11.39033840568112	0.46482659025841	4.05700687198997
C	11.45324541280763	1.18651496602436	2.71562379293184
H	11.47519754976975	7.87377236690125	2.70756025315978
H	10.51132473197589	6.49614637979383	2.16533282150165
H	10.98528778295649	8.47047892681487	5.36269365820263
H	12.37091187151987	7.48194511853327	4.92289248858563
H	8.67963621133002	6.73973524188361	3.32486898907770
H	9.15473602078649	8.30674958142063	3.99930502981247
H	8.91853293328993	7.38908753717563	6.29632643455596
H	7.41734420135550	7.45331797421252	5.39241170745587
H	7.11978410527536	5.33258794263482	3.95178533979736
H	6.11769655457638	5.48040064381520	5.39127077664708
H	6.79756625289590	3.19223326018241	6.11054627435625
H	5.84519610211072	3.26948923970914	4.64156089742648
H	6.53498203390173	2.51523948182668	2.74032793405011
H	8.13438208076327	1.81628202871769	2.50141953934661
H	6.93116959574080	0.80855062104172	4.68678009910036
H	7.87488568248771	1.43654458654231	6.03943526308396
H	8.88155534528440	-0.54445676693692	4.88256802257543
H	9.05461244462191	0.40364770532341	3.41907312988643
H	6.96039385523462	5.52128279143799	7.46592387477664
H	8.62935081853464	6.02954150852807	7.71116358290583
H	10.90202255983961	-0.42785853112483	6.42102535699019
H	9.78090344807207	0.82839911158428	6.95033144203073
H	11.26843363864604	-0.61301904353416	3.89841460651254
H	12.36245484124135	0.61712130582539	4.53318116948796

Table S50: Cartesian coordinates (Å) obtained from geometry optimizations for $[La(HOPO)(H_2O)]^-$.

E -2766.615497289566 E_h

La	49.69302360426309	38.21682383010718	42.39760012315748
C	54.54446972889289	40.24430874613743	41.46337941861130
C	54.02308327390936	39.90811031678140	42.83155231122056
C	54.43647537140590	39.59659298948885	45.17473244940342

C	53.09202449241980	39.39517044529713	45.38867314885424
C	52.17095140954302	39.42413613670173	44.32073552414508
C	54.90839003944230	39.85388104558096	43.88672121913358
C	53.98343450905266	41.10299757086662	39.23026155675398
C	53.81582877797839	39.83896075956886	38.38921374352489
C	52.43570417383039	39.21187250697871	38.57241189840666
C	52.61167445193313	36.78261803954003	38.33701213833239
C	51.85893120201646	38.09876248597214	36.41589374964829
C	50.33605692347548	38.06019093256202	36.26919045135259
C	48.27269823283234	36.53691889384900	36.28557244329959
C	54.45703370909617	36.54425252594297	40.01770727683454
C	54.87811031932475	36.41650050848889	41.34233623675430
C	53.95586482993517	36.45974440972061	42.36510187432140
C	52.58561401901149	36.65337530246015	42.10408063596451
O	46.99568445477026	38.90018224251671	37.12614230263602
C	47.09060907082461	37.93503402036426	37.90437987529531
C	46.36699287118078	38.00805376638477	39.22365088029353
C	45.00973927532367	37.78400527706941	39.30119323095008
C	44.37244186816434	37.91814550720980	40.53626400187277
C	45.09612887335836	38.28987675373536	41.64829723687996
C	46.48281012953450	38.52729974902739	41.57302945510100
O	47.23592274242770	38.85714947459985	42.54246088041997
N	47.07234595207459	38.36662958938745	40.32207410210487
O	48.39817651049221	38.52715187217886	40.22803970123893
N	47.70668547417573	36.78416970245772	37.61643254405663
C	47.85913431111126	35.71868532314601	38.60911833542900
C	46.75866953186926	34.66538451942044	38.55372820765268
C	46.98543056103207	33.59233569737098	39.61839140138397
N	47.08830771882904	34.15971999914563	40.95761402296279
C	46.01592752070073	34.51301402045969	41.67446359721019
O	44.84664801731905	34.23437473441857	41.36007818089410
C	46.27322524599114	35.20517332310671	42.98176362701001
N	47.42557239618195	35.89769480985930	43.22635526218828
O	48.37459393536021	36.03231814430372	42.29048168863958
C	47.65997366305244	36.56635965051167	44.42448607615679
O	48.73154869027857	37.24692220397036	44.52216954533786
C	46.68098763924527	36.43686570027251	45.42817783559877
C	45.52156400147923	35.72906163882893	45.20270384074968
C	45.31060259047602	35.11947928727584	43.96712064742358
C	49.79401598864869	36.63287924812689	36.26093543095511
N	52.29588269277313	37.96928441953209	37.81189317898259
O	52.57902792987273	35.70583437599853	37.71527511884198
C	53.11948336046579	36.74948047122356	39.75562214776371
N	52.22119023922419	36.81162038641495	40.76956395095748
O	50.93104952556855	37.06662250746665	40.50719234473262
O	51.66206560358255	36.71400863801164	42.97766251486203
N	53.68955720172311	40.86073068364566	40.63784030104090
O	55.73706134712860	40.02534375611111	41.19702822878795
N	52.69446031508721	39.69024626469388	43.05903153816239
O	50.92122964800796	39.21765084744590	44.41395992893987

O	51.81702823827746	39.66458229292883	42.04646306527602
O	49.45235461408389	40.92168445886264	42.15646635930312
H	50.42152885261220	40.94836774220370	42.02635830709342
H	49.06646676489218	41.13463206704161	41.29589726187996
H	46.87078855349669	36.93795659084834	46.36775116887929
H	44.77184480007067	35.65243521661020	45.97988372820609
H	44.40912899042964	34.56791737120196	43.74722890414368
H	47.94785558026699	34.63410295123333	41.23799333764506
H	46.16993091839248	32.87113513989291	39.60900259505738
H	47.91543501228437	33.05762230283101	39.42479680087374
H	46.74157828187841	34.18481149318057	37.57272482686864
H	45.78899058812320	35.14140874967183	38.71282508793609
H	48.83214292668260	35.25514980896451	38.43924348121720
H	47.90364189742425	36.17590156137402	39.59496182424057
H	44.47422131594494	37.49623374784952	38.40791683325220
H	43.31067891620415	37.72429611265087	40.62084574266583
H	44.63305223245239	38.39329775365202	42.62021085591797
H	47.81829810861174	37.24423178814978	35.59354400238481
H	47.96009504034564	35.53122670555166	35.99840949355598
H	50.14850735721643	36.12487572642416	35.35946477450597
H	50.19815875354039	36.07521087447735	37.10761763933184
H	49.88582026948774	38.64499771292154	37.07661653556708
H	50.06759201982370	38.55468633793986	35.33142303192344
H	52.25198756444325	39.05065444898229	36.05852131493076
H	52.32484176422347	37.30216980731572	35.83548446379579
H	51.64983657589757	39.89037304068086	38.23584210135542
H	52.24425090932076	39.00067498333480	39.62257949012618
H	53.97789347932505	40.09339990526301	37.33900524297643
H	54.57619998606674	39.10926159026413	38.67620374564451
H	55.00075760065722	41.48130122219708	39.14414522419327
H	53.30109829380201	41.88246133450101	38.89174715117049
H	55.95489618902673	40.02555872094125	43.68509203209301
H	55.12902169130626	39.55788516142582	46.00595938012277
H	52.69587871792546	39.19082339567886	46.37418884798594
H	54.24974324001682	36.36902066620585	43.40209331041239
H	55.92915120511827	36.28444988469981	41.56559240212930
H	55.14965277805857	36.49752160423161	39.18974221229571
H	52.71268900656852	40.76505067607645	40.91316505980853

Table S51: Cartesian coordinates (Å) obtained from geometry optimizations for $[Ac(HOPO)(H_2O)]^-$.

E -3110.526687453975 E_h

Ac	49.70417560894935	38.16871634410271	42.40521282967280
C	54.56323815050050	40.28934570768615	41.45627577544279
C	54.07529780579158	39.91470742588621	42.82766623244196
C	54.55486697358651	39.52212283955799	45.14651143828777
C	53.21848815260680	39.30106946188840	45.38970298805997
C	52.26671746793522	39.36251683280558	44.35034313393855
C	54.98914377910510	39.82862384903576	43.85625622098192

C	53.94349561265068	41.16242883320864	39.24404146151215
C	53.80016561315182	39.90706273642457	38.38546883822565
C	52.43948129444858	39.23941247264394	38.57145425956588
C	52.68684483444153	36.82016515154096	38.29437895227317
C	51.85837513135423	38.13763967834981	36.40821627795401
C	50.33525790359841	38.05070376511164	36.29229001585928
C	48.31337564886552	36.47198596568185	36.32006802677191
C	54.54584064991062	36.61321350065557	39.96741987263985
C	54.97133063358252	36.47584499102886	41.28966950846806
C	54.04842677414495	36.47162778537042	42.31282565016416
C	52.67240146256498	36.62650133392137	42.05632890365101
O	46.99134680957313	38.83268952963923	37.09252068677028
C	47.07890243043479	37.88137373154519	37.88869995532951
C	46.33903098889741	37.97356661189497	39.19733018180668
C	44.98213530197516	37.74676696888113	39.26824147886479
C	44.33656179878310	37.89685929208838	40.49732508331131
C	45.05197592774177	38.28485151102948	41.60911308411319
C	46.43896627329320	38.52508302002841	41.54148959925907
O	47.18478586016523	38.86719620022316	42.51210171061994
N	47.03612318594497	38.35139524659807	40.29474457711805
O	48.36196545947103	38.51760454890697	40.20353956797943
N	47.70713743193232	36.73015431992369	37.63074870685669
C	47.84092809731813	35.67664547111388	38.63940895077949
C	46.73641691983979	34.62758855194968	38.57626114576441
C	46.93444008553556	33.56288657293103	39.65517738864320
N	47.01493379300676	34.14029924502218	40.99156298498622
C	45.93295551124534	34.51277045688009	41.68362075181512
O	44.76684277969814	34.24537774053543	41.34779988511544
C	46.17054635206095	35.21863448717815	42.98813617104641
N	47.33483332886649	35.88111198234609	43.25943068314892
O	48.31772620035711	35.96844431626854	42.35333330908128
C	47.55141807503220	36.56757118978135	44.45238715248799
O	48.64060910787086	37.21569255500721	44.57299990410143
C	46.53449887669635	36.48825589392251	45.42327355013907
C	45.36273992166230	35.80993425292954	45.17223395603235
C	45.17446030606854	35.18094266214996	43.94271410119498
C	49.83251825031207	36.60964832134447	36.33098971189771
N	52.32765926798385	38.00480060530474	37.79304801463046
O	52.68430416261011	35.75404764244692	37.65365899124801
C	53.20216239534764	36.77860356210212	39.70959033388630
N	52.30197699458546	36.79403471470456	40.72388806168818
O	51.00491102671610	37.01154766386691	40.46311351521877
O	51.74860202295670	36.64572628735570	42.93213786659537
N	53.67891977917481	40.89153799628902	40.65203278448323
O	55.75682669808575	40.10510600994924	41.16730751427617
N	52.75383379812575	39.68394386953231	43.08505035228391
O	51.02168744470992	39.14232791976830	44.47141827462244
O	51.84664786117443	39.69839481734850	42.09846241128655
O	49.48041956661555	40.96497738944159	42.23267247731180
H	50.45268270746742	40.97777244854402	42.12392182614989

H	49.11689158614928	41.23717629908879	41.37938770392243
H	46.70682218886374	37.00279203082344	46.35896085191109
H	44.58552454585271	35.77164596994032	45.92490097006363
H	44.26427285148073	34.65220364827365	43.70361070946640
H	47.87530011714129	34.60262993191973	41.29017437343858
H	46.11414286571789	32.84727373959451	39.63456617364611
H	47.86427229623892	33.01996733181845	39.48506485464068
H	46.73488637974427	34.13723103290142	37.59990241349140
H	45.76677431442731	35.11095417877758	38.71147446271086
H	48.81503841418820	35.20818309082088	38.49027403199322
H	47.87004612395918	36.14451577137820	39.62082521713532
H	44.45347780902104	37.44405238561023	38.37581917343804
H	43.27473111121290	37.70160228606361	40.57777833199825
H	44.58158914026158	38.39904020706135	42.57633207590874
H	47.85820309125526	37.15154849915758	35.60113412363189
H	48.03566463233307	35.45235378622268	36.04713513822596
H	50.22454862912257	36.07541201398329	35.46077379541835
H	50.22707051938904	36.09860408311895	37.21141358994924
H	49.88204676428232	38.64443967439416	37.09152262735396
H	50.03832846300107	38.51185657144572	35.34595297674611
H	52.21183893885411	39.10644530410335	36.05475900153982
H	52.33715142490077	37.36358006890605	35.80818135360917
H	51.63211378196198	39.90080470517265	38.25245668686620
H	52.26366500071608	39.00711043357819	39.61990565302516
H	53.94394775801073	40.18182743311025	37.33769084984047
H	54.58296770447919	39.19386879495813	38.65286255975129
H	54.94769959304573	41.57211079842197	39.14747435923167
H	53.23321267668388	41.92615216791287	38.92777210897825
H	56.02897895521140	40.01147510830271	43.63134656685511
H	55.27054861667830	39.45867301453294	45.95638345146065
H	52.85200681181461	39.05658854521144	46.37757140064375
H	54.34593306441192	36.37119298638468	43.34788718899263
H	56.02608155295819	36.37342108952089	41.51097329222683
H	55.23910264019310	36.60385850043766	39.13871246596650
H	52.70991833990998	40.77093123537657	40.94720737806571

Table S52: Cartesian coordinates (Å) obtained from geometry optimizations for $[La(BISPI)]^+$.

E -2638.769486871167 E_h

O	12.00541424069980	14.89079769471808	7.98938921562879
O	14.07606123876390	14.01718405759624	7.97677384538408
O	14.22346990676475	9.19596049823667	9.67792648465546
O	12.21036149420462	8.38959450043160	10.26680075251247
O	12.85748449290577	12.07636848221927	10.15022665609161
H	13.61638197857907	11.73916995465021	10.64429181173398
O	5.53938138595538	7.79556192171008	5.89656142690586
O	7.32591155883531	9.04669834869952	6.42236530720921
O	6.06424112675526	13.41922423089381	4.19030682255197
O	7.58198959434093	11.79983324742772	4.51107395794609

N	10.62262537426282	12.73807170814821	5.01001863263315
N	10.57694549433208	8.26968101962897	6.67626789638164
N	12.01846972632567	10.61638046779579	6.18980919582929
N	10.64996552888710	9.91234230625842	3.66865965618977
N	8.17104005036674	9.06601676475862	3.90965803648374
N	7.71763222376710	12.27356562197772	7.10126884272511
N	10.02658144470921	11.31188728752168	8.37859327876556
C	12.18304079451648	12.51238707872818	7.89140112741165
C	12.46462783955185	12.01153595451485	6.44301522879219
H	13.55587828843865	12.04462656447087	6.35198706323530
C	12.49115311648647	9.67994077730443	7.24728165705151
H	13.57804420250709	9.57435958136581	7.15947361411873
C	12.24902540576564	10.19208263273564	8.69971682921526
C	10.79322750061032	10.30330924249327	9.14340032057713
H	10.27562776187493	9.35167568813246	9.04590123957676
H	10.79279166214815	10.57454037820317	10.20387207940052
C	10.71998199473278	12.62028907258177	8.32006088283286
H	10.69533811463273	13.08625832379787	9.31043551296616
H	10.17035305765010	13.26736292969621	7.64025267750672
C	12.93138158854694	11.56334997347237	8.82525490895550
H	13.97129964341806	11.48430897607174	8.50847285994073
C	12.86833602242898	13.87991224204564	7.97058737128108
C	12.58558676723289	16.22032021596659	7.98685558781954
H	13.18129707097131	16.35443860229869	7.08549116517623
H	13.19974708196848	16.35335165369888	8.87553658691108
H	11.73693861823571	16.89709051261102	7.99589071956341
C	13.00967656228380	9.21602440994412	9.60132191413841
C	12.86982346233008	7.39537061852878	11.09214211200458
H	13.50001604547973	6.76470125104765	10.46746981634890
H	12.06380645392131	6.81996945558623	11.53649216886378
H	13.46347102354666	7.89118220113283	11.85786784024596
C	11.90429001506896	12.91928756909936	5.37673619580808
C	12.71717654170021	13.88129484659187	4.79011724480594
H	13.74654026004004	13.97836735778760	5.10849903351057
C	12.19582982894493	14.68642425779845	3.78609562426800
H	12.81313439368042	15.43737482197712	3.30990772354925
C	10.87941842249672	14.49041986655721	3.39206243525935
H	10.43206137354183	15.07745887661358	2.60144951432203
C	10.13327724572430	13.50808697739617	4.02551359863347
H	9.11135064587396	13.31412083145018	3.73288071318254
C	11.87217266421727	8.31816854295074	7.03565164719948
C	12.62404927497659	7.16849638394473	7.24713011267539
H	13.66914929614634	7.25286462479273	7.51513092256635
C	12.01682651490220	5.92784665310090	7.10330396392312
H	12.58367519397957	5.01971302572732	7.26400426410504
C	10.67727723363580	5.87542917267514	6.73959425340589
H	10.16251268435532	4.93305219512727	6.61046182776922
C	9.99990599911631	7.06636431759590	6.53002873241209
H	8.95863499671048	7.06747645521277	6.23539936919208
C	12.68809425921672	10.17323181149963	4.91949696757325

H	13.67173594045816	10.64097386449578	4.84138850385616
H	12.84650618585618	9.09610076944061	4.99623859618399
C	11.90116439493252	10.38812027618752	3.65892135238890
C	12.46877231547104	10.93044644909708	2.51242175723667
H	13.47412537932678	11.32799677382409	2.54006884943816
C	11.71277155592261	10.95113622962975	1.34606436767037
H	12.11863963797945	11.38512712916170	0.44132971526905
C	10.43877718881004	10.39714855077818	1.33993981482850
H	9.84822301699654	10.38418657501159	0.43481007080003
C	9.93292335905997	9.87875650394667	2.52872965483955
C	8.60238168852176	9.24326874580930	2.65159180882366
C	7.83970166768223	8.82590492518062	1.56185172660263
H	8.19544057679520	8.97012393872995	0.55123108797876
C	6.61987025833018	8.20580897225531	1.80035088014246
H	6.01501177240643	7.86430499879267	0.97018833770767
C	6.18158138868814	8.03015578889510	3.10790666902503
H	5.23490121933443	7.55808446542509	3.32953530677368
C	6.98740810241559	8.49330923558067	4.14139726224488
C	6.56844460639142	8.42397955548892	5.59617835405729
C	8.75836910048788	11.50809729829232	9.12727144455960
H	8.97536494365333	11.87706175809809	10.13514767645000
H	8.27669904995169	10.53087978925570	9.22492723194366
C	7.82380257567101	12.44591210188571	8.42310361903088
C	7.08157704582665	13.41228773504131	9.09406811346757
H	7.19762140677790	13.53646836053033	10.16247395948148
C	6.20345977322221	14.20509891325823	8.36604970546678
H	5.61546967727248	14.96624102911287	8.86298300073370
C	6.09118573036426	14.01483973442098	6.99296971367486
H	5.41927164419391	14.60993173793872	6.39077761714112
C	6.87020737670661	13.03518101519473	6.39449010007540
C	6.81990435406879	12.74468446483548	4.91330232018260
La	9.25008328236057	10.50017727740302	5.85895413420104

Table S53: Cartesian coordinates (Å) obtained from geometry optimizations for $[Ac(BISPI)]^{+}$.

E -2982.678181624762 E_h

O	11.99485967270096	14.88750093286361	7.90264507675838
O	14.06786745169148	14.01971576471405	7.91741890219802
O	14.23985718202064	9.24332853597264	9.72339083016206
O	12.23362656458135	8.42539945200068	10.31949556502247
O	12.86773596724988	12.12184398301476	10.13457600241784
H	13.62746560779227	11.79239977694945	10.63265602022108
O	5.44420147147950	7.66446152642645	5.60301277690050
O	7.20725908902641	8.88429861168018	6.26290729269237
O	5.92118115127620	13.44763587098136	4.33331562511236
O	7.43303423919070	11.81203469356550	4.59675799956524
N	10.59488383644517	12.69956795915746	5.01146166901479
N	10.59245478701466	8.21784522185038	6.76354407443165
N	12.00890995697239	10.57562017868618	6.21082108347648

N	10.65342137217124	9.86916474012163	3.64094496479229
N	8.13999721472654	9.04551067318797	3.77721164913156
N	7.65853564406412	12.28204162277942	7.19027387985091
N	10.02529333939424	11.32016760237586	8.41662291203044
C	12.17847519345270	12.50779139990722	7.87150765172321
C	12.45253577574304	11.97685771695234	6.43026602899247
H	13.54341231233589	12.01115293903556	6.33100434852821
C	12.49510750311995	9.66456212258157	7.28373484536618
H	13.58280412887294	9.56639127516984	7.19188788481956
C	12.25737466537508	10.20575407613235	8.72696783284880
C	10.80415594823111	10.32051468919023	9.18061761042571
H	10.28840164569803	9.36618601340133	9.09818147565471
H	10.81424743502237	10.59972467236875	10.23913579996388
C	10.71821289513032	12.62509925164864	8.31136727342081
H	10.70376916830883	13.12293745655398	9.28645754276952
H	10.16172424979468	13.25121896839367	7.61716605139215
C	12.93546888433304	11.58141597162897	8.82011962363687
H	13.97379842933636	11.49947810135606	8.49883969680807
C	12.86056366203890	13.87868065232981	7.91430505006313
C	12.57106719179350	16.21771556247016	7.85428542005324
H	13.17009994681755	16.32110276767954	6.95100641595103
H	13.18123139701286	16.38492321370407	8.74003772772168
H	11.72033730932507	16.89175355946618	7.83551255678762
C	13.02608014968860	9.25061762493936	9.64417159943647
C	12.90122480777167	7.44795537969722	11.15818771156076
H	13.54146524705542	6.81798660652263	10.54302062139678
H	12.10009750377574	6.86801119880768	11.60557579107699
H	13.48577354271313	7.95909302353159	11.92093789589728
C	11.88385682696916	12.87113815374201	5.35545503418909
C	12.69382217613865	13.82159648864568	4.74629375104858
H	13.72945495442120	13.91360187735893	5.04518861822974
C	12.16084128406242	14.62267691896844	3.74490493734198
H	12.77605178610580	15.36438992719571	3.25172036988326
C	10.83554012280451	14.43679141617893	3.37595168921651
H	10.37937714729164	15.02292678510579	2.58969140476529
C	10.09264543128041	13.46586139586399	4.03072273358211
H	9.06153023064170	13.28092552867608	3.76222114695109
C	11.89034068083406	8.29036570357071	7.10659408261824
C	12.65787860352930	7.15347087093897	7.33064254115487
H	13.70490586408763	7.25409394869171	7.58546730166744
C	12.06297359387226	5.90354656570263	7.21621781344305
H	12.64233627303518	5.00524177927713	7.38760792734639
C	10.72026884746650	5.82729352417784	6.86764434770605
H	10.21660140587656	4.87608140755971	6.76130843605705
C	10.02663244525035	7.00638127534655	6.64396250596560
H	8.98115213951122	6.99267880007567	6.36023908296878
C	12.66736925902512	10.10155360959818	4.94721719678946
H	13.66989663707989	10.53005940530567	4.87649326359235
H	12.78126945249011	9.01966460617783	5.02929750327252
C	11.90430744977154	10.34834050939361	3.67619019879227

C	12.49706282331069	10.93296035683962	2.56345765152708
H	13.50222263217564	11.32660315971878	2.62920820602580
C	11.76730621035585	11.00363323563584	1.38285141074831
H	12.19372809404230	11.47148933323488	0.50483737093617
C	10.49054617816825	10.45983671027354	1.33072246488900
H	9.91659930002350	10.49038872283990	0.41549057557187
C	9.95909439723413	9.89305045905621	2.48612936513260
C	8.61840629525230	9.26758842770854	2.54327260444387
C	7.89308269767596	8.90020431041468	1.41040341683473
H	8.28625222942010	9.08116268410165	0.41984534422178
C	6.66194537199041	8.28004855316344	1.57841442717188
H	6.08538741877918	7.97765648797480	0.71359091351500
C	6.17815229837422	8.04891722206172	2.86102419589306
H	5.22438938967505	7.56906705750315	3.02890477586792
C	6.94849048827392	8.46272603357890	3.94130393843768
C	6.48134810813799	8.31075857615533	5.37537604531615
C	8.77206938894751	11.52887361483791	9.18733703481392
H	9.01148991905520	11.90198880393463	10.18874445693801
H	8.28780120783177	10.55456136690985	9.30257353671480
C	7.82623661304737	12.47250286207237	8.50356427532117
C	7.13776341089466	13.46791062945568	9.18933516996472
H	7.30273860729704	13.60618586268471	10.24960216861321
C	6.24988569203347	14.27231669592755	8.48625121918623
H	5.70241324664083	15.05521196846637	8.99562293677843
C	6.07810806285216	14.06774686624260	7.12154452159097
H	5.40018500287364	14.67439805822825	6.53791646645438
C	6.80690959510293	13.06015944114812	6.50577541037457
C	6.69688720171088	12.76535084889708	5.02725720841416
Ac	9.16763196673158	10.43655976358409	5.87756022164622

References

(1) Aime, S.; Barge, A.; Benetollo, F.; Bombieri, G.; Botta, M.; Uggeri, F. A Novel Compound in the Lanthanide(III) DOTA Series. X-Ray Crystal and Molecular Structure of the Complex Na[La(DOTA)La(HDOTA)]·10H2O. *Inorg. Chem.* **1997**, *36* (19), 4287–4289. https://doi.org/10.1021/ic9704501.

(2) Thiele, N. A.; Brown, V.; Kelly, J. M.; Amor-Coarasa, A.; Jermilova, U.; MacMillan, S. N.; Nikolopoulou, A.; Ponnala, S.; Ramogida, C. F.; Robertson, A. K. H.; Rodríguez-Rodríguez, C.; Schaffer, P.; Williams Jr., C.; Babich, J. W.; Radchenko, V.; Wilson, J. J. An Eighteen-Membered Macrocyclic Ligand for Actinium-225 Targeted Alpha Therapy. *Angewandte Chemie International Edition* **2017**, *56* (46), 14712–14717. https://doi.org/10.1002/anie.201709532.

(3) Valencia, L.; Martinez, J.; Macías, A.; Bastida, R.; Carvalho, R. A.; Geraldes, C. F. G. C. X-Ray Diffraction and 1H NMR in Solution: Structural Determination of Lanthanide Complexes of a Py2N6Ac4 Ligand. *Inorg. Chem.* **2002**, *41* (20), 5300–5312. https://doi.org/10.1021/ic0257017.

(4) Jaraquemada-Peláez, M. de G.; Wang, X.; Clough, T. J.; Cao, Y.; Choudhary, N.; Emler, K.; Patrick, B. O.; Orvig, C. H4octapa: Synthesis, Solution Equilibria and

Complexes with Useful Radiopharmaceutical Metal Ions. *Dalton Trans.* **2017**, *46* (42), 14647–14658. https://doi.org/10.1039/C7DT02343J.
(5) Freire-García, A.; Babi Araujo, Y.; Wuest, M.; Szilágyi, B.; Madarasi, E.; Valencia, L.; Argibay-Otero, S.; Rodríguez-Rodríguez, A.; Esteban-Gómez, D.; Tircsó, G.; Wuest, F.; Platas-Iglesias, C. Decadentate Acyclic Chelators for Lanthanum Radiopharmaceuticals. *J. Med. Chem.* **2025**, *68* (16), 17823–17839. https://doi.org/10.1021/acs.jmedchem.5c01558.
(6) Gao, S.; George, S. J.; Zhou, Z.-H. Interaction of Gd-DTPA with Phosphate and Phosphite: Toward the Reaction Intermediate in Nephrogenic Systemic Fibrosis. *Dalton Trans.* **2016**, *45* (12), 5388–5394. https://doi.org/10.1039/C5DT04172D.
(7) Rui-Yao Wang; Jun-Ran Li; Tian-Zhu Jin; Guang-Xian Xu; Zhong-Yuan Zhou; Xiang-Ge Zhou. Synthesis and Crystal Structure of the Potassium Lanthanum Complex with Triethylenetetra-Aminehexaacetic Acid (H6TTHA): K[KLa(HTTHA)(H2O)]·8H2O. *Polyhedron* **1997**, *16* (8), 1361–1364. https://doi.org/10.1016/S0277-5387(96)00400-7.
